# Magnetic Anisotropy Dominates over Physical and Magnetic Structure in Performance of Magnetic Nanoflowers

**DOI:** 10.1002/sstr.202400410

**Published:** 2024-09-30

**Authors:** Julie Borchers, Kathryn Krycka, Brianna Bosch-Santos, Eduardo de Lima Correa, Anirudh Sharma, Hayden Carlton, Yanliu Dang, Michael Donahue, Cordula Grüttner, Robert Ivkov, Cindi L. Dennis

**Affiliations:** NIST Center for Neutron Research, National Institute of Standards and Technology Gaithersburg, MD 20899-6102, USA; NIST Center for Neutron Research, National Institute of Standards and Technology Gaithersburg, MD 20899-6102, USA; Material Measurement Laboratory, National Institute of Standards and Technology, Gaithersburg, MD 20899-8552, USA; Material Measurement Laboratory, National Institute of Standards and Technology, Gaithersburg, MD 20899-8552, USA; Theiss Research, La Jolla, CA 92037, USA; Department of Radiation Oncology and Molecular Radiation Sciences, Johns Hopkins University School of Medicine, Baltimore, MD 21231, USA; Department of Radiation Oncology and Molecular Radiation Sciences, Johns Hopkins University School of Medicine, Baltimore, MD 21231, USA; Material Measurement Laboratory, National Institute of Standards and Technology, Gaithersburg, MD 20899-8552, USA; Information Technology Laboratory, National Institute of Standards and Technology, Gaithersburg, MD 20899, USA; micromod Partikeltechnologie, GmbH, 18057 Rostock, Germany; Department of Radiation Oncology and Molecular Radiation Sciences, Johns Hopkins University School of Medicine, Baltimore, MD 21231, USA; Department of Oncology, Sydney Kimmel Comprehensive Cancer Center, Johns Hopkins University School of Medicine, Baltimore, MD 21231, USA; Department of Mechanical Engineering Whiting School of Engineering, Johns Hopkins University, Baltimore, MD 21218-2681, USA; Department of Materials Science and Engineering, Whiting School of Engineering, Johns Hopkins University, Baltimore, MD 21218-2681, USA; Material Measurement Laboratory, National Institute of Standards and Technology, Gaithersburg, MD 20899-8552, USA

**Keywords:** magnetic anisotropy, magnetic nanoparticles, nanoflowers, small angle neutron scattering

## Abstract

Magnetic nanoparticles are indispensable in many biomedical applications, but it remains unclear how the composition and structure will influence the application specific performance. We consider two compositions, ferrite and cobalt ferrite, synthesized under conditions that create aggregated multi-core nanoparticles, called nanoflowers. Each nanoflower has an ionic surfactant or dextran to provide colloid stability in water. The composition, but not the coating, greatly impacts the heating output and the magnetic particle imaging tracer quality (with cobalt ferrite significantly reduced compared to ferrite). The cobalt ferrite nanoflowers have a core/shell structure with a reduced magnetization, which limits the effective magnetic anisotropy of the individual cobalt ferrite nanoflowers as well as the magnetic interactions among the nanoflowers. Both limitations significantly reduce the overall increase in the magnetic anisotropy with increasing magnetic field and consequently the nanoflowers’ efficacy for heating and imaging. Despite this, the formation of denser-packed clusters and chains with external magnetic field in the ionic surfactant-cobalt ferrite nanoflowers overcomes some of the shell’s detrimental effects, resulting in better heating and imaging properties compared to the dextran-cobalt ferrite. In short, the magnetic anisotropy dominates over physical and magnetic structure in the performance of the studied nanoflowers for heating and imaging applications.

## Graphical Abstract

Idealized schematics showing example colloidal structures, consistent with the SANS data, that form in the magnetic nanoflower (MNF) colloids. The chains increase the overall magnetic anisotropy which controls the MNFs’ effectiveness for Magnetic Particle Imaging and Magnetic Nanofluid Hyperthermia. This anisotropy increase can overcome other detrimental effects like shell formation, but, if the increase is too large, may become detrimental.

## 1. Introduction

Magnetic nanoparticles (MNPs) have demonstrated utility in many research and commercial applications. Among the various MNPs developed, magnetic iron oxide nanoparticles have established uses for magnet-assisted cell sorting, diagnostic imaging, drug delivery, and magnetic nanoparticle hyperthermia (MNPH) to treat solid tumor malignancies.^[1–11]^ More recently, iron oxide nanoparticles can act as agents to treat iron deficiency anemia and are recognized as potential vaccine adjuvants or immune therapies to treat infectious diseases and cancer.^[12–16]^ Early evidence suggests that the combination of the presence of iron and other physicochemical properties of the nanoparticles (such as size, shape, coating, etc.) offer the potential to stimulate innate immune effector function and initiate adaptive immune responses. The iron oxide nanoparticles do not have to be magnetic, but magnet-responsive nanoparticles enable magnet-assisted immune cell sorting to interrogate changes to tumor immune microenvironments^[12,17]^ or MNPH.^[15,18–20]^ Perhaps the most exciting prospect for this class of nanomaterials is that with appropriate design of physical composition and magnetic properties, multifunctional diagnostic and therapeutic, or “theranostic”, applications are possible.

The theranostic potential offered by combining magnetic particle imaging (MPI) with MNPH has motivated development of a specific type of magnetic iron oxide nanoparticle capable of performing well for both MNPH^[21]^ and MPI.^[22]^ MNPH is a cancer treatment modality that exploits heat stress to sensitize cancer cells to radiation therapy or chemotherapies by inhibiting or abrogating their DNA damage repair mechanisms. Cancer cell killing depends upon duration of exposure to elevated temperature between 42 °C and 47 °C for about 30–60 min. Heat is generated within the tumor by MNPs when the region is exposed to an alternating magnetic field (AMF). The efficiency of heating, which determines therapeutic “potency”, arises from hysteresis losses due to reorientation of the spins within the MNP from the applied AMF.^[21]^ Intratumor heating can be controlled remotely by adjusting the amplitude (and potentially the frequency) of the AMF, which penetrates tissue with little attenuation.^[23,24]^ MNPH also offers the potential to precisely treat deep-seated tumors to a prescribed thermal dose, that is, time-at-temperature.^[20,24]^ However, as the MNPs are the source of heat, their spatial distribution within the target tissue determines temperature distribution and effectiveness as a cancer treatment, making it essential to combine MNPH treatment planning with an imaging modality.^[5]^

Imaging iron oxide MNPs within tissues has proven to be challenging with clinical imaging modalities. MRI for MNPH is unreliable because MNP concentrations required for MNPH generate significant magnetic susceptibility and other artifacts. In addition, MNPs are T2 contrast agents for MRI, yielding a hypointense signal that is indistinguishable from natural hypointense features often encountered in tissue. Iron oxide MNPs provide limited radiodensity that can be used to confirm MNP presence in tissue with clinical CT imaging, but this radiodensity is generally insufficient to enable treatment planning.^[25,26]^ In contrast, MPI creates images from the measured non-linear magnetic response of MNPs exposed to an AMF operating at a low frequency.^[22,27–30]^ For MPI, MNPs are tracers that enable detection at both low concentration (<1 × 10^−8^ g of MNP per g of tissue) for diagnostic imaging and high concentration (>1 × 10^−2^ g of MNP per g of tissue) for MNPH treatment planning.^[5]^

The magnetic performance of iron oxide MNPs for applications requiring magnetic fields is typically assumed to depend upon their chemical structure (i.e., maghemite vs magnetite) and single-crystal, single-core MNP anisotropy energy.^[31]^ Contemporary research demonstrates that more complex MNP nanocrystalline structure(s) and interparticle interactions may substantially influence their magnetic responses and performance for MPI and MNPH.^[32–35]^ In particular, MNPs comprised of crystallites, grains, and/or clusters excel at MNPH with low AMF amplitudes^[36–39]^ because nanometer-scale spin disorder at the crystallite boundaries within grains, and dipolar/exchange coupling among grains modulate energy and time-dependent responses of their magnetization.^[35]^ Details of how these magneto-structural features influence MPI performance remain unclear.^[21]^ Gavilan et al. demonstrated that nanoflower MNPs could be synthesized^[34]^ to have a dense structure comprised of misaligned crystalline grains. Depending on the synthetic conditions, the degree of crystallographic (mis)alignment can be controlled, as can the size and shape of the grains and the nanoflower MNP. Such a complex structure may directly affect the dynamic response, and therefore the heating and MPI performance of nanoflowers.

Interparticle interactions also influence performance for MNPH and MPI. While the intraparticle magnetic structure may define the effective magnetic anisotropy of an individual MNP, the physical structure and state, composition, and concentration of MNPs in the medium determine their contributions to interparticle interactions, which also influence the magnetic anisotropy. These MNP interactions can produce complex spatial arrangements or chain formation when the MNPs are suspended in liquid media. These formations directly affect aggregate sample responsiveness to AMFs for both heating and imaging. Indeed, some MNP arrangements can yield collective magnetic behaviors that directly influence hysteresis heating for MNPH.^[40–43]^ For a given MNP composition, these interparticle interactions can be sensitive to the presence and type of MNP coating which influence the collective structure of the aggregate.^[44]^

Our objective here is to ascertain the relative impact of interparticle interactions, composition, and magnetic anisotropy on performance. We compare physical, colloidal, and magnetic structures; static and dynamic magnetization; magnetic particle imaging and heating performance among four nanoflower MNPs. The ferrite nanoflowers (labeled FeNF) considered in this investigation comprise iron oxide grains with electrostatic stabilization (-E) or dextran surface coatings (-D). To determine the relationship between their structure and response to AMFs, we used transmission electron microscopy (TEM), X-ray diffraction (XRD), dynamic light scattering (DLS), AC susceptibility (ACS), torque magnetometry, polarized small angle neutron scattering (SANS), specific loss power (SLP) measurements, point spread function (PSF) in a commercial small animal preclinical MPI scanner, as well as magnetization versus applied magnetic field (*M* vs *H*) and magnetization versus temperature (*M* vs *T*) measurements. We performed comparable measurements using cobalt ferrite nanoflowers (labeled CoNF), similarly coated, to ascertain if the observed trends are enhanced with increased magnetic moment and/or magnetocrystalline anisotropy.

For the two applications we tested, heating (SLP) and MPI tracer quality (PSF), we measured surprisingly little difference between the coated and uncoated FeNFs. In contrast, the SLP and PSF of the CoNFs are significantly lower than that of the FeNFs, with CoNF-D exhibiting the greatest reduction in heating and MPI tracer performance. To explain these pronounced differences, we probed the complex aggregate structures that form in the colloids in response to a static magnetic field. The nano-flower coating influences the shape and size of clusters and chains that develop. Specifically, the electrostatically stabilized MNFs form multi-pole-like clusters that merge into chains or narrow columns when exposed to an increasing magnetic field. The dextran-stabilized MNFs form dipoles that create branched chains as the magnetic field increases. Both FeNFs display comparable increases in the effective magnetic anisotropy (from chaining) with increasing magnetic field. Measurable hysteresis losses persist for heat generation (SLP) because the value of the effective magnetic anisotropy does not exceed the peak amplitude of the AMF used. Furthermore, both FeNFs display magnetic anisotropy properties suitable for MPI. In comparison, the magnetic anisotropy measured for both CoNFs is significantly reduced, especially for CoNF-D. Our analysis of data, however, suggests that the sensitivity of CoNF performance to the coating was due to the presence of a stiff magnetic outer shell surrounding the nanoflower core that produces a rigid, frustrated magnetization that resists rotation. Our analysis supports a hypothesis that closer packing of CoNF-E likely facilitates longer-range magnetic interactions that partially overcome the frustrations to enable moment rotation, yielding better PSF and SLP performance. Overall, our analysis highlights the importance of magnetic anisotropy, relative to surface stabilization and interparticle structure, in determining the function of these complex MNP systems, and suggests future directions to improve performance with minor alterations to their synthesis and coating.

## 2. Results

Many of the magnetic nanoparticles synthesized for biomedical applications have a single-crystal core composed of a single material (e.g., magnetite), which is often approximately spherical in shape and has a surface stabilization (e.g., polymer). Recently, interest has developed in multi-crystalline cores, so-called “nano-flowers” because each crystalline grain looks like the petal on a nano-flower. Here, we consider the performance of two different compositions of nanoflowers, each with a different surface stabilization (Table 1). The FeNF-E nanoflowers are composed of iron oxide with no additional surface stabilization beyond the electrostatic coating developed during synthesis. The FeNF-D nanoflowers are the same as the FeNF-E but with an additional physisorbed coating of dextran on the surface. (The nanoflower cores are from the same batch.) The CoNF-E nanoflowers are composed of cobalt iron oxide with no additional surface stabilization beyond the electrostatic coating developed during synthesis. As for FeNFs, the CoNF-D nanoflowers are the same as the CoNF-E but with an additional physisorbed coating of dextran on the surface. (The cores are from the same batch.)

**Figure 1.**
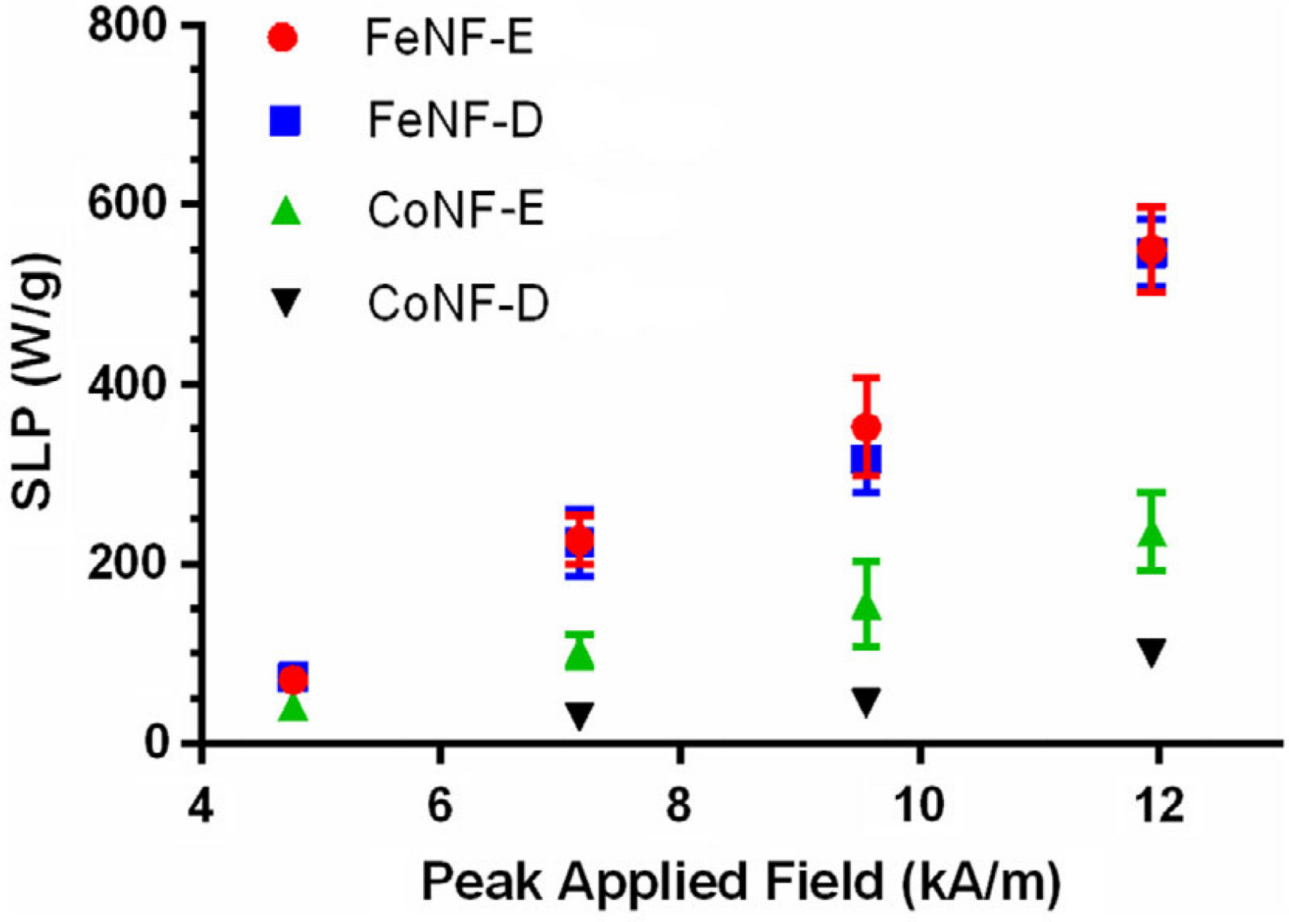
Measured SLP versus AMF peak amplitude for FeNF-E, FeNF-D, CoNF-E, and CoNF-D. SLP was estimated from calorimetry data measured with an AMF frequency of 340 kHz for all MNFs. Data shown represent mean SLP values and error bars represent one standard deviation.

In MNPH, the performance is determined by the SLP, which is the heat generated per mass of material, as a function of the peak amplitude AMF (Figure 1). All the magnetic nanoflowers (MNFs) exhibit a monotonically increasing SLP with peak amplitude AMF. Despite the difference in coating, the SLPs for FeNF-E and FeNF-D are within measurement variance of each other (See also Figure S8, Supporting Information). In contrast, the coating significantly impacts the SLP for the CoNFs; uncoated CoNF-E show a lower SLP than either of the FeNFs but display a two to three times larger SLP than that measured for CoNF-D nanoparticles.

**Figure 2.**
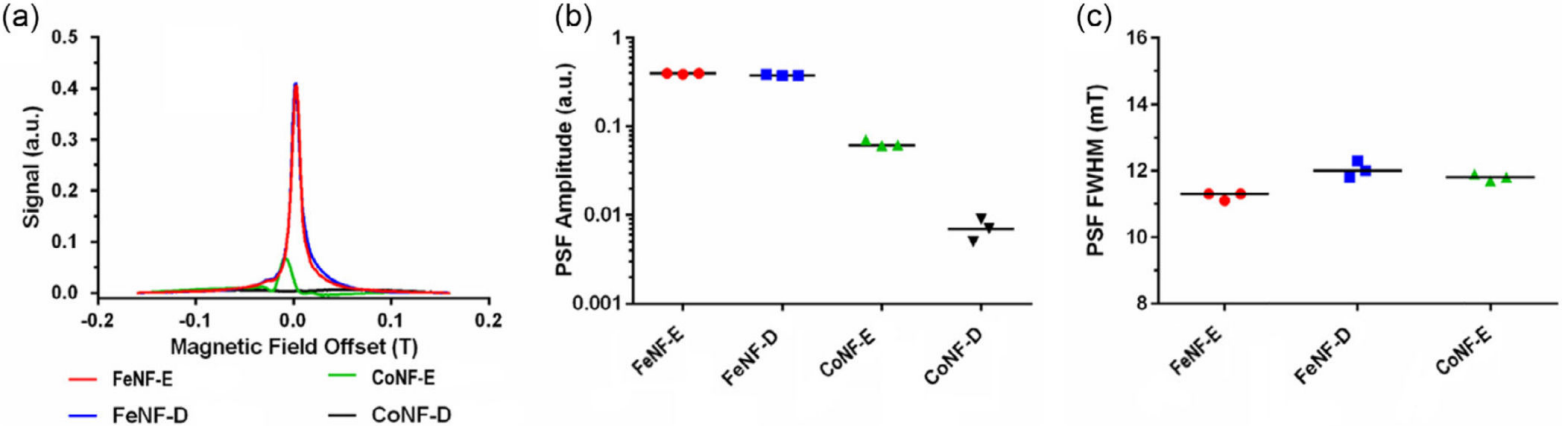
PSF measurements for FeNF-E, FeNF-D, CoNF-E, and CoNF-D. a) Representative comparison of the PSF for each MNF. PSF b) amplitude and c) FWHM for each MNF. (The peak for CoNF-D was not distinct enough to measure the FWHM.) Data shown represent median values and error bars represent one standard deviation.

In MPI, two aspects of the performance are indicated by the PSF (Figure 2a). The signal is proportional to the amplitude of the PSF (Figure 2b), so a larger amplitude correlates with a stronger signal (i.e., a “brighter” image). The full width at half maximum (FWHM) corresponds to the spatial resolution (Figure 2c), with a narrower width corresponding to sharper resolution. As is observed with SLP, the measured PSF of the FeNFs is unaffected by coating, whereas the coating of the CoNFs results in measurable differences. In addition, PSF from both CoNFs is reduced compared to the FeNFs. Specifically, the amplitude of the PSFs obtained from both FeNFs are indistinguishable within measurement uncertainty, and the FWHM of FeNF-E is slightly less than that measured from FeNF-D. The amplitude of the PSF obtained from CoNF-E is nearly tenfold lower than that measured from either FeNFs, and the PSF amplitude of CoNF-D is nearly one order of magnitude lower still in comparison to CoNF-E. Interestingly, the FWHM of CoNF-E is similar to that of FeNF-D but could not be measured in CoNF-D as the peak is indistinct. (See also Figure S9, Supporting Information, for data from a complementary sample set.)

In short, in both performance applications the FeNFs behave similarly despite the different coatings, while the coating influences performance of the CoNFs. Furthermore, the CoNFs display a consistently worse performance than do FeNFs for both heating and imaging applications. The objective of our further characterization of these nanoflower formulations is to better understand the origin of the differences.

**Figure 3.**
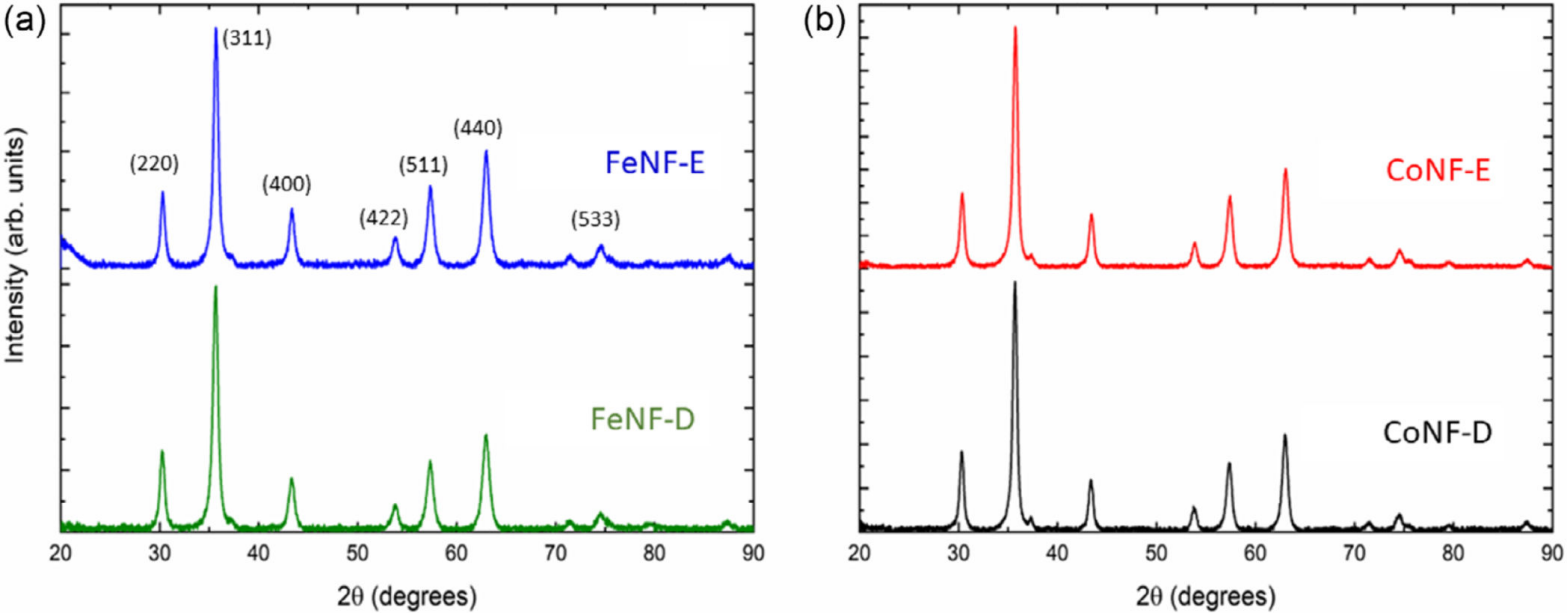
XRD of the a) FeNF-E and FeNF-D and b) CoNF-E and CoNF-D nanoflowers. Peak labels in (a) are the same for all scans. Direct comparison of the data for all four MNF samples, including a close-up of the primary (311) peak showing the slight shift in position between the CoNFs and FeNFs, can be found in Figure SI-13.

**Figure 4.**
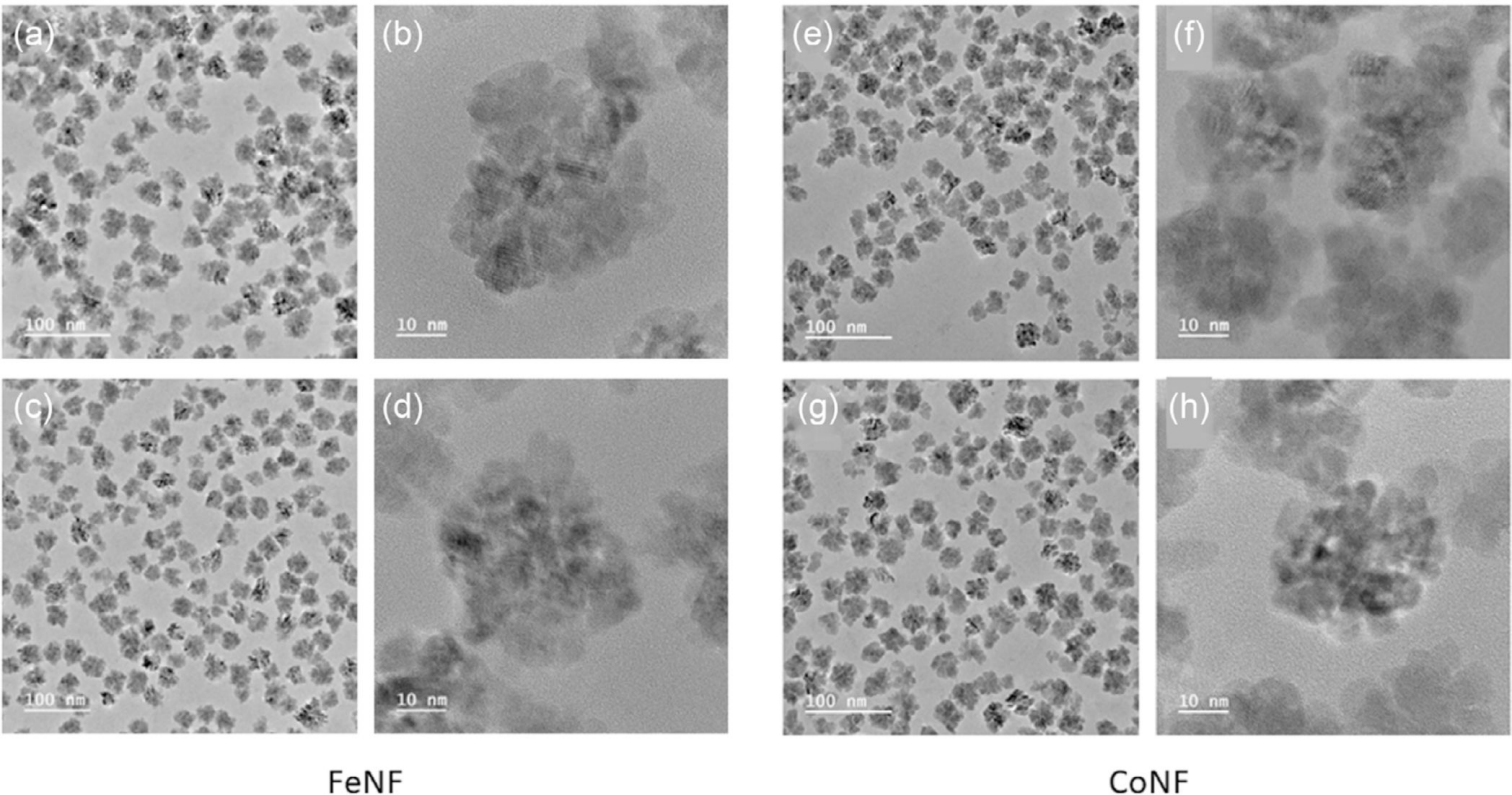
TEM image of a) FeNF-E, including a close-up in b), as well as c) FeNF-D, including a close-up in d). TEM image of e) CoNF-E, including a close-up in f), as well as g) CoNF-D, including a close-up in h).

First, we describe the physical characterization of the four colloids. Analysis of XRD results (Figure 3a) shows that both FeNFs were comprised of grains displaying inverse spinel structure, likely a mixture of maghemite (γ-Fe_2_O_3_) and magnetite (Fe_3_O_4_). The smallest dimension of the nanocrystal grains was ≈15 nm for FeNF-E and ≈13 nm for FeNF-D.^[34]^ Groups of nanocrystal grains clustered to form each MNF, as shown in the TEM (Figure 4a–d). Nanoflowers in both FeNFs appear to be distorted spheres with rough surface features, or ellipsoids of ≈26 nm–30 nm in diameter (Figure 4a,c). TEM confirms the approximate grain size determined by XRD. Furthermore, each grain within the MNFs appears to be a single crystal, but grains are generally crystallographically misaligned with neighboring grains. From DLS measurements (Figure S1 and Table S1, Supporting Information) we interpret that the hydrodynamic diameter of the nanoflowers was 45 ± 3 nm for FeNF-E and 59 ± 3 nm for FeNF-D. Zeta-potential measurements indicate a slight negative charge on the surface of the uncoated FeNF-E particles, resulting in electrostatic stabilization; whereas, FeNF-D shows neutral charge consistent with steric stabilization with dextran. By comparison, the larger hydrodynamic diameter measured for FeNF-D with DLS is consistent with expectations for dextran coating in water. This reasoning, however, does not explain the discrepancy between TEM (30 nm) and DLS (45 nm) measurements for FeNF-E.

Similar structural trends were observed for the CoNFs. XRD results (Figure 3b) for both CoNFs show that they were comprised of grains of cobalt ferrite, with the smallest dimension grain size of ≈10 nm. As before, the grains cluster together to form a core that is ≈25 nm in diameter, as measured by TEM (Figure 4e–h). The CoNFs are generally spherical in shape, and the individual grains in the MNFs appear as single crystals, but without any crystallographic alignment with neighboring grains. As with the FeNFs, the diameter measured from CoNF-E with TEM (≈25 nm) is significantly smaller than the measured hydrodynamic diameter with DLS (44 ± 6 nm; see Figure S1 and Table S1, Supporting Information). The hydrodynamic diameter from DLS is 55 ± 4 nm for CoNF-D, with the difference likely due to the dextran coating.

**Figure 5.**
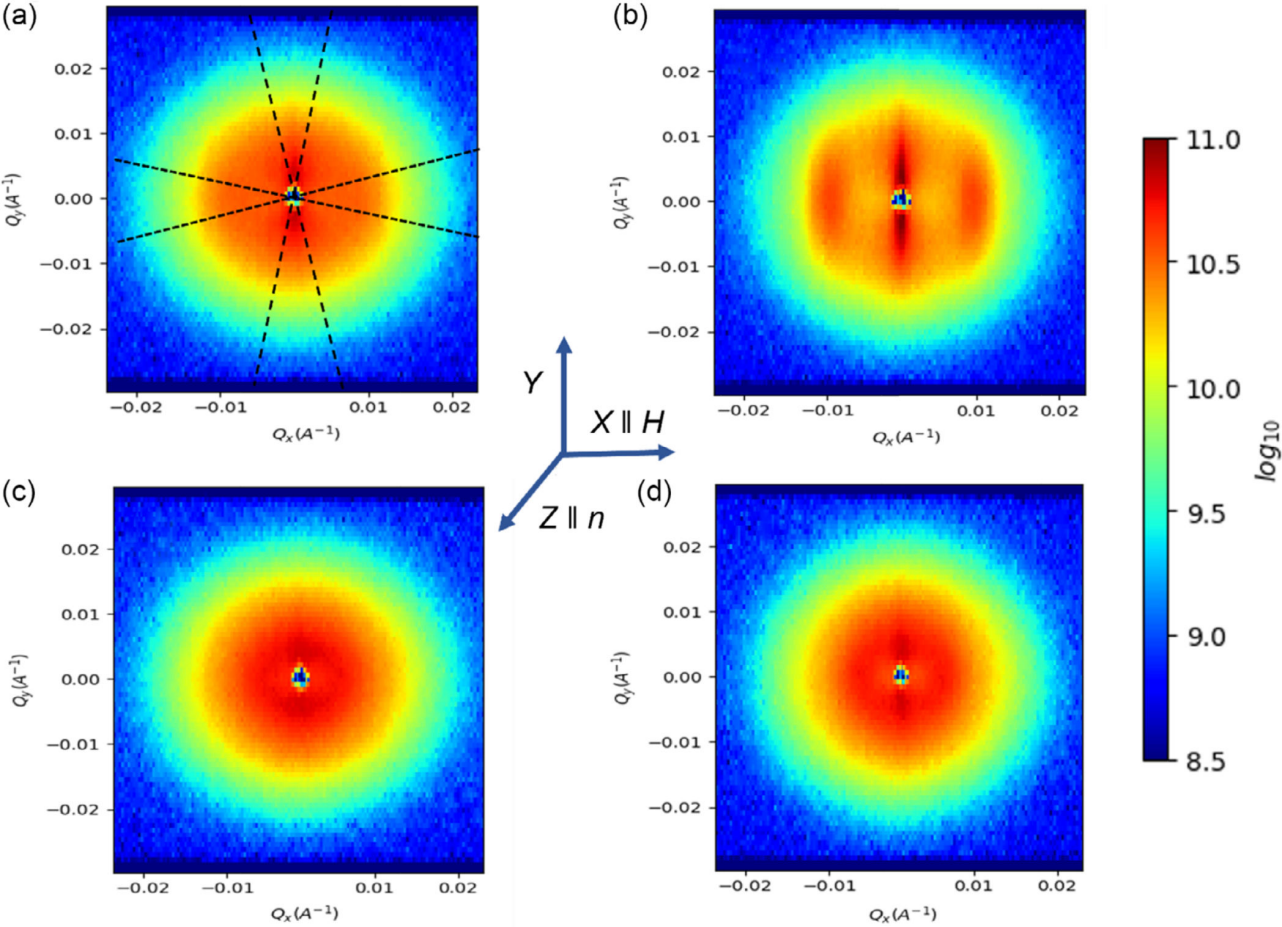
Structural 2D SANS measurements ($I^{↑}+I^{↓}$ from half-polarized scattering) at room temperature for a) FeNF-E in a 7 mT field, b) FeNF-E in a 500 mT field, c) FeNF-D in a 7 mT field, and d) FeNF-D in a 500 mT field. In (a) the dashed lines demonstrate the sector cut parallel to the *Y*-axis and the dotted lines illustrate the sector cut parallel to the *X*-axis. The inset in the middle indicates the different directions. The applied magnetic field H is parallel to the *X*-axis. The neutron beam (*n*) is parallel to the *Z*-axis.

**Figure 6.**
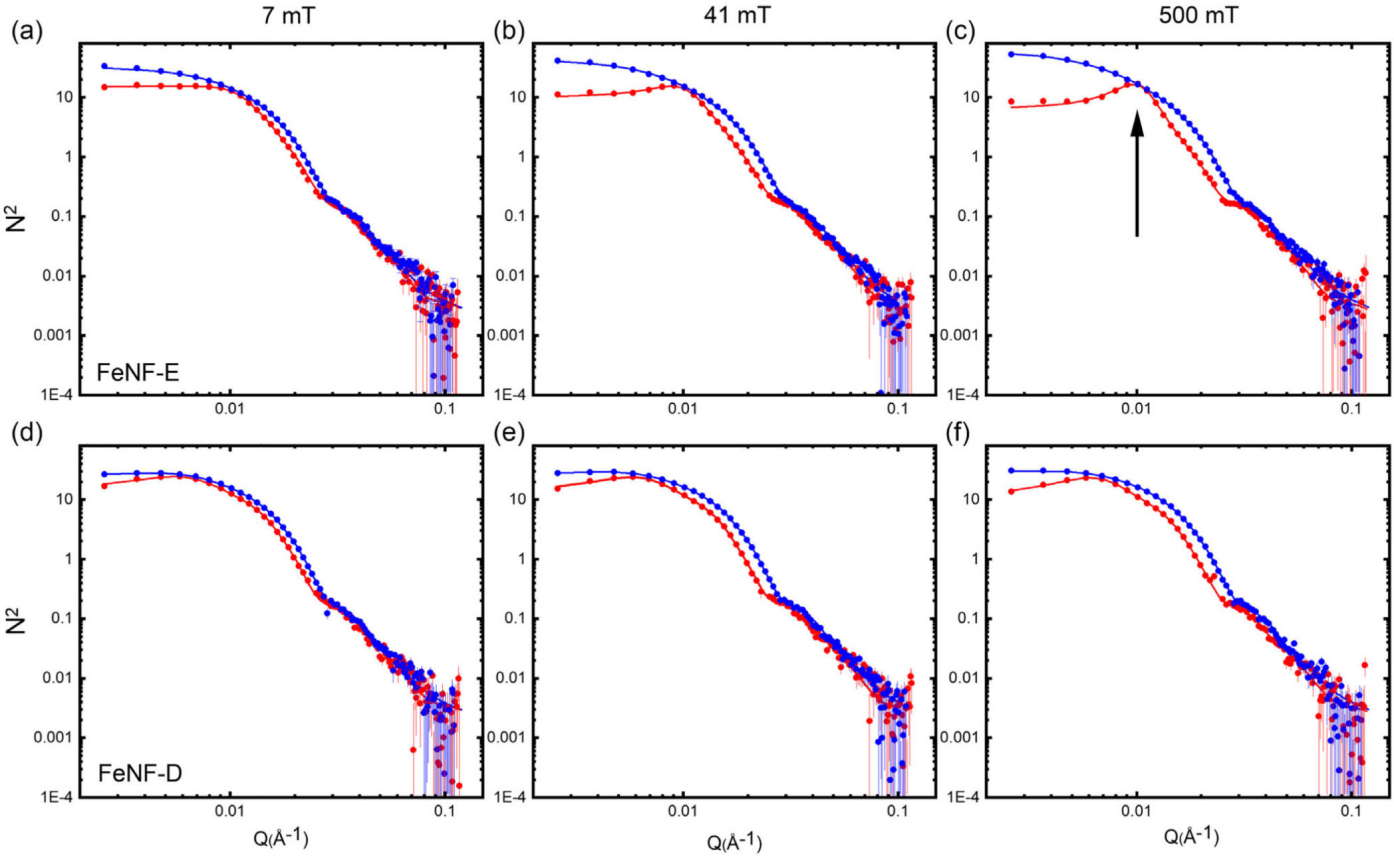
Structural SANS measurements ($I^{↑}+I^{↓}$ from half-polarized scattering) and corresponding fits at room temperature for (top row, a-c) FeNF-E and (bottom row, d-f) FeNF-D in magnetic fields of (left column, a and d) 7 mT, (center column, b and e) 41 mT, and (right column, c and f) 500 mT. Red, blue points are the structurally dominated scattering as seen along directions parallel and perpendicular (i.e., along the *X*- and *Y*-axis), respectively, to the magnetic field which is in the *X* direction. The lines are fits to the data using the model described in the Experimental Methods section. The arrow marks a correlation peak that is a signature of long-range order. Error bars are shown and represent 1*σ*, but may be smaller than the symbol.

We used SANS to focus on the structure of the nanoflowers dispersed in H_2_O, both internal to each nanoflower and aggregated among the interacting nanoflowers (see the Experimental Methods section for more information). As part of this analysis, we also examined how the colloidal structure changes with exposure to an increasing static magnetic field, as the formation of MNP superstructures has been correlated^[45]^ with hysteresis losses and increased SLP. Despite the similarities in nanoflower size and shape (Figure 4), analysis of SANS data reveals differences in the colloidal structure among all samples. As described in the Experimental Methods section, the structural scattering (N^2^) can be characterized by adding the I^↑^ and I^↓^ cross sections of the half-polarized data. An obvious asymmetry in the resulting 2D SANS images (Figure 5), which develops with increasing DC magnetic field, is especially prominent for FeNF-E in a 500 mT field (Figure 5b). This can be more readily quantified with *Q*-dependent sector slices (see dashed and dotted lines in Figure 5a) for both FeNF-E (top) and FeNF-D (bottom) in fields of 7 mT (left), 41 mT (center), and 500 mT (right), which are shown in Figure 6. In the low 7 mT field, the scattering perpendicular to the field (along the *Y*-axis) for both FeNF-E and FeNF-D (blue symbols in Figure 6a,d) is reminiscent of a simple form factor for a sphere, ellipse, or related shape, with no obvious peak indicative of correlations extending beyond a single scattering unit. (The actual fits, described below, do require a slightly more complex model.) This shape remains essentially unchanged when FeNF-D isexposed to high field (blue symbols in Figure 6f), but the high field produces a slight upturn at low *Q* for FeNF-E (blue symbols in Figure 6c), suggesting emergence of longer length scale structures. In contrast, the scattering parallel to the field along the *X*-axis (red symbols) undercuts the perpendicular scattering for both samples at all fields, with the sharp upturn in the data occurring at lower *Q* ≈ 0.015 Å^−1^, suggesting slightly larger structures oriented parallel to the field. Longer-range structural correlations among the nanoparticles in this direction are evidenced by the appearance of a peak in the low-field data (red symbols in Figure 6a,d) at *Q* ≈ 0.010 Å^−1^ and *Q* ≈ 0.065 Å^−1^, respectively, for FeNF-E and FeNF-D. This correlation peak becomes more pronounced for both samples in high field (red symbols in Figure 6c,f), though it is particularly well-defined for FeNF-E (arrow in Figure 6c).

**Figure 7.**
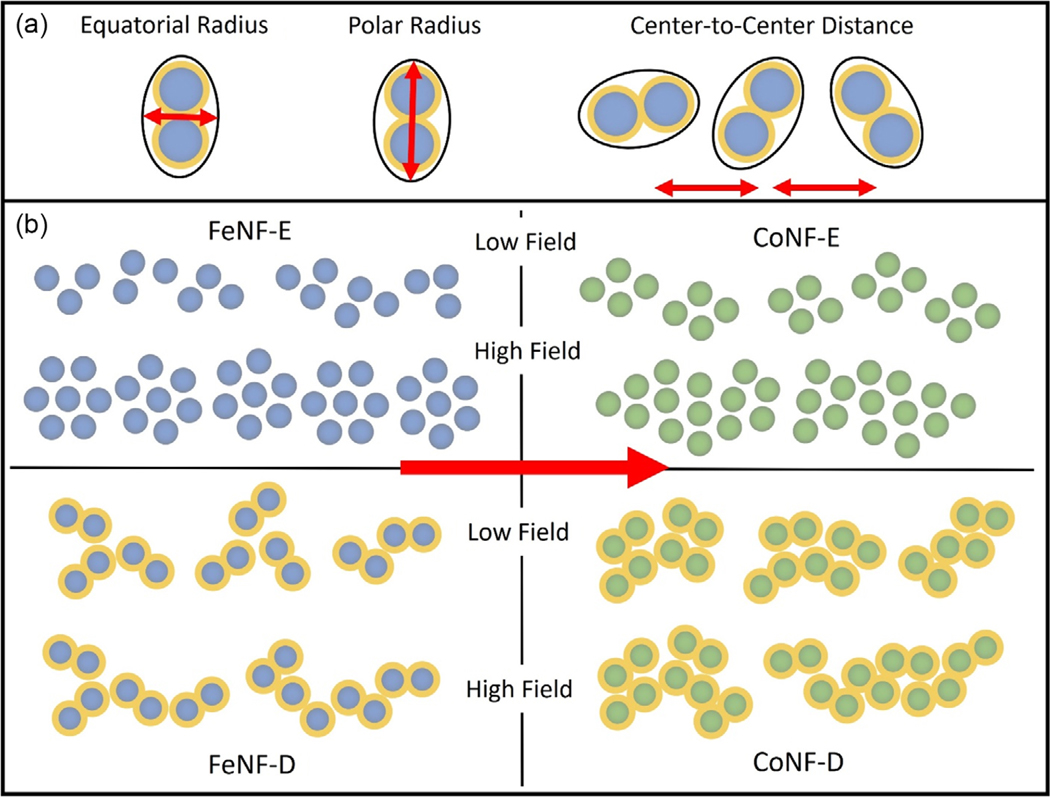
a) Schematic showing the relationship between the ellipsoid dimensions extracted from the fits to the structural SANS data relative to the MNF clusters that form. (Note that this schematic is a 2D representation of a 3D structure.) The case of a nanoflower dipole is shown as an example. b) Idealized schematics showing the colloidal structures, consistent with the SANS fits, that are formed in FeNF-E, FeNF-D, CoNF-E, and CoNF-D nanoflowers at low (70 mT) and high (500 mT) magnetic fields. The yellow ring in the coated nanoparticles represents the dextran shell. The red arrow designates the applied magnetic field direction.

Fitting the data (lines in Figure 6) to an empirical model (see the Experimental Methods section for more information) reveals magnetic field-dependent intra- and inter-particle interactions in these systems. As a reminder, the three-component model chosen to fit the data approximates the nanoflower colloid with 1) an ellipse form factor describing nanoflowers or nanoflower clusters multiplied by 2) a hard-sphere structure factor, characterizing center-to-center distances and long-range order among the clusters (i.e., describing the correlation peaks that emerge in Figure 6), that is added to 3) a spherical form factor that approximates scattering from unattached or otherwise isolated grains. (Refer to Figure S4, Supporting Information, for an example of the breakdown of the contributions of these individual components to a typical fit.) In the context of this model, the best fit parameters for the data in Figure 6 are listed in Table 2. Using the field-dependent ellipse sizes (polar and equator radii), center-to-center distances (twice the hard-sphere radius), and relative degree of chaining (proportional to volume fraction) extracted from the fits to the SANS data, we propose example Structures that are consistent with the data as a function of applied magnetic field (Figure 7). Note that the scale factors multiplying the scattering from the isolated grains are consistently larger along the *X*-axis, suggesting a preferential accumulation of these small structures along the applied field direction for both samples. However, they are not explicitly included in Figure 7.

In Table 2, the equator and polar radii refer to the fitted dimensions of the nanoflowers (or nanoflower clusters) along the ellipsoid center width and length, respectively (Figure 7a). The equator radius for scattering along the Y-axis at all fields is on average 13.3 nm for FeNF-E and 13.6 nm for FeNF-D, approximately corresponding to the 26–30 nm nanoflower diameter obtained from TEM. It is notable that this fitted dimension is larger (average of 15.5 nm) for both samples along the *X*-axis, possibly indicating tilting of the nanoflowers along the field direction (or related to tighter stacking in the third dimension). By contrast, the polar radii (i.e., length) for the ellipsoids are significantly larger than the equator radii (i.e., width). Specifically, the length of the ellipsoid for FeNF-D is approximately double (average of 25.4 and 27.7 nm along the *Y*- and *X*-axes, respectively) its width in both high and low fields. This indicates that the nanoflowers form stable, randomly oriented, two-particle dipole clusters in H_2_O, likely due to dipole interactions (Figure 7b). FeNF-E forms slightly larger clusters (27.8 nm polar radius) along the *Y*-axis in the 7 mT field, but this polar dimension increases to 43.5 nm (>3 times greater than the equator radius) in the 500 mT field. Along the field direction (*X*-axis), the ellipsoid length is also approximately a factor of three greater than the corresponding width in both high and low fields. These results indicate that the structures (likely trigonal at low field and hexagonal at high field) formed by FeNF-E are larger than those formed by FeNF-D (Figure 7b). This tripole formation could account for the larger measured MNF dimensions from DLS (Refer to Table S1, Supporting Information), which would approximate the tripole structures as equivalent spheres of larger radius. The longer lengths in both FeNF samples indicate that these clusters likely form during the synthesis.

Other clues to the contrasting magnetic behavior of these FeNF systems are revealed by the fitted results obtained for estimates of the volume fraction and hard-sphere radius (Table 2), which together parameterize the structure factor and any corresponding long-range correlations among the individual nanoflowers and/or nanoflower clusters. Specifically, the center-to-center distance (i.e., corresponding to twice the hardsphere radius in Table 2 as shown in Figure 7a) between the FeNF-D ellipsoids appears to be independent of direction (*X* or *Y*) and field magnitude. The field-averaged value of 86 nm is almost three times larger than the diameter of the nanoflower ellipsoid along its full width, which is 27–32 nm, but is closer in size to the ellipsoid along its full length, which is 50–58 nm (Table 2). After taking into account the thickness of the surfactant coating, we speculate that some ellipsoid clusters are loosely arranged end-to-end, like dipoles (Figure 7b). While this alignment is present perpendicular to the field (*Y*-axis), it is more pronounced along the field direction (*X*-axis), with a maximum volume fraction of 0.13 obtained for the sector slice along the *X* direction in a field of 500 mT (Figure 6f). Overall, this volume fraction value remains relatively small, reflecting the broad width of the correlation peak at *Q* ≈ 0.0065 Å^−1^. On the contrary, the volume fraction for FeNF-E in the *X* direction parallel to the field increases from 0.14 in the 7 mT field to 0.28 in 500 mT, consistent with chain-like alignment over large distances. Correspondingly, the width of the correlation peak at *Q* ≈ 0.010 Å^−1^ decreases substantially with increasing field (Figure 6a–c), but the peak position remains approximately constant with a fitted center-to-center distance (i.e., twice the hard-sphere radius in Table 2) between the MNFs of 56 nm on average. Comparing this length scale to the dimensions of the ellipsoids, we surmise (Figure 7b) that the nanoflowers are ordered linearly or in a zig-zag (i.e., branching) chain such that their spacing is almost twice the ellipsoid’s full width (i.e., 31 nm on average). (This interpretation is grounded in the expected physical behavior.^[46]^) It is notable that the fitted value of the volume fraction for FeNF-E along the *Y*-axis is effectively zero, indicating these nanoflowers do not exhibit long-range correlations perpendicular to the field (i.e., the loose chains are well separated). Overall, we conclude that at high fields, FeNF-E forms close-packed, hexagonal chains parallel to the applied field while retaining local 3D trigonal clusters. These thick chains are well-separated in the direction perpendicular to the field (Figure 7b). Conversely, the dipoles formed in FeNF-D reorient to a lesser degree with their long axis preferentially parallel to the field.

**Figure 8.**
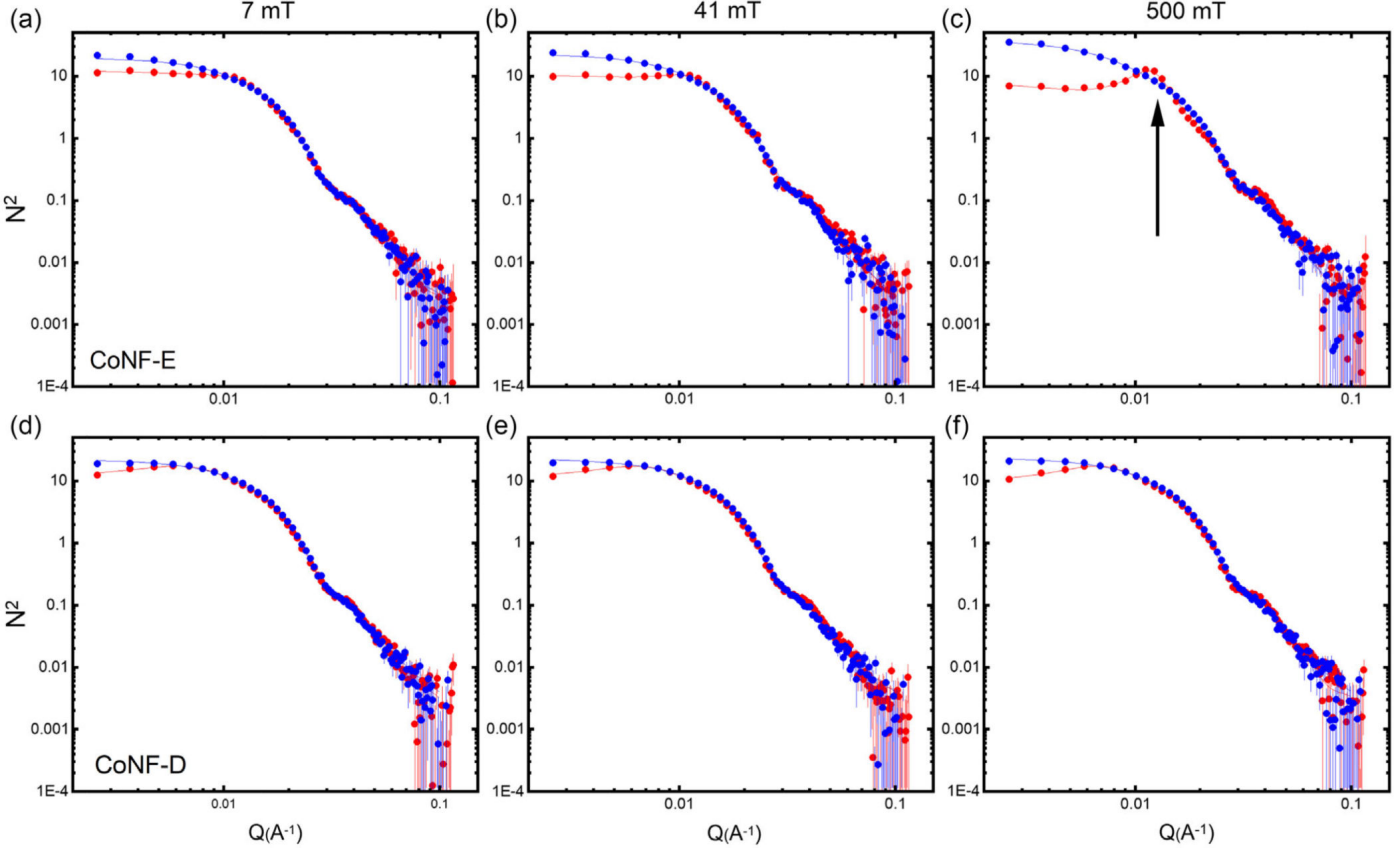
Structural SANS measurements ($I^{↑}+I^{↓}$ from half-polarized scattering) and corresponding fits at room temperature for (top row, a-c) CoNF-E and (bottom row, d-f) CoNF-D in magnetic fields of (left column, a and d) 7 mT, (center column, b and e) 41 mT, and (right column, c and f) 500 mT. Red, blue points are the structurally dominated scattering as seen along directions parallel and perpendicular (i.e., along the *X*- and *Y*-axis), respectively, to the magnetic field which is in the *X* direction. The lines are fits to the data using the model described in the Experimental Methods section. The arrow marks a correlation peak that is a signature of long-range order. Error bars are shown and represent 1σ, but may be smaller than the symbol.

Similar changes of the physical colloidal structure of CoNF-E and CoNF-D nanoflowers with magnetic field are evidenced by a similar approach to analyze data obtained from comparable SANS measurements, which are shown in Figure 8. The resulting fit parameters for the CoNFs are provided in Table 3. The equator radius parallel and perpendicular to the field remain constant at ≈13 nm, which again corresponds to the nanoflowers’ diameters as observed in TEM (Figure 4f,h). As for the FeNFs, the polar radii are consistently larger than the equator radii. Specifically, the ellipsoid length of CoNF-D is approximately twice (field average of 27 and 23 nm along the *Y*- and *X*-axes, respectively) its width at all fields, again suggesting two-particle dipole formation (Figure 7b). In comparison, the polar radius for the CoNF-E nanoflowers increases with field from two to three times the equator radius along the Y-axis and from three to more than five times the equator radius along the magnetic field direction (*X*-axis). Analogous to the FeNF system, the uncoated CoNF-E nanoflowers form larger (possibly cubic or diamond shaped) structures than their coated (CoNF-D) counterparts (Figure 7b). With respect to chain formation, the fitted values of the volume fraction and the center-to-center distance between nanoflowers (which is equivalent to twice the hard-sphere radius in Table 3) for the CoNF-D system follow the same trends as for the FeNF-D nanoflowers along the field direction (*X*-axis), indicating a loose, angled distribution of the dipoles (Figure 7b). However, the arrangement of these disjointed chains perpendicular to the field (*Y*-axis) is tighter than for FeNF-D with an average center-to-center distance corresponding to (or slightly less than) the diameter of a single nanoflower (≈22 nm). The situation for CoNF-E is comparable to that of FeNF-E with chain-like alignment along the *X*-axis (field direction) that lengthens with increasing field. The center-to-center distance of 48 nm between CoFe-E nanoflowers is roughly constant with magnetic field and is again approximately equal to twice the equator diameter. As with FeNF-E, CoNF-E appears to arrange in locally clustered, kinked chains that are well-separated perpendicular to the field (Figure 7b), as evidenced by the zero value of the volume fraction along the *Y*-axis at all fields. Overall, the evolution of the colloidal structure of the CoNF-E and CoNF-D nanoflowers with magnetic field follows a pattern observed from the FeNFs, though the chains and clusters generated by the CoNFs tend to be larger.

**Figure 9.**
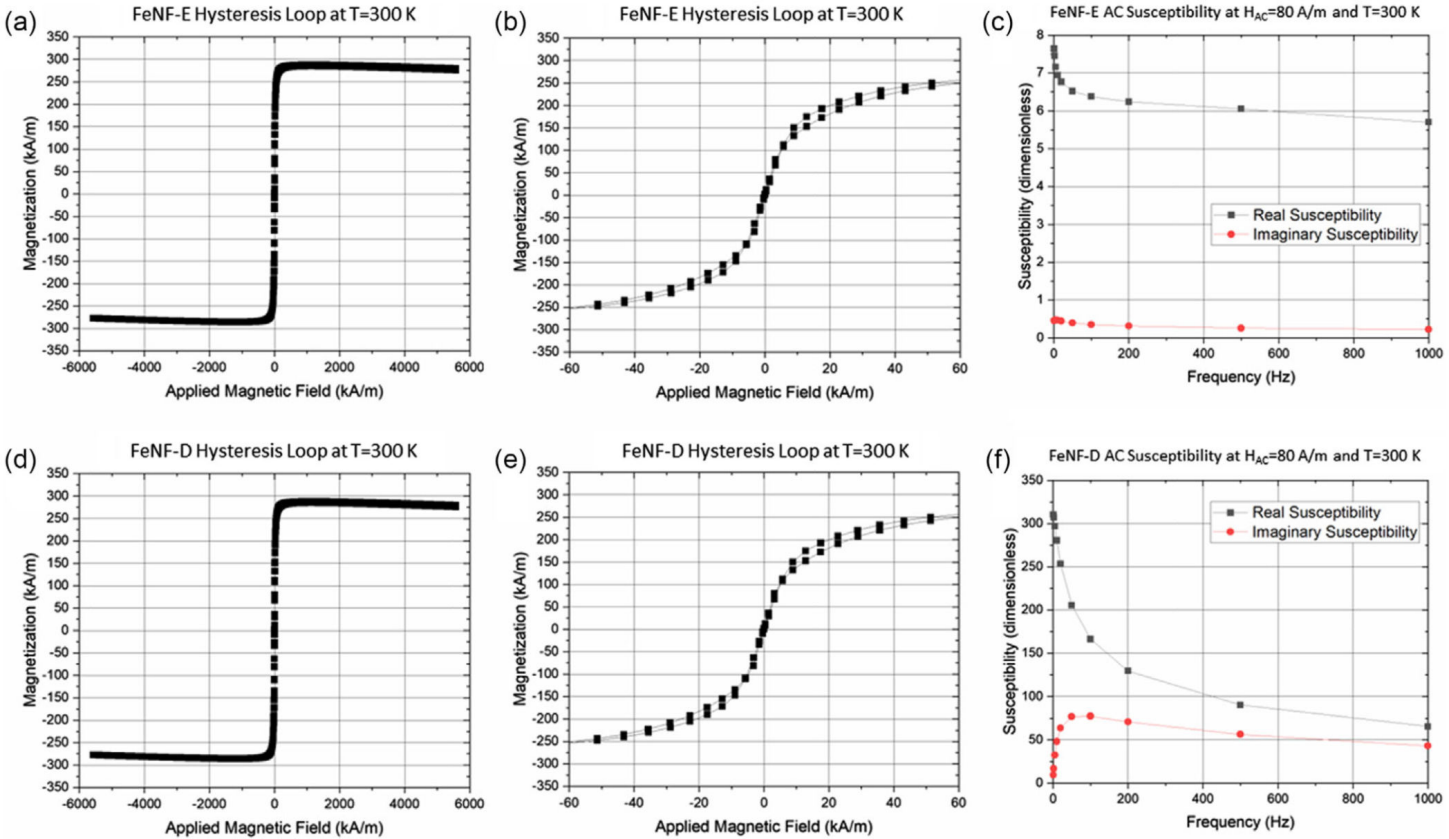
Magnetization as a function of field at room temperature for a) FeNF-E and d) FeNF-D samples with blow ups of the low-field region in b,e), respectively. The real and imaginary parts of the AC susceptibility are plotted as a function of frequency in c) for FeNF-E and in f) for FeNF-D. (Temperature-dependent ACS data for FeNF-D are shown in Figure S2, Supporting Information.) Error bars are shown and represent 1σ, but may be smaller than the symbol.

**Figure 10.**
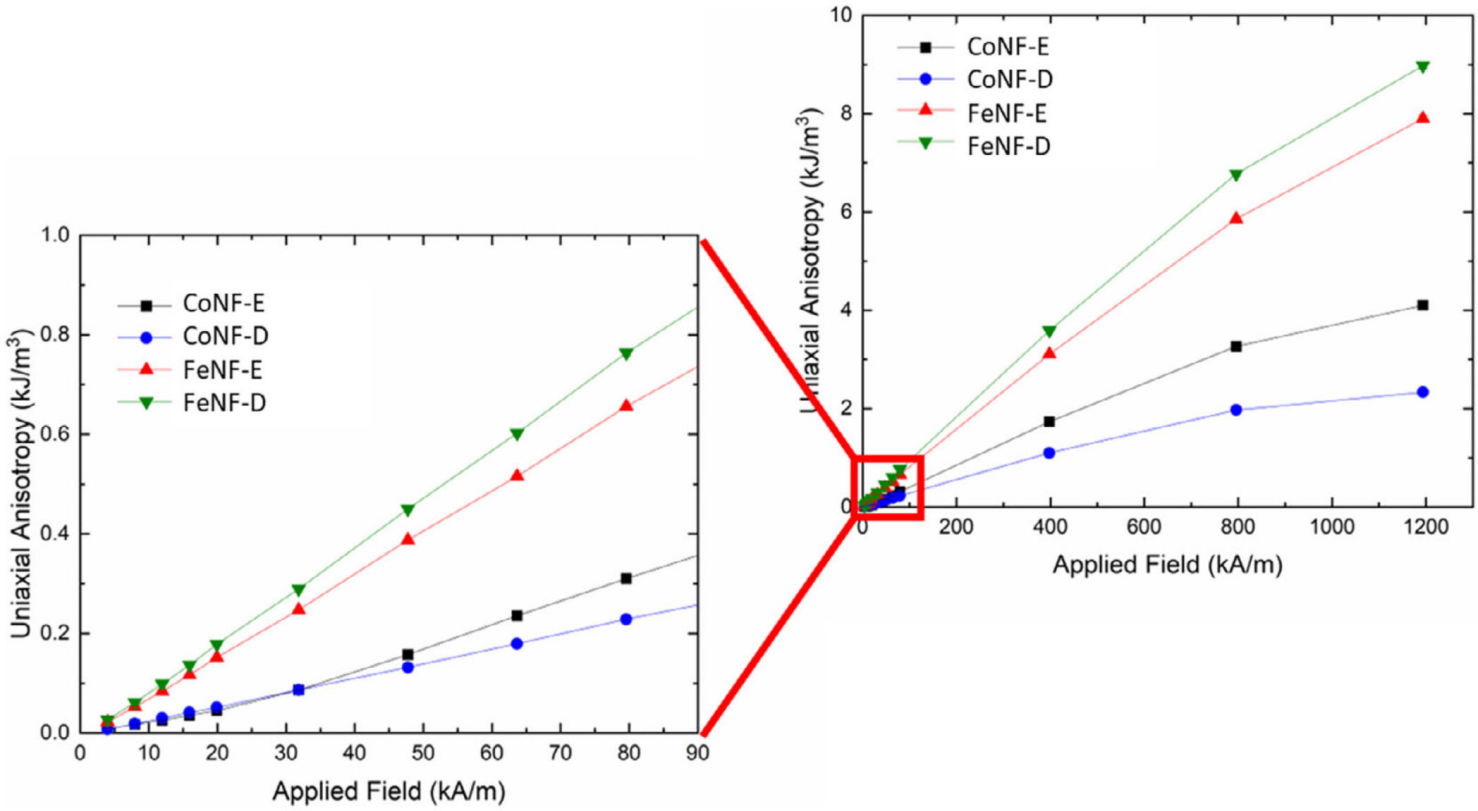
Effective magnetic anisotropy measured from torque magnetometry as a function of applied magnetic field. Only the dominant contribution (uniaxial, *K*_2_) has been plotted for simplicity. Values for the magnetic anisotropy for all contributing components are provided in Table 4. Normalization is by total volume of magnetic material estimated to be present in the sample (Table 1). Error bars are shown and represent 1*σ*, but may be smaller than the symbol.

**Figure 11.**
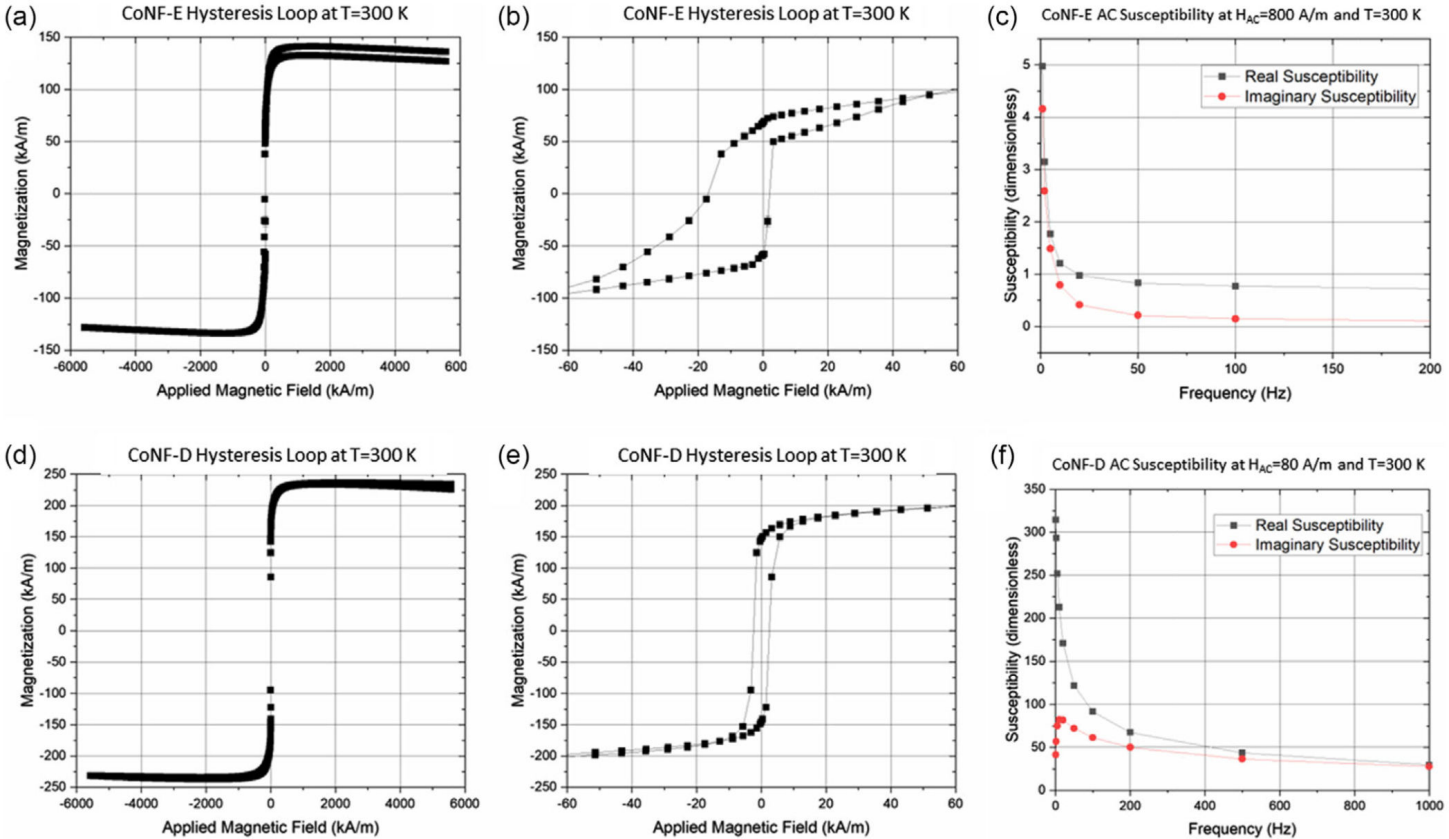
Magnetization as a function of field at room temperature for a) CoNF-E and d) CoNF-D samples with blow ups of the low-field region in b,e), respectively. The real and imaginary parts of the AC susceptibility are plotted as a function of frequency in c) for CoNF-E and in f) for CoNF-D. (Temperature- dependent ACS data for CoNF-D are shown in Figure S3, Supporting Information.) The disparity between the measurements in CoNF-E at high fields and in the coercivity of the ascending and descending curves is due to large scale chaining at those fields, which causes artifacts in the measurement. Error bars are shown and represent 1σ, but may be smaller than the symbol. (See Figure 15 for explanation of difference).

SANS measurements reveal that the colloidal structures of the FeNFs and CoNFs are comparable, though both systems exhibit a strong dependence on the type of surface coating (Figure 7b). These structural differences manifest in their magnetic responses in an applied magnetic field (Figures 9–11). The DC *M* vs. *H* curves are similar for the coated and uncoated FeNF nanoflowers, with square-like hysteresis loops that saturate at fields less than 40 kA m^−1^. FeNF-E has no measurable hysteresis (Figure 9a,b), while FeNF-D shows a coercivity (Figure 9d,e) of 2.88 kA m^−1^ (36 Oe). The square-like curve for both is characteristic of a ferromagnet, with FeNF-D being slightly “harder”. More significant differences become evident in the measurements of the zero-field ACS, shown in Figure 9c,f. Specifically, the imaginary susceptibility as a function of frequency for FeNF-E only shows the expected increase as the frequency decreases toward 1 Hz, whereas the susceptibility measured from FeNF-D has a pronounced peak near 80 Hz.

This peak in the ACS typically originates from one of three sources: domain wall motion, Brownian rotation, or Néel rotation (see the Experimental Methods section for additional information). To determine the origin of this peak and the information it can impart about the MNFs, each possibility is considered individually. Since the samples came from the same batch and the core is nominally the same in both the coated and uncoated FeNFs, their individual reversal mechanism should be identical. Therefore, if a reversal domain forms and grows within the nanoflower (the well-known process of domain nucleation and growth) in one MNF (i.e., domain wall motion), it should also form and move through the other. However, the peak in the ACS only occurs in FeNF-D, and so domain wall motion can be eliminated as the origin. Brownian rotation of the MNFs has a characteristic frequency (Equation (1) in the Experimental Methods section) that depends upon the size of the rotating object (and viscosity of the medium, at a defined temperature). Judging by TEM, XRD, and SANS, this base magnetic unit could be a magnetic grain, an individual nanoflower, or a cluster of nanoflowers. The peak frequency calculated from Equation (1) is well outside the range measured for both FeNFs regardless of the base magnetic structure used for the estimate (1.8 MHz, 180, or 4.0 kHz, respectively). By process of elimination, Néel rotation (via coherent rotation) of the magnetization within the MNFs must be the origin, and we can estimate the effective magnetic anisotropy (*K*_eff_ ) from Equation (2) in the Experimental Methods section. As with Brownian rotation, the base magnetic unit could be a magnetic grain, a MNF, or a nanoflower cluster. For FeNF-D, K_eff_ is either 60 kJ m^−3^ for a grain, 6.0 kJ m^−3^ for a nanoflower, or 0.38 kJ m^−3^ for a two-nanoflower dipole cluster. (Refer to dimensions obtained from SANS in Table 2). For an individual nanoflower, this number is within a factor of two of previous measurements^[45]^ for iron oxide, but the effective anisotropy is a factor of 5 too large for a grain and more than a factor of 30 too small for the dipole cluster. Therefore, the anisotropy is most likely associated with an individual nanoflower.

Since no peak is evident in the ACS of FeNF-E, we hypothesize the base unit generating the effective anisotropy to be different than that of FeNF-D. Assuming an anisotropy of 6.0 kJ m^−3^ for a nanoflower, we calculate the characteristic frequency of Néel rotation using Equation (2) in the Experimental Methods section from the large nanoflower clusters in FeNF-E (Table 2) and find that it would shift the peak completely out of the frequency range measured (≪ 1 Hz), as is observed. However, this approximation may be an over-simplification, as it is known that there are at least two competing effects occurring in the cluster: 1) a reduction in the magnetic anisotropy due to easier magnetic reversal of a MNF through collective behavior among neighboring MNFs and 2) an increase in the magnetic anisotropy due to structural shape effects from physical colloidal structures^[47]^ that impedes the easy rotation in low-AC magnetic fields. To probe further the origin of the effective anisotropy, torque magnetometry measurements were made on the MNFs as a function of field from which the unidirectional, uniaxial, trigonal, and cubic anisotropy constants were extracted using Equation (3) in the Experimental Methods section. The uniaxial anisotropy constant (*K*_2_) is plotted as a function of field in Figure 10, and all the components of the magnetic anisotropy measured under saturating magnetic field conditions are provided in Table 4. The dominant component of the magnetic anisotropy measured is uniaxial (*K*_2_) and is 7.90 kJ m^−3^ for FeNF-E and 8.98 kJ m^−3^ for FeNF-D. We conclude that the competition described above results in comparable values of the effective magnetic anisotropy for both FeNFs, despite the differences in their colloidal structures.^[47]^ Since only FeNF-D shows a peak in the ACS, we conclude that the ACS-measured effective magnetic anisotropy is associated with individual nanoflowers in FeNF-D and instead with larger trigonal clusters in FeNF-E (Figure 7b). This difference in the magnetic base unit is due to the strength of the interactions between the MNFs, which is controlled predominantly by the coating.

Magnetic hysteresis loops measured from the CoNFs show similar trends (Figure 11a,b,d,e), with a more S-shaped curve with very small coercivities for both the coated and uncoated samples. In addition, ACS measurements (Figure 11c,f) show a peak for CoNF-D around 20–30 Hz, but no peak for CoNF-E. As before, domain wall and Brownian motion are ignored as significant contributors to the measured peak for CoNF-D, and the effective magnetic anisotropy is estimated (Equation (2) in the Experimental Methods section)^[48]^ to be either 138 kJ m^−3^ for a grain, 8.9 kJ m^−3^ for a nanoflower, or 1.1 kJ m^−3^ for a two-nanoflower dipole cluster (Refer to dimensions obtained from SANS in Table 3). For all of these, values are significantly less than the theoretical value of bulk cobalt ferrite of 353 kJ m^−3^, which makes it difficult to determine the magnetic base unit here.^[49]^ However, it is known that the arrangement of iron and cobalt atoms and the exact oxidation state play a large role in determining the magnetocrystalline anisotropy, and so the reduced values are not unexpected.^[47]^ To help identify the magnetic base unit, torque magnetometry measurements were again performed as a function of field, with results plotted in Figure 10 and listed in Table 4. Though significantly smaller than bulk, the dominant magnetic anisotropy component at saturation was uniaxial $\left(K_{2}=4.1\text{kJ}{\text{m}}^{-3}\right)$ for CoNF-E and uniaxial $\left(K_{2}=2.3\text{kJ}{\text{m}}^{-3}\right)$ plus trigonal $\left(K_{3}=2.3\text{kJ}{\text{m}}^{-3}\right)$ for CoNF-D. The latter most closely matches that of the cluster K_eff_ from the ACS for CoNF-D. This indicates that the magnetic base unit is the cluster for both the coated and uncoated CoNFs. Note that when this value is applied to the quad-pole structure of CoNF-E, the ACS peak is shifted completely out of the frequency range measured (≪ 1 Hz), as is observed.

**Figure 12.**
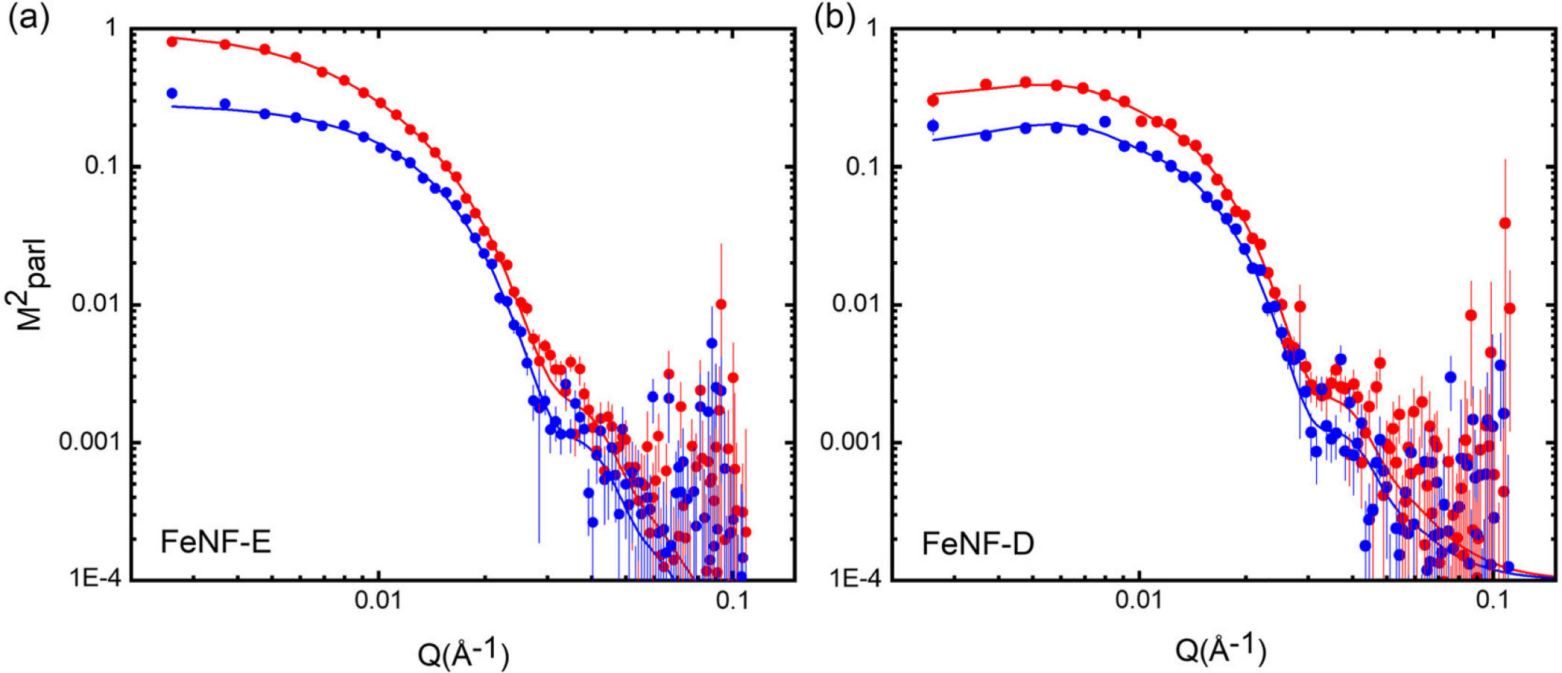
Square of the net magnetization parallel to the applied magnetic field (along the *X* direction) obtained from half-polarized SANS measurements and corresponding fits at room temperature for a) FeNF-E and b) FeNF-D. These data were obtained from sector averages along the *Y* direction, where the selection rules are satisfied that the scattering vector *Q* is perpendicular to the net magnetization direction. (See the Experimental Methods section for more information.) Red and blue data points were obtained at 500 mT and 7 mT, respectively. (For clarity the intermediate 41 mT data are not shown.) *M*^2^_parl_ is in arbitrary units. The lines are fits to the data using the model described in the Experimental Methods section. Error bars are shown and represent 1*σ*, but may be smaller than the symbol.

To correlate the observed magnetic behavior with the local colloid structure, polarized SANS measurements were performed to isolate the magnetic scattering resulting from the inter- and intra-particle magnetic order. Figure 12 shows plots of the square of the net magnetization parallel to the field $\left(M_{\text{parl}}^{2}\right)$ versus Q for FeNF-E at 7 mT and 500 mT (left plot) and for FeNF-D at the same fields (right plot). These data were obtained from the $I^{↑}$ and $I^{↓}$ cross sections (Figure S6, Supporting Information) using Equation (4) (see the Experimental Methods section). (Note that $M_{\text{parl}}^{2}$ corresponds to the net magnetization parallel to the field along the *X*-axis, but the Q dependence of $M_{\text{parl}}^{2}$ along the *Y*-axis reflects only correlations perpendicular to the field direction.) While the shape of these curves for both FeNFs is similar to their structural equivalents (blue curves) shown in Figure 6, the values of some of the parameters obtained from the fits differ (Table 2 relative to Table 5). In particular, while the fitted values of the magnetic equator radii for both FeNFs are similar to their corresponding structural radii, the fitted values of the magnetic polar radii are on average 4 nm smaller than their structural counterparts. This means that the clusters of 2–3 MNFs that form perpendicular to the applied field are only partially magnetized with a magnetic coherence that is less than the extent of the structural ordering. Some of this difference could be attributed to spin canting or oxidation at the nanoflower surface.

**Figure 13.**
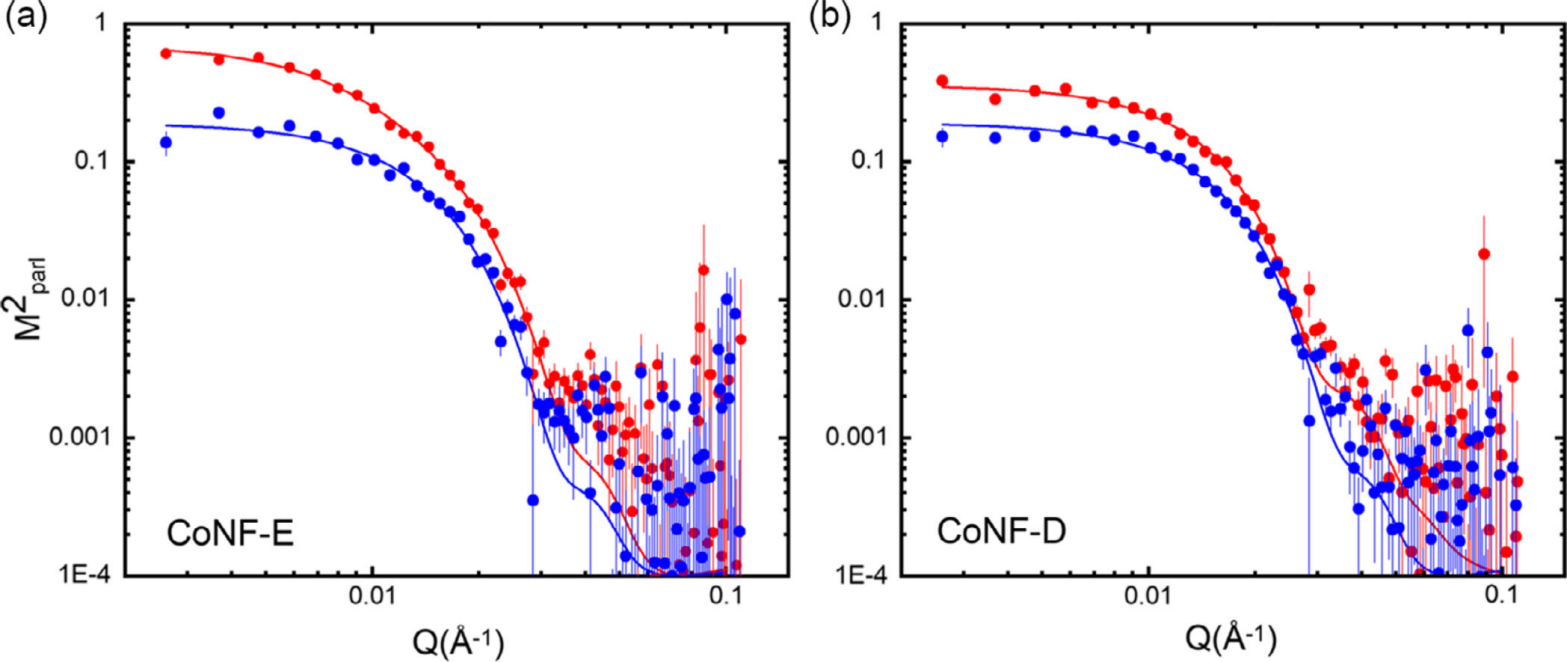
Square of the net magnetization parallel to the applied field (along the *X* direction) obtained from half-polarized SANS measurements and corresponding fits at room temperature for a) CoNF-E and b) CoNF-D nanoflowers. These data were obtained from sector averages along the *Y* direction, where the selection rules are satisfied that the scattering vector *Q* is perpendicular to the net magnetization direction. (See the Experimental Methods section for more information.) Red and blue data points were obtained at 500 and 7 mT, respectively. (For clarity, the intermediate 41 mT data are not shown.) The best fits (lines) were obtained for a core/shell ellipsoid with reduced magnetization in the shell. For the hard-sphere structure factor multiplying the ellipse, the hard-sphere radii and volume fractions for CoNF-D were fixed to the values obtained from the corresponding structural fits (Table 3), and the volume fraction for CoNF-E was set to zero consistent with the structural fits. *M*^2^_parl_ is in arbitrary units. Error bars are shown and represent 1*σ*, but may be smaller than the symbol.

Figure 13 shows equivalent $M_{\text{parl}}^{2}$ data for the CoNF samples. Fitting models to the data reveal that differences in the structural and magnetic coherence lengths also appear in the CoNFs and are slightly more pronounced for CoNF-D. In that case, the structural coherence length (Table 3) is 3–5 nm larger than the value of the total magnetic polar radius (Table 6) along the *Y* perpendicular direction for all fields measured. (Note that the structural equator radii are consistently 1–2 nm smaller than the magnetic polar radii, possibly indicating preferential alignment of the magnetization only in tilted ellipses or just reflecting the limitations of the ellipse parameterization). The origin of this difference in both the coated and uncoated CoNFs is complicated by the existence of a 3–5 nm thick magnetic shell with reduced magnetization (Table 6), which corresponds to the outer portion of the nanocrystal grains surrounding the nanoflower core. Unfortunately, we have insufficient information to identify the cause of this shell, as it could be many things. Since the magnetization of the ferrites occurs due to super-exchange through the oxygen, very small changes in lattice dimensions, bond angles, occupancy, etc., can result in significant changes in the magnetization. These effects could be caused by stress or strain differences in the lattice at the surface versus the core, changes in the (tetrahedral vs octahedral) site occupancy of the Co within the inverse spinel structure, non-uniform distribution of the Co throughout, changes in oxidation at the surface, variation in crystallographic grain boundary type, and others. Any of these issues could result in a local reduction in the net magnetization directly or could give rise to local competing anisotropies in the grains surrounding the nanoflower core that lead to to magnetic frustration at the MNF surface. We do know that significant changes in composition cannot be the origin of the shell, however, as the unpolarized SANS data (Figure 8) are best fit with an ellipsoid with a uniform scattering length density (SLD) (i.e., no shell).

From fits to these data, we can determine the magnitudes of the magnetization in each of the samples (see Table 5 and 6). The values measured at 500 mT (near saturation) for FeNF-E (340 kA m^−1^) and FeNF-D (334 kA m^−1^) are similar and lower than, but comparable, to that of bulk maghemite (360 kA m^−1^) and magnetite (476 kA m^−1^), as is expected.^[50]^ In the 7 mT field, the magnetization for both FeNFs is reduced to ≈80% of values measured at 500 mT. The saturation values from SANS are in rough agreement with those determined by *M* vs. *H* measurements (287 and 338 kA m^−1^, respectively, for FeNF-E and FeNF-D from Table 4). When comparing the bulk magnetization and SANS values, it is important to note that the latter measures the absolute value of the total magnetic moment since the magnetic scattering was normalized to the structural scattering (Refer to Experimental Techniques). In contrast, the former includes contributions from the water and surfactant, which is then normalized by the mass of the nanoparticle cores. The result includes the mass of unmagnetized sample components, such as the loose grains. As a result, the SANS estimates of the saturation magnetization tend to be higher than the estimates from *M* vs. *H*.

The values measured at 500 mT (near saturation) of the magnetizations of the cores are 436 kA m^−1^ for CoNF-E and 364 kA m^−1^ for CoNF-D, and the magnetizations of the shells in both CoNFs are reduced by more than a factor of two. The values for the core saturation magnetization are smaller than the CoFe_2_O_4_ bulk magnetization, which is 498 kA m^−1^. By weighting the magnetic contributions from the core and shell by volume, we obtain estimates of the reduced average magnetization of each MNP, which is 285 kA m^−1^ for CoNF-E and 274 kA m^−1^ for CoNF-D. These are substantially reduced from bulk but are both higher than the values determined by *M* vs. *H* (141 and 236 kA m^−1^, respectively, for CoNF-E and CoNF-D from Table 4), which measures the average value of the core and shell, normalized by volume. (Note that the differences between the SANS and *M* vs. *H* magnetization values again are related to different normalization approaches, as discussed earlier.) The suppression of the average magnetization in the CoNFs is clearly related to the previous result of the significantly reduced effective magnetic anisotropy in the CoNFs, as compared to bulk CoFe_2_O_4_.

**Figure 14.**
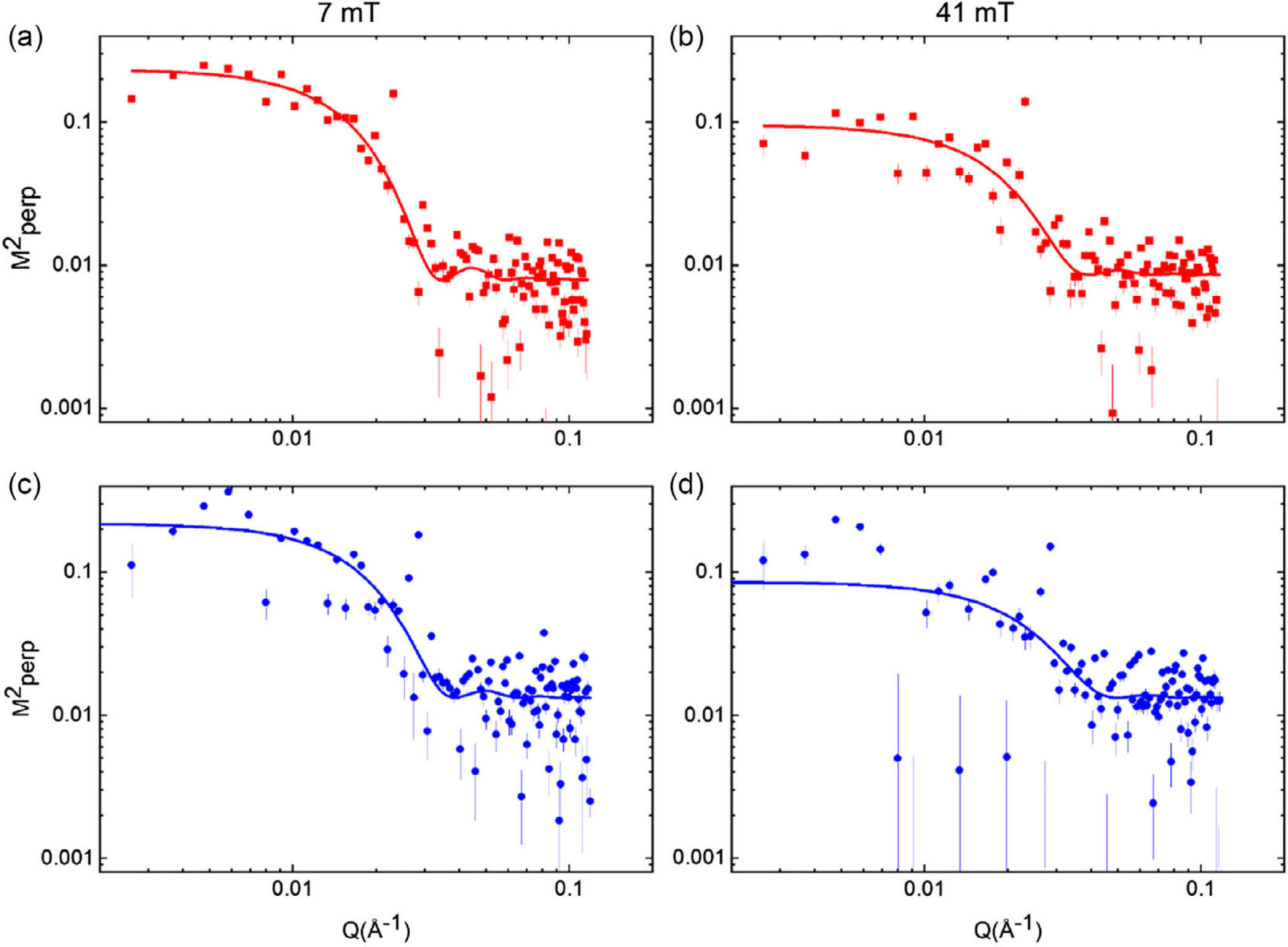
Square of the net magnetization perpendicular to the applied field (along the *X* direction) obtained from full-polarized SANS measurements and corresponding fits to a spherical function at room temperature for FeNF-E in fields of (left column) 7 mT and (right column) 41 mT. The red curves in a,b) correspond to scattering as seen along the *X* direction (Equation (6)), and the blue curves in c,d) correspond to scattering along the *Y* direction (Equation (5)). Error bars are shown and represent *1σ*, but may be smaller than the symbol.

Additional details pertaining to magnetic domain sizes can be gleaned from full polarization SANS measurements which are sensitive to the magnetization both parallel and perpendicular to the magnetic field. Due to neutron beamtime limitations, we investigated only FeNF-E and CoNF-D in this scattering configuration, and we focus here on the field-dependent spin flip data (Figure 14), which are sensitive to the magnetization components perpendicular to the magnetic field (see Experimental Methods for more information). Figure 14 separately shows the *Q* dependence of *M*_perp_ along the *X* and *Y* directions (top and bottom row, respectively) in fields of 7 and 41 mT (right and left column, respectively) for FeNF-E in D_2_O. The overall shapes of these curves are all similar with only a systematic decrease in intensity with increasing field up to 500 mT (data not shown) as the spins align parallel to the field. The radii extracted from simple sphere fits to these curves ranged from 9.1 to 13.0 nm and were comparable to the structural equator radii for FeNF-E reported in Table 2, which range from 13.3 to 16.5 nm. This result indicates that each magnetic domain extends beyond an individual nanocrystal grain in the direction perpendicular to the field but is confined to a single nanoflower. In addition, there is a subtle difference in these magnetic length scales along the *X* and *Y* directions. For example, in 7 mT, the sphere radius along the *X*-axis parallel to the field was determined to be 13.0 nm (Figure 14a) in comparison to 11.7 nm along the *Y*-axis perpendicular to the field (Figure 14c). The fitted sphere radius in a 41 mT field was similarly larger along the *X*-axis in comparison to the *Y*-axis (11.4 and 9.1 nm, respectively, from Figure 14b,d). This trend is consistent with the increase in the structural equator radius along the field direction (Table 2) that was attributed to the tilting of the MNFs. For comparison, the spherical fits to comparable spin flip data for CoNF-D in D_2_O in a 7 mT field (Refer to Figure S7, Supporting Information) give radii values of ≈12 nm both along the *X*- and *Y*-axes. Though these particles are coated with dextran, the results again confirm that the transverse domains extend beyond a single grain but not beyond a single nanoflower. Coating, regardless of its nature, seems to have a little effect on magnetic domain size.

**Figure 15.**
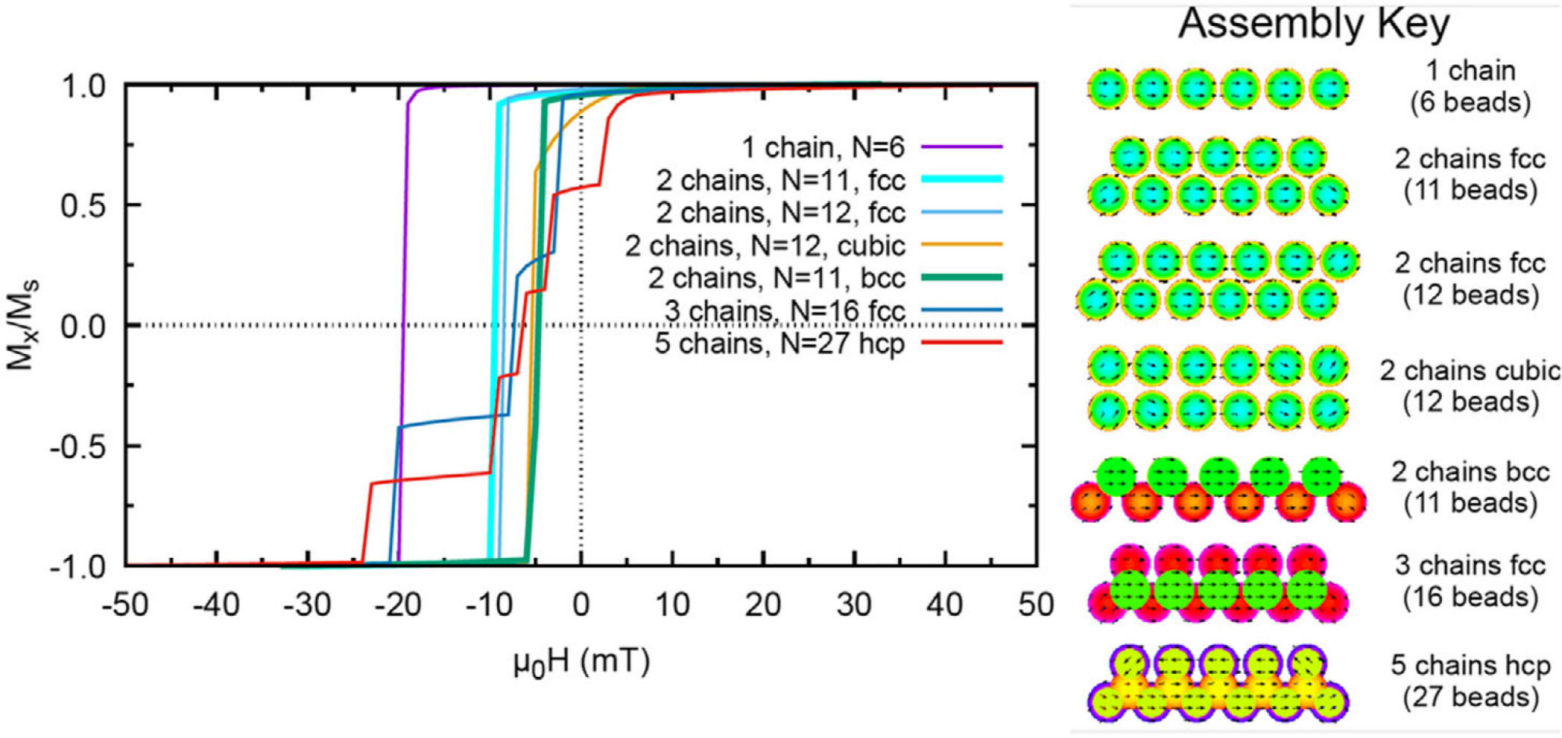
Object oriented micromagnetic framework (OOMMF) modeling of equilibrium states as a function of applied magnetic field for different structural configurations. Models used cobalt ferrite parameters from Table 3 and 6, specifically M_s_ = 385 (core)/165 (shell) kA m^−1^, 17 nm core, d = 26 nm, A = 13.2/7.6 (interface) pJ m^−1^, K = 0, 1 nm spacing, which also corresponds to Figure SI-11(i). Only half of the hysteresis loop is shown. Each color represents a different chain configuration, but the same parameters.

Finally, we used object oriented micromagnetic framework (OOMMF) modeling^[51]^ to better understand the magnetic behavior associated with the different chaining and clustering behavior revealed by SANS for these nanoflower systems. In the model (details can be found in the Experimental Methods section), we focused on individual nanoflowers (at 0 K), and how they reversed (equilibrium states) as a function of their idealized fixed chain arrangement (Figures 15 and S11, Supporting Information). The general trends of the simulations qualitatively match those of the experiments as the calculated *M* vs. *H* is sharp with higher coercivity for thin, loosely packed chains, but the calculated M vs. H is rounded with steps for thick, tightly packed chains. The steps are likely due to a partial reversal of the ensemble of MNFs: when one nanoflower, presumably near the end of the chain, flips its magnetization direction, its neighbor then flips, followed by its neighbor, and so on, until the entire chain has reversed. The ease with which the reversal occurs depends on the strength of the dipolar interactions, which are strongest in the uncoated CoNF-E sample that is electrostatically stabilized, and the only sample where the steps are clearly observed (Figure 11b, descending curve).^[50]^ In confirmation with the SANS results, FeNF-E tend with increasing field toward a two-chain cubic transitioning to a three-chain fcc/five-chain hcp structure (Figure 7b) which is softer and has a more S-shaped *M* vs. *H* curve (Figure 9a,b); in contrast, FeNF-D and CoNF-D both tend toward a one-chain structure (Figure 7b) having squarer loops (Figure 9d,e and 11d,e, respectively). CoNF-E (Figure 11b) resembles the two-chain bcc structure at low fields and a three-chain fcc/five-chain hcp structure at high fields.

## 3. Discussion

Our objective here is to ascertain the relative influence of composition, magnetic anisotropy, and interparticle interactions on MPI and MNPH performance found in MNFs. To achieve our objective, we selected two different MNF compositions exhibiting different magnetic anisotropies, with each being either electrostatically stabilized or sterically stabilized (with dextran). The FeNFs display higher SLP and PSF values than did the CoNFs. These remain unchanged with coating for the FeNFs. By contrast, SLP and PSF of CoNFs substantially decrease with the addition of a dextran coating.

All four samples show distinctly different colloidal structures when exposed to an external magnetic field, with the greatest contrast appearing between the branched and clustered chains that formed in the electrostatically and sterically stabilized MNFs, respectively (Figure 7b). (Recall that the same nanoflower cores are from the same batch.) These differences, however, are not reflected in the performance of the MNFs in MNPH and MPI. In addition, the difference in the saturation magnetization of the FeNF and CoNF uncoated samples (Figures 9 and 11) does not scale with the magnitude of the differences in the SLP or the PSF, even when the CoNF magnetization is averaged over the core and shell (Table 6). Though the contrast of measured average magnetization between coated FeNF and CoNF samples is also large, it does not account for the significantly lower SLP and PSF amplitude measured from the latter. Therefore, the magnetization alone cannot account for the observed performance trends. Instead, the primary differences in the heating and imaging behaviors must be associated with the effective magnetic anisotropies of each MNF. Results shown in Figure 10 and Table 4 demonstrate that CoNF-D displays an uniaxial anisotropy four times lower than that measured from the FeNFs, and two times less than measured from CoNF-E, even in the AMF peak amplitude ranges used for the SLP measurements.

As the SLP (Figure 1) depends on the AMF amplitude and frequency, as well as on the magnetic moment and magnetic anisotropy of the MNF, CoNF-E with its low effective anisotropy (Table 4) produces significantly less measured heat than the FeNFs, and also CoNF-D, having the lowest anisotropy among the samples measured, demonstrates the lowest heating efficiency.^[23,47]^ In comparison, the FeNF-D and FeNF-E particles have a large enough effective magnetic anisotropy, originating from the individual nanoflowers and nanoflower clusters, respectively, to produce significant heat, but not so large as to require large peak amplitude AMFs to generate enough energy to overcome the reversal energy barrier. In general, an SLP of at least 100 W/gFe is needed to heat tumors (>1 cm^3^) for MNPH applications. As such, all the particles, except CoNF-D demonstrate clinically relevant heating at the chosen AMF test conditions.

As MPI is based on the non-linear response of the MNFs to an AC magnetic field, the AC response becomes linear, leading to little signal if the magnetic anisotropy is too large or too small. The magnetic anisotropy of the FeNF nanoflowers is likely inside the “Goldilocks zone”, resulting in a good non-linear response, while CoNF-E likely has too small of a magnetic anisotropy, resulting in a linear response. CoNF-D is even smaller, giving rise to an even weaker linear response. In fact, the observed differences in PSF amplitude (Figure 2) correspond with the factor of two differences between the magnetic anisotropy of the CoNFs. This result indicates that the as-synthesized FeNFs are more promising for MPI than the as-synthesized CoNFs.

From our analysis of data, we conclude that the MNF coating alters the size and shape of the colloidal structure formed in AC and DC magnetic fields for all samples tested. Specifically, we propose a model to explain the behavior of both coated MNFs (FeNF-D and CoNF-D) in the presence of a magnetic field in which the individual nanoflowers form two-particle clusters as a base structure, likely from the influence of dipole interactions occurring during synthesis. These dipoles then form loose, partial chains at low fields that coalesce into larger branched chains at high fields. Our analysis of SANS data shows the chains are well separated in the direction perpendicular to the field, though the spacing between them decreases as the field is increased. The primary difference between FeNF-D and CoNF-D that we could discern is that the latter shows evidence of chains with tighter packing perpendicular to the field, possibly corresponding to collapsed branches (Figure 7b). In contrast, both electrostatically stabilized MNFs (FeNF-E and CoNF-E) form larger clusters in low fields, which appear to have a trigonal structure for FeNF-E and a cubic or diamond structure for CoNF-E (Figure 7b). This behavior is unsurprising since the particles are stabilized primarily by electrostatic forces, thus enabling closer packing than would be possible with steric stabilization. (Note that the discrepancy in the hydrodynamic diameter (44–45 nm) and TEM core diameter (26–30 nm) for FeNF-E and CoNF-E can be explained by the trigonal/cubic character of the FeNF/CoNF clusters, which likely form during synthesis.) We surmise that this trigonal cluster is also likely a flux closure structure, reducing the measured magnetization at low fields to close to zero (as is observed). In contrast, cubic/diamond clusters in CoNF-E may be a flux closure structure, but this may not always be the case as its formation may depend upon field history. If true, this would manifest a zero-field (remnant) magnetization in the *M* vs. *H* loop of CoNF-E being significantly larger than in FeNF-E, which is observed. In large fields (500 mT), we conclude from our analysis that these clusters appear to compress to form long, thick, zig-zag chains with a packing having hexagonal structure for FeNF-E. It is notable that the chains formed in CoNF-E appear to be dense and elongated parallel to the field direction. These chains are well-separated perpendicular to the field for both electrostatically MNFs.

The measured difference in the colloidal structures of the electrostatically and sterically stabilized MNFs has implications for the AC and DC magnetic response. DC magnetic hysteresis loops for the sterically stabilized samples show a square shape with low coercivity, whereas the hysteresis loops measured from the electrostatically stabilized samples appear to be more rounded (with steps present for CoNF-E). OOMMF modeling finds that this behavior is consistent with the type of chain formation that is occurring. Only the coated MNFs show a peak in the AC susceptibility, with an estimated magnetic anisotropy (see Table 6) associated with Néel rotation of the individual MNFs for FeNF-D and with MNF clusters for CoNF-D. In contrast, no features are observed with the electrostatically stabilized MNFs within the measurement frequency range. We conclude the estimated magnetic ansiotropy would be associated with Néel rotation of the larger MNF clusters.

While the colloidal structure of the MNFs and their resulting magnetic response depend on the presence of a dextran coating, the heating and imaging behaviors are sensitive primarily to the nanoparticle composition (i.e., ferrite vs cobalt ferrite) and their corresponding magnetic anisotropies, which are substantially lower for the CoNFs. The key difference between the CoNFs and FeNFs that explains the contrasting magnetic anisotropies is the nature of the magnetic order within each individual nanoflower. Specifically, we conclude from our analysis of polarized SANS data that the transverse magnetization is confined to a single nanoflower for both the CoNF and FeNF at all fields considered. However, the analysis also reveals that the CoNFs possess a thick disordered magnetic shell having a significantly reduced magnetization that is nearly unresponsive to fields up to 500 mT. For CoNF-D, the thickness of the shell appears to decrease slightly with increasing field. For both CoNF-D and CoNF-E in low fields, this shell constitutes more than 60% of the total volume of the cluster (Table 6). This magnetic shell formation could explain the reduced effective magnetic anisotropy seen in the CoNF samples as compared to the FeNFs. Together these effects suggest frustrated magnetization on the surface of each nanoflower that limits the extent of the magnetic coherence relative to the longer structural coherence. While the nanoflower construction is intended to reduce the overall MNF anisotropy by design, the actual effect in the case of CoNF seems to be the creation of a stiff magnetic outer shell around the nanoflower core with rigid, frustrated magnetization that is hard to rotate. Though the heating and imaging properties of both CoNF systems are poor relative to those of the FeNF, the performance of CoNF-E is better than that of CoNF-D, presumably because the closer packing of the uncoated MNFs facilitates longer-range magnetic interactions that partially overcome the magnetic frustration induced by the local competing anisotropies of the grains, thereby increasing the effective magnetic anisotropy. In general, these MNF structures, and their related effective magnetic anisotropy, clearly can have a profound impact on the AC magnetic response in applications, especially when the magnetocrystalline anisotropy is close to a performance limit for that application.

## 4. Summary and Conclusions

AC biomedical applications of MNFs abound, but knowledge gaps remain to relate structural and magnetic properties with optimal performance. We find that for MPI and MNPH, the magnetic anisotropy determines performance of the FeNFs and CoNFs measured. Though the colloidal structures (Figure 7b) formed by the electrostatically and sterically stabilized FeNFs differ, their heating and imaging properties are insensitive to coating, and are superior to those measured in the CoNFs. The effective magnetic anisotropy of the FeNFs is higher than that measured in the CoNFs and did not change with coating. We conclude that this is due in large part to the nanoflower structure of the core, creating more loosely coupled magnetic grains that can more easily align than can a single crystal. In contrast, the nanoflower structure for cobalt ferrite creates a reduced magnetization with a frustrated shell over a core that is decoupled from its neighbors. In this system, the magnetization of the outermost grains competes with the central core, generating intra-particle magnetic frustration that requires higher applied magnetic fields than were used in this study. In this manner, the CoNF structure appears to be opposite to that observed in the FeNFs. Though the colloidal structures formed by the CoNFs are of similar character to those that develop in the FeNFs, the magnetic interactions among the nanoparticles appear to be limited, which consequently limits the increase in the magnetic anisotropy with increasing magnetic field. Furthermore, the core–shell structure of the CoNFs produces an overall significant reduction of the magnetic anisotropy, which decreases both the heating and imaging performance. We conclude that the formation of denser-packed clusters and chains in the electrostatically stabilized CoNFs (CoNF-E) attenuate and compensate for the effects of the frustration. The intent of synthesizing MNF constructs is to deliberately create misaligned structural grains to reduce the overall magnetic anisotropy of the composite and to increase their suitability for heating and imaging applications. Our data show that this approach may be inappropriate for some nanoparticle compositions. The ramifications of the nanoparticle structural design on the effective magnetic anisotropy of the system must be prioritized since it clearly overshadows the influence of surface stabilization and even of colloid structure.

## 5. Experimental Methods Section

### Synthesis of Nanoflower MNFs:

The FeNF nanoflower in water samples examined herein (Table 1) were synthesized by micromod Partikeltechnologie, GmbH. The magnetite/maghemite samples were prepared by a modified polyol method,^[35,52]^ varying the stoichiometry of the iron(II) and iron(III) precursor salts as well as the temperature during synthesis. The starting nanocrystals clustered together to form the nanoflowers at the elevated temperatures. These uncoated nanoflowers comprised the first sample, FeNF-E, which were electrostatically stabilized through the precursor materials. The second sample, FeNF-D, was prepared by coating the FeNF-E nanoflowers post-synthesis with dextran (D) for steric stabilization. In short, these MNFs have essentially the same nanoflower core, but different surface stabilizations: electrostatic and steric. For most characterization measurements, the MNFs were suspended in H_2_O to form a water-based colloid with a MNF concentration of 16–20 mg mL^−1^. For the full-polarized SANS measurements, the original FeNF-E sample in H_2_O was dialyzed in 100% D_2_O, resulting in a solution that is ≈99% D_2_O/1% H_2_O.

Cobalt ferrite (CoFe_2_O_4_) nanoflower samples in water were also examined (Table 1). These were again synthesized by micromod Partikeltechnologie, GmbH, and were prepared by a modified polyol method,^[34,52]^ varying the stoichiometry of the iron(III) and cobalt(II) precursor salts as well as the temperature during synthesis. As in the FeNFs, the starting nanocrystals clustered together to form the nanoflowers at the elevated temperatures. These uncoated nanoflowers formed the first sample, CoNF-E, and were electrostatically stabilized through the precursor materials. The second sample, CoNF-D, was prepared by coating CoNF-E nanoflowers post-synthesis with dextran for steric stabilization. As in the FeNF, these MNFs have the same nanoflower core, but different surface stabilizations: electrostatic or steric (due to post-synthesis coating with dextran). Again, for most characterization measurements, the MNFs were suspended in H_2_O to form a water-based colloid with a MNF concentration of 16–20 mg mL^−1^. For the full-polarized SANS measurements, the original CoNF-D sample in H_2_O was dialyzed in 100% D_2_O, resulting in a solution that is ≈99% D_2_O/1% H_2_O.

### TEM:

Images were taken using the FEI Titan 300 kV analytical TEM with Gatan Oneview IS COMS camera. The samples were prepared by drop-casting the diluted (1/1,000) MNF suspension onto a carbon coated copper grid on filter paper. ISO 21363:2020 specifies the correct procedures for measuring size and shape distributions using transmission electron microscopy for nanomaterials. Our TEM measurements do not meet one of the fundamental requirements, which is a minimum number of particles, so we cannot quantitatively report the size and its distribution. This is why the data are presented as “approximately” and why no histogram and corresponding fitting function are shown. Instead, we use these approximate values as a consistency check with other measurements.

### XRD:

The crystal structure of the samples was determined from powder XRD on a D8 (Bruker AXS LLC, Madison, WI) diffractometer with Cu Kα radiation at room temperature. The diffractometer was equipped with Gobel mirror optics and a scintillation detector, and the sample was mounted in a “zero background” holder. XRD was performed on dried samples over the 2*θ* range of 20°–90° with a step width of 0.01° and a sampling time of 2 s. The approximate crystallite size was estimated using Scherrer analysis, which is most sensitive to the smallest dimension of the crystallite. The peaks were fitted with a Lorentzian function, and the full width at half maximum was used as the broadening value.

### DLS:

The hydrodynamic diameter of the particles was measured using DLS with a Panalytical Zetasizer (Malvern Panalytical Ltd., Malvern, UK) operating at a scattering angle of 90° and at a temperature of 25 °C. The samples in H_2_O were diluted at least 1/500 to reduce/eliminate effects of multiple scattering. The hydrodynamic diameter was calculated from the equivalent sphere model that best fits the data.

### Magnetometry:

Magnetization (*M*) vs. applied magnetic field (*H*) and *M* vs. temperature (*T*) measurements were made using a vibrating sample magnetometer with a superconducting quantum interference device (SQUID VSM [Quantum Design, Inc, San Diego CA, USA]). *M* vs. *H* measurements were performed at 300 K from ±5,570 kA m^−1^ (±70 kOe) while *M* vs. *T* measurements were conducted at temperatures ranging from 290 K to 350 K (to avoid freezing or boiling the water in the sample) in a static (or direct current, DC) magnetic field of 8 kA m^1^ (100 Oe). For both, the vibration amplitude was fixed at 4 mm, the range at 1000, and the measurement time at 4 s. 70 μL of MNFs suspended in H_2_O were loaded into a polychlorotrifluoroethylene liquid capsules (LakeShore Cryotronics, Westerville, OH) and sealed with epoxy to prevent water evaporation during measurements under vacuum (about 800 Pa). Backgrounds^[21]^ from the water and dextran as well as any residual magnetization resulting from flux trappage in the superconducting magnet (±2.8 kA m^−1^ [35 Oe]) were not subtracted. As a result, magnetic fields less than this flux trappage limit cannot be resolved and are considered not measurable. Data were normalized by total volume of magnetic material. As the diamagnetic background from the water and dextran were not removed, saturation magnetization values are expected to underestimate actual values. It is important to note that these samples were measured in liquid and the actual volume of MNFs can vary within that. Multiple samples were not prepared and measured to determine reproducibility, so actual errors due to variation in MNF quantity per unit volume may be larger than those reported.

### AC Susceptibility:

Alternating current susceptibility (ACS) measurements were performed using the SQUID VSM with the three-point measurement method at 300 K with “zero” DC magnetic field (24 A m^−1^ [0.3 Oe]); an AC magnetic field amplitude of 8 A m^−1^ (0.1 Oe), 80 A m^−1^ (1 Oe), or 800 A m^−1^ (10 Oe); and a frequency range of 1 Hz –1000 Hz. Five measurements were made under each AC field and frequency combination, with the measurement time fixed at 4 s per measurement and a range of 100. The smallest AC field was insufficient to generate measurable signal at all frequencies, and the other two AC magnetic fields used generated nearly identical data. For clarity, we only show the 80 A m^−1^ ACS data, except for CoNF-E where we show the 800 A m^−1^ data as the 80 A m^−1^ data had an error. The same liquid samples sealed in polychlorotrifluoroethylene liquid capsules were used for ACS, as well as the magnetometry measurements. Data were normalized by total volume of magnetic material.

Two main magnetic relaxation processes^[48]^ can occur on these time scales: Brownian (physical rotation of the entire MNF) and Néel (rotation of just the magnetic moment relative to the crystallographic axes) relaxation. Each of these has a characteristic time scale. The Brownian frequency $\text{(}\tau_{\text{B}}\text{)}$ for physical rotation of an object in a fluid is given by:

(1) $$\tau_{\text{B}}=\frac{3\eta V_{\text{H}}}{k_{\text{B}}T}$$

where η is the viscosity of the fluid, $V_{\text{H}}$ is the hydrodynamic diameter, and k_B_ is the Boltzmann constant. Calculations of the Brownian frequency assume the dynamic viscosity at 300 K for water. The Néel frequency $\left(\tau_{N}\right)$ for magnetic rotation is given by:

(2) $$\tau_{N}=\tau_{0}e^{\left(KV/k_{\text{B}}T\right)}$$

where $\tau_{0}$ is the attempt frequency (assumed to be 1 GHz), V is the volume of a discrete non-interacting magnetic unit, and the magnetic anisotropy energy density is *K*. (The magnetic anisotropy is the energy required to reverse the magnetization—the barrier height.) *K* can be estimated by assuming that the peak in the imaginary component of the ACS is the frequency of Néel rotation and that there are no interactions between the MNFs. Data are normalized by the volume of an “independent” magnetic unit, such as a single grain, a single nanoflower, or a single dipole cluster (two nanoflowers).

The aforementioned two magnetic relaxation processes assume reversal via coherent rotation of the magnetic moment—where the entire magnetic moment of the MNF rotates together. However, magnetic reversal is not necessarily coherent. Instead, reversal can occur by magnetic domain nucleation and growth. A magnetic domain is a region where all the atomic spins point in the same direction. In a MNF with a single magnetic domain, reversal starts by creating a second, small region with a magnetic moment pointing in a different direction. (It is possible that multiple domains may form, not just two.) This is separated from the first region by “domain walls”. These domain walls move, growing the second region at the expense of the first, until the moment of the entire MNF has reversed. This reversal process will also have a characteristic speed/ frequency of motion, which can appear in the ACS data.

### Torque Magnetometry:

Torque (τ) measurements were performed using a VSM (MicroSense, LLC, Lowell, MA) at room temperature in magnetic fields ranging from 4 kA m^−1^ (50 Oe) to a saturating magnetic field of 1.2 MA m^−1^ (15 kOe) with 16 measurements per magnetic field and angle combination. (The gain was fixed for all angles at each applied magnetic field, but could vary between different applied magnetic fields to account for the increase in signal.) The freshly made liquid samples sealed in polychlorotrifluoroethylene liquid capsules were used for torque measurements. The sample was held fixed with respect to the pick-up coils and the electromagnet (and therefore the applied magnetic field) was rotated (both clockwise [CW] and counterclockwise [CCW]) around the sample in steps of 5 degrees. This eliminated the artifacts associated with non-perfectly straight sample rods. The resulting torque curves (CW and CCW) for each applied magnetic field were each fit to the following phenomenological equation to extract the anisotropy energies:

(3) $$\tau =K_{1}V\text{sin}(\theta )+K_{2}V\text{sin}(2\theta )+K_{3}V\text{sin}(3\theta )+K_{4}V\text{sin}(4\theta )$$

where $K_{i}$ is the $i^{\text{th}}$ anisotropy energy density, V is the volume of magnetic material, and θ is the angle between the applied magnetic field and the sample magnetization. $K_{i}$ is only an effective value when the applied magnetic field is less than the saturation field for the sample. Data were normalized by total volume of magnetic material. (This means that if only the core is reversing in the CoNFs, the value calculated will be an underestimate.) Interpretation of this phenomenological model is linked to the physical structure of the colloid, not crystallographic axes of the ferrites as is commonly done for single crystals.^[42,53]^

### SANS:

**Figure 16.**
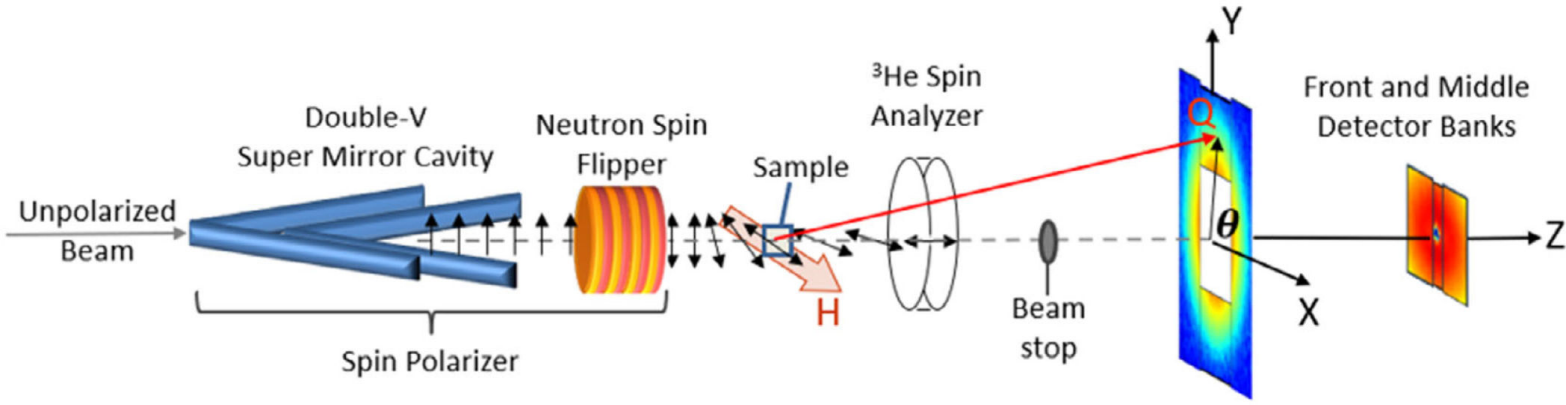
The schematic shows the components of the vSANS beamline along with their orientation relative to the *X*, *Y*, and *Z* axes. Note that vSANS has two 2D “picture frame” detectors that allow simultaneous detection of the scattered neutrons over an expansive *Q* range.

Unpolarized, half-polarized, and full-polarized SANS measurements at room temperature were performed at the NIST Center for Neutron Research (NCNR) using the Center for High Resolution Neutron Scattering (CHRNS) vSANS (very Small Angle Neutron Scattering) beamline (see Figure 16 for instrument schematic). The half-polarized (i.e., only the incident beam was polarization analyzed) measurements were performed on all samples suspended in H_2_O whereas the full polarization (i.e., both the incident beam and the scattered beam were polarization analyzed) measurements were limited to the FeNF-E and CoNF-D samples suspended in D_2_O to highlight the low-intensity magnetism. (The D_2_O SLD is nearly contrast matched to that of iron oxide.) The neutron wavelength was 0.6 nm with one guide in the incident neutron beam. The data were collected with two sets of staggered 2D detectors that spanned a range of scattering vectors (*Q*) from 2.5 × 10^−3^ to 1 × 10^−1^ Å^−1^. All raw 2D data were corrected for empty cell and background scattering (blocked beam) as well as for detector pixel efficiency. These data were adjusted for the sample transmission but were not scaled by the main beam intensity to generate the absolute scattered intensity since the nanoparticle concentration in the area illuminated by the beam may vary slightly with applied magnetic field.

The beam and magnetic field orientation relative to the 2D detector plane for polarization measurements is illustrated in the instrument schematic in Figure 16 and in the inset to Figure 5. Incident neutrons along the Z direction scatter from the sample in transmission geometry into the 2D detectors in the *X*-*Y* plane to measure the intensity (I). The wavevector *Q* is assumed to be in the detector plane. An electromagnet was used to apply magnetic fields in the *X* direction. All measurements were made at room temperature with the field increasing sequentially from 7 mT to 500 mT. Since SANS measurements are very time intensive, the measurements were performed at three selected magnetic fields of 7 mT, 41 mT, and 500 mT. These were chosen to encompass magnetic fields before and after the formation of chains and near saturation—see Figures 9 and 11. (Note that $B=\mu_{0}(M+H)$ in the SI where ${\text{μ}}_{0}$ is the permeability of free space.) The spin state of the incident neutrons, up (↑) or down (↓), was selected by a double-V supermirror cavity in combination with a radio-frequency (RF)-neutron spin flipper. The supermirror cavity and flipper have a combined polarization efficiency of 0.975 ± 0.009. For a small set of the measurements, the spin state of the scattered neutrons was selected using a ^3^He spin filter that preferentially transmits neutrons with spins aligned parallel to the polarized ^3^He atoms, while neutrons of the other spin state are absorbed. The polarization state of the ^3^He spin filter can also be reversed with a nuclear magnetic resonance pulse of an appropriate frequency. In this full polarization configuration, we measured the $I^{↑↑}$ and $I^{↓↓}$ non-spin flip cross sections as well as the $I^{↑↓}$ and $I^{↓↑}$ spin flip cross sections of the scattered neutrons. The full polarization data were additionally corrected for time-dependent polarization efficiency and transmission of ^3^He cell in accordance with previously described methods. [54] The half-polarized data ($I^{↑}$ and $I^{↓}$) were not corrected for the polarization efficiency of the supermirror in the incident beam as it is negligible. All data were reduced using vSANS Pol-Reduction software^[55]^ which also extracts 1D, area-normalized sector slices of ±15° (Figure 5a) taken about the horizontal *X*-axis and the vertical Y-axis.

To interpret the resulting SANS data, it is important to note that neutron selection rules dictate that the scattered neutrons are sensitive only to the projection of the magnetization that is perpendicular to Q. Additionally, polarized neutrons enable the separation of structural and magnetic scattering contributions as detailed elsewhere.^[56]^ Since magnetic scattering is a relatively small component of the total scattering, the field-dependence of the colloid structure $\text{(}N^{2}\text{)}$ was first characterized by adding the $I^{↑}$ and $I^{↓}$ cross sections of the half-polarized data (to extract the total unpolarized signal) and then conducting sector-specific analysis about the *X*- and *Y*-axes (Figure 5a) to highlight the formation of collective MNF superstructures oriented parallel and perpendicular to the applied field, respectively.

The Q dependence of the net magnetization parallel to the applied field $\left(M_{\text{parl}}\right)$ was obtained from either the half-polarized or the fully-polarized data using the following equation:

(4) $$M_{\text{parl}}^{2}={\left[I^{↓↓}(Y\text{axis})-I^{↑↑}(Y\text{axis})\right]}^{2}/4\left[I^{↑↑}(Y\text{axis})-I^{↓↓}(Y\text{axis})\right]$$

under the assumption that $I^{↓↓}\geq I^{↑↑}$. (Note that $I^{↑↑}\left(I^{↓↓}\right)$ is replaced with $I^{↑}\left(I^{↓}\right)$ in the case of half polarization.) As an example, Figure S6, Supporting Information, shows the individual $I^{↑}\left(I^{↓}\right)$ cross sections of the half-polarized data for FeNF-E in fields of 7 mT and 500 mT. A difference between $I^{↑}$ and $I^{↓}$ is evident in the sector cuts along the *Y*-axis, and $M_{\text{parl}}^{2}$ can be extracted from these cross sections using Equation (4). However, $I^{↑}$and $I^{↓}$ are equal for the sector cuts along the *X*-axis, consistent with neutron selection rules. The $M_{\text{parl}}^{2}$ results presented in this manuscript were extracted from similar half-polarized data for the samples suspended in H_2_O, but the *Q* dependence of $M_{\text{parl}}^{2}$ from the full-polarized data for the sample suspended in D_2_O was similar. The field-dependent magnitude of the scattering from these two datasets, however, differed, possibly due to partial precipitation of the MNFs out of the D_2_O solvent in high fields. As a result, the full polarization data could not be relied upon to provide accurate values of the MNF magnetization.

The magnetization perpendicular to the field $\text{(}M_{\text{perp}}\text{)}$ can be extracted only from the full polarization measurements. We focus here on:

(5) $$M_{\text{perp}}^{2}(Y\text{axis})=M_{Z}^{2}(Y\text{axis})=\left[I^{↑↓}(Y\text{axis})+I^{{↑}^{↓}}(Y\text{axis})\right]$$

which highlights the *Q* dependence of $M_{\text{perp}}$ along the Y-axis direction (i.e., perpendicular to the applied magnetic field direction), and on:

(6) $$M_{\text{perp}}^{2}(X\text{axis})=\left[M_{Y}^{2}(X\text{axis})+M_{Z}^{2}(X\text{axis})\right]/2=\left[I^{↑↓}(X\text{axis})+I^{↓↑}(X\text{axis})\right]/2$$

which describes the Q dependence of $M_{\text{perp}}$ along the *X*-axis direction (i.e., the applied magnetic field direction). Note that $M_{Y}$ and $M_{Z}$ represent the components of the magnetization along the perpendicular *Y*- and *Z*-directions, respectively. The data for these magnetization components are generally limited by signal-to-noise as the spin flip intensities are significantly smaller than the corresponding non-spin flip intensities.

Model functions were used to fit the $N^{2}$, $M_{\text{parl}}^{2}$ and $M_{\text{perp}}^{2}$ curves for each sample using SasView.^[57]^ The complexity of the intra- and inter-particle structure of the MNFs required a linear combination of multiple model functions. It is important to note that models used to parameterize SANS data are not unique due to a loss of phase information that is intrinsic to the measurement technique. In this case, we have chosen a model that captures the primary features in the data (such as the low-*Q* upturns and the correlation peaks), is self-consistent among all the field-dependent data for individual samples, minimizes the number of free parameters (to avoid overparameterization), and is consistent with the physical interactions that control the colloids. Since the parameters listed in Tables 2, 3, 5, and 6 are only relevant in the context of the chosen model, error estimates are not meaningful. For these measurements, the colloidal structures formed by multi-MNF clusters can best be modeled as interacting ellipsoids (Figure 7a). For $N^{2}$ and $M_{\text{parl}}^{2}$, the best fits were generated from a linear combination of an ellipsoid^[58]^ with a hard-sphere structure factor^[59]^ and a sphere.^[60]^ (Refer to Figure S4, Supporting Information, for an example of the breakdown of the contributions of these individual components to a typical $N^{2}$ fit and to Figure S5, Supporting Information, for a discussion of alternate models that were ruled out.) In general, the ellipsoid component accounts for the oblong shape of the individual MNFs as well as asymmetric clustering among multiple MNFs. (As an example, an ellipse approximation of a MNF dipole cluster is shown in Figure 7a.) The addition of the hard-sphere structure factor for interacting units mimics the local chaining or clustering among the nanoflowers with the “volume fraction” parameter related to the extent of the long-range order in this context. Twice the hard-sphere radius typically corresponds to the average center-to-center distance between the ellipsoids (Figure 7a). The additional sphere model accounts for individual grains that comprise the MNFs and are either physically detached and/or otherwise isolated. (These unattached or isolated grains are likely paramagnetic and do not contribute substantially, on an average, to the magnetic response of the entire system.)

Known parameters (such as SLDs) were fixed in the fits, and the fit ranges for several parameters were constrained to maintain consistency with other physical measurements as well as with the fits in different magnetic fields. The SLD for the iron oxide was held constant at 6.97 X 10^−6^ Å^−2^ and the SLD for H_2_O is assumed to be −0.5 × 10^−6^ Å^−2^. As stated previously, the MNFs for the full polarization measurements were suspended in D_2_O with a SLD of 6.37 × 10^−6^ Å^−2^, which is nearly matched to the iron oxide structural SLD. The polydispersity of the equator radius of the ellipse was assumed to be Gaussian and set to a value of 0.14 for both FeNF samples, and the polydispersity of the polar radius was set to 0.18 for FeNF-E and 0.14 for FeNF-D. For the CoNF samples, *N*^2^ was again fit to a linear combination of an ellipsoid^[58]^ with a hard-sphere structure factor^[59]^ and a sphere.^[60]^ The SLD for the CoNFs was held constant at 6.07 × 10^−6^ Å^−2^, and the polydispersities of the equator and polar radii were set to 0.13 and 0.10, respectively.

To extract the values of the magnetization reported in Tables 5 and 6, the fitted scale factor for $N^{2}$ in the corresponding field (Figures 6 and 8) was held constant in fits of $M_{\text{parl}}^{2}$. Contrasting with the structural fits, the model that best describes the $M_{\text{parl}}^{2}$ data for the CoNP nanoflowers is a core–shell ellipsoid^[61]^ in which the shell has reduced magnetization. Note that for all $M_{\text{parl}}^{2}$ data, it was not necessary to include an additional spherical term representing loose grains. For the hard-sphere structure factor multiplying the ellipsoid, the hard-sphere radii and volume fractions were fixed to the values obtained from the corresponding structural fit for that same sample (Tables 2 and 3), as the nanoflower-to-nanoflower distance is dictated by their physical separation. The resulting fitted volume fractions were small and comparable to those obtained in the structural fit. For FeNF-E and CoNF-E, the volume fraction was assumed to be zero to match the absence of structural correlations in the *Y*-direction.

Due to neutron beam time limitations, we investigated only FeNF-E and CoNF-D in full polarization, and we focus here on the field-dependent spin flip data (Figure 14), which are sensitive to the magnetization components perpendicular to the magnetic field $M_{\text{perp}}=M_{Y}=M_{Z}$. The resultant spin flip scattering, however, was weak and limited by signal-to-noise. To avoid issues with over-parameterization in the fits, we modeled the data with a simple sphere (with no polydispersity) to obtain an estimate of the dominant/average magnetic length scale (i.e., sphere radius) for the perpendicular magnetization components.

### OOMMF:

The FeNFs and CoNFs were modeled with variable saturation magnetization $M_{\text{s}}$, (with different values for both the core and shell if appropriate), magnetic anisotropy K (type, sign, and magnitude), total magnetic volume diameter d, shell thickness if appropriate, spacing between MNFs, and exchange energy A. Values for these parameters are constants and chosen to be representative of the different MNFs, and also include bulk values for comparison (see Figure SI-11). If not specified, the exchange energy is *A* = 13.2 pJ/m (corresponding to bulk for magnetite), d = 28 nm, and the spacing is 1 nm. The bulk $M_{\text{s}}$ for magnetite is 480 kA/m. The calculated energy (see Figure SI-12) also includes contributions from exchange (which depends upon the angle between neighboring spins), demagnetization (as a shape anisotropy), and Zeeman, but the exchange is four orders of magnitude smaller than the other two. The demagnetization and Zeeman are comparable in magnitude, but the demagnetization is small compared to the magnetic anisotropy and is nearly constant for each chain assembly; i.e., the energy at H=0 is pretty close to the demagnetization energy across all the data points. (This is presumably not the case during reversal, but Figure 15 only shows equilibrium states.) The simulations were run with the MNFs discretized with 1 nm cells; the magnetization is found to be nearly uniform inside each MNF.Balls were chosen as the model of the MNFs because the MNFs are generally spherical in shape from both TEM and SANS, and the SANS data could be modeled as single domains. While this ball model is overly simplistic, it captures key aspects of the behavior of the MNFs with respect to magnetization reversal. The MNFs are actually comprised of perhaps non-spherical grains which would have a non-zero demag (shape anisotropy), and it is possible that the addition of individual grains to each MNF in the model might lead to predictions with greater quantitative accuracy. However, we do not know experimentally and quantitatively what the detailed shapes of the grains are or what exchange coupling there is between the grains. It is too big a space to model without more guidance from the experiments. For this initial work, we kept the models simple to avoid over-parameterization.

### SLP:

A prototype AMF heating device called “HYPER” (Magnetic Insight, Inc., Alameda, CA), that spatially confines regions of MNF activation^[62]^ was used to measure the SLP. The heater consisted of two opposing permanent magnets and an AMF-generating solenoid coil surrounding a sample chamber. The permanent magnets generate a field-free region, centered on the sample chamber, which spatially confines the heating, that is, MNFs within the region are free to interact with the AMF and generate heat, while moments of MNFs outside the region are saturated by the static magnetic field and do not generate heat. For these measurements, the permanent magnets were adjusted to create a region which completely encompassed the 1 mL sample. By adjusting the capacitance, the solenoid can generate a variable amplitude AMF (0–12 kA m–^1^) at a set frequency of 340 kHz. The magnetic field amplitude was calibrated with an AMF probe (AMF Life Systems, Auburn Hills, MI). The rate of temperature rise was measured in 1.0 s intervals with a fiber-optic temperature probe (Micronor Sensors, Inc., Ventura, CA).

We estimated SLP from measured time-temperature data using a modified form of the well-known Box–Lucas equation as previously described.^[63]^ Briefly, 1 mL volumes of aqueous MNF suspension and water (blank) were prepared and placed in standard 1.5 mL polystyrene test tubes and inserted into a 3D-printed holder. Samples were then placed within the HYPER solenoid and heated. Heating experiments were repeated at several peak amplitude settings between 4 kA m^−1^ and 12 kA m^−1^, all at a frequency of 340 kHz. Power during heating was pulsed, each with 1 min duration and 50% duty (30 s on and 30 s off) for a total of 20 pulses. The selected amplitude and frequency range is within the Hergt–Dutz criteria (5 × 10^−9^ A m^−1^ s^−1^).^[64]^ Temperatures measured from water blanks were subtracted from sample temperatures to correct for calorimeter heat capacity before subtracting the start temperature for each experiment from the temperature measured at each time step. The resulting temperature change was then fit to the modified Box–Lucas model, using methods previously described.^[63]^ SLP was calculated for each pulse, then averaged. The error bars denote one standard deviation.

### PSF:

PSF is the derivative of the *M* vs. *H* loop at a specific frequency and peak amplitude AMF. A Momentum Magnetic Particle Imaging scanner (Magnetic Insight, Inc., Alameda, CA) was used to measure the PSF for each MNF sample using methods previously described.^[27]^ PSF data was collected using the RELAX module which analyzes the net magnetization of the MNF sample between –127 kA m^−1^ and +127 kA m^−1^ with a 16 kA m^−1^ excitation field at a drive frequency of 45 kHz. Sample were prepared by dispersing MNFs in water (at a concentration of 5 μg of Fe in 1 mL of deionized water) via mechanical mixing. It should be noted that the height of the PSF correlates to the change in measured magnetic flux within the excitation field, while the full width at half maximum (FWHM) corresponds to the theoretical spatial resolution of the MNFs.

### Statistical Analysis:

For data that has error bars, the average value $\left(\bar{X}\right)$ and standard deviation (S, which represents 1σ) are calculated using the equations shown below: When the measured parameter is number of counts (i.e., SANS and XRD), counting statistics are used. In this case, the reported value is the number of counts (N) and the standard deviation is $S^{2}=N$.


(7) $$\bar{X}=\left(\frac{1}{n}\right)\sum_{k=1}^{n}x_{k}$$

(8) $$S^{2}=\left(\frac{1}{n-1}\right)\sum_{k=1}^{n}{\left(x_{k}-\bar{X}\right)}^{2}$$

## Supplementary Material

Supinfo

Supporting Information

Supporting Information is available from the Wiley Online Library or from the author.

## Figures and Tables

**Table 1.** List of FeNF and CoNF MNF samples examined here with names, lot numbers, coating, concentration, and measurements performed. (Note: in the sample names, “D” refers to dextran and “E” refers to electrostatic).

Sample Name	Lot Number	Coating	Concentration (mg of Fe per mL of water)	Measurements in Manuscript
FeNF-E	17 719 103–02 (Synomag)	None	10	TEM, DLS, XRD, *M* vs *H*, *M* vs *T*, ACS, torque, SANS, half- and full-Pol. SANS, SLP, PSF
FeNF-D	17 819 104–02 (Synomag-D)	Dextran	10	TEM, DLS, XRD, *M* vs *H*, *M* vs *T*, ACS, torque, SANS, half-Pol. SANS, SLP, PSF
CoNF-E	17 419 123–02 (CoFe_2_O_4_)	None	10	TEM, DLS, XRD, *M* vs *H*, *M* vs *T*, ACS, torque, SANS, half-Pol. SANS, SLP, PSF
CoNF-D	18 119 124–02 (CoFe_2_O_4_-D)	Dextran	10	TEM, DLS, XRD, *M* vs *H*, *M* vs *T*, ACS, torque, SANS, half- and full-Pol. SANS, SLP, PSF

**Table 2.** Parameters for the fits to the structural SANS data in Figure 6 for FeNF-E and FeNF-D in fields of 7 mT, 41 mT, and 500 mT. The (approximate) equaor and polar radii refer to the dimensions of the nanoflowers (or nanoflower clusters) along the ellipsoid width and length, respectively. The hard-sphere diameter (i.e., twice the radius listed in this table) approximates the average center-to-center spacing from one nanoflower (or nanoflower cluster) to the next. The volume fraction, which must be less than 1, provides a relative gauge of the extent of the structural correlation among the nanoflowers (or nanoflower clusters). The scale factor multiplies the added spherical form factor that approximates the scattering from loose or unbound grains. Note that the radii of these grains were fit for the 500 mT data along the field direction and were held constant at 5.50 nm for FeNF-E and 5.24 nm for FeNF-D for the remainder of the fits.

			Parallel to *Y*-axis			

Sample	Field [mT]	Equator Radius [nm]	Polar Radius [nm]	Hard Sphere Radius [nm]	Volume Fraction	Scale Factor for Sphere

FeNF-E	7	13.3	27.8	N/A	0	0.029
	41	13.1	34.5	N/A	0	0.026
	500	13.3	43.5	N/A	0	0.020
	7	13.8	25.0	43.9	0.038	0.014
FeNF-D	41	13.7	24.7	44.0	0.032	0.027
	500	13.5	25.8	42.4	0.020	0.014
		Parallel to *X*-axis and Magnetic Field				

Sample	Field [mT]	Equator Radius [nm]	Polar Radius [nm]	Hard Sphere Radius [nm]	Volume Fraction	Scale Factor for Sphere

FeNF-E	7	14.5	42.2	26.2	0.14	0.092
	41	15.7	43.5	28.4	0.20	0.12
	500	16.5	45.0	29.3	0.28	0.10
	7	15.2	26.6	43.9	0.093	0.079
FeNF-D	41	15.9	26.9	43.6	0.11	0.11
	500	15.8	28.8	42.4	0.13	0.089

**Table 3.** Parameters for the fits to the structural SANS data in Figure 8 for CoNF-E and CoNF-D in fields of 7, 41, and 500 mT. The (approximate) equator and polar radii refer to the dimensions of the nanoflowers (or nanoflower clusters) along the ellipsoid width and length, respectively. The hard-sphere diameter (i.e., twice the radius listed in this table) approximates the average center-to-center spacing from one nanoflower (or nanoflower cluster) to the next. The volume fraction, which must be less than 1, provides a relative gauge of the extent of the structural correlation among the nanoflowers (or nanoflower clusters). The scale factor multiplies the added spherical form factor that approximates the scattering possibly from loose or unbound grains. While these radii are not constant with field (5–7 nm), the fits are not particularly sensitive to this parameter.

			Parallel to *Y*-axis				

Sample	Field [mT]	Equator Radius [nm]	Polar Radius [nm]	Hard Sphere Radius [nm]	Volume Fraction	Scale Factor for Sphere	Sphere Radius [nm]

CoNF-E	7	12.2	24.0	N/A	0	0.066	5.9
	41	12.3	26.9	N/A	0	0.075	7.3
	500	12.4	40.4	N/A	0	0.056	6.4
	7	13.3	26.3	10.7	0.061	0	N/A
CoNF-D	41	13.4	27.3	11.0	0.069	0	N/A
	500	13.6	29.0	10.9	0.080	0	N/A
		Parallel to *X*-axis and Magnetic Field					

Sample	Field [mT]	Equator Radius [nm]	Polar Radius [nm]	Hard Sphere Radius [nm]	Volume Fraction	Scale Factor for Sphere	Sphere Radius [nm]

CoNF-E	7	12.3	45.9	22.4	0.15	0.086	5.4
	41	12.6	50.8	23.9	0.18	0.12	5.2
	500	13.6	75.4	25.3	0.29	0.14	5.5
	7	12.9	23.0	38.2	0.078	0.075	6.3
CoNF-D	41	13.2	22.7	38.2	0.087	0.10	5.8
	500	13.3	22.4	38.0	0.11	0.12	5.8

**Table 4.** Magnetic anisotropy constants at saturation from torque magnetometry measurements (Figure S10, supporting information) normalized by total volume of magnetic material assuming the bulk density of magnetite of 5.2 g cm^−3^ (1.86 × 10^−10^ m^−3^ for the FeNF samples and 2.83 × 10^−10^ m^−3^ for the CoNF sample). *K*_1_ is the unidirectional anisotropy; *K*_2_ is the uniaxial anisotropy; *K*_3_ is the trigonal anisotropy; K_4_ is the cubic anisotropy. As the magnetocrystalline anisotropy for the ferrite is cubic, *K*_4_ is therefore the magnetocrystalline anisotropy constant. The last column is the saturation magnetization (*M*_S_) from the DC hysteresis loops. Error bars represent 1*σ*.

Sample	*K*_1_ [kJ m^−3^]	*K*_2_ [kJ m^−3^]	*K*_3_ [kJ m^−3^]	*K*_4_ [kJ m^−3^]	*M*_S_ [kA m^−1^]
FeNF-E	1.83 ± 0.05	7.90 ± 0.05	3.39 ± 0.05	1.02 ± 0.05	287.987 ± 0.024
FeNF-D	1.99 ± 0.05	8.98 ± 0.05	3.82 ± 0.05	0.86 ± 0.05	338.210 ± 0.032
CoNF-E	1.38 ± 0.04	4.10 ± 0.04	2.69 ± 0.04	0.67 ± 0.04	141.361 ± 0.011
CoNF-D	1.20 ± 0.04	2.33 ± 0.04	2.30 ± 0.04	0.71 ± 0.04	236.419 ± 0.022

**Table 5.** Parameters for the fits to the half-polarized SANS data corresponding to the net magnetization squared (Figure 12) for FeNF-E and FeNF-D in fields of 7 mT, 41 mT, and 500 mT. These data were obtained from sector slices parallel to the *Y*-axis. The equator and polar radii refer to the dimensions of the ellipsoidal magnetized regions within nanoflower clusters along the ellipsoid width and length, respectively. The scale factors for the scattering from the nanoflower ellipsoids were held constant to match the values obtained from the structural fits (Figure 6). The hard-sphere radii were constrained to match the values in Table 2 for FeNF-D, and the volume fraction for FeNF-E was fixed at zero. The magnetic scattering length density is proportional to the net magnetization along the field, which is also listed in units of kA m^−1^. Note that the magnetization from the loose, unbound grains was determined from fits to be zero in all the cases listed in the table.

Sample	Field [mT]	Equator Radius [nm]	Polar Radius [nm]	Hard Sphere Radius [nm]	Volume Fraction	Magnetic SLD (×10^−6^ A^−2^, kA m^−1^)
FeNF-E	7	12.9	23.4	N/A	0	0.78, 279
	41	12.6	39.9	N/A	0	0.91, 314
	500	13.1	39.9	N/A	0	0.99, 340
FeNF-D	7	13.0	19.8	43.9	0.075	0.75, 258
	41	13.0	21.0	44.0	0.028	0.88, 303
	500	13.2	21.9	42.4	0.058	0.97, 334

**Table 6.** Parameters for the fits to the half-polarized SANS data corresponding to the net magnetization squared (Figure 13) for CoNF-E and CoNF-D in fields of 7, 41, and 500 mT. These data were obtained from sector slices parallel to the *Y*-axis. The best fits were obtained for a core/shell ellipsoid with reduced magnetization in the shell. The equator and polar radii (shell thickness) refer to the dimensions of the ellipsoidal magnetic core (shell thickness) within nanoflower clusters along the ellipsoid width and length, respectively. The scale factors for the scattering from the nanoflower ellipsoids were held constant to match the values obtained from the structural fits (Figure 8). The hard-sphere radii and volume fractions for CoNF-D were fixed to the values obtained from the corresponding structural fits (Table 3), and the volume fraction for CoNF-E was set to zero consistent with the structural fits. The magnetic scattering length density is proportional to the net magnetization along the field, which is also listed in units of kA m^−1^. Note that the magnetization from the loose, unbound grains was determined from fits to be zero in all the cases listed in the table. We note that there appears to be a process of slow, remagnetization in high fields of CoNF-D which could be responsible for some of the observed differences in anisotropy.

Sample	Field [mT]	Equator Radius [nm]	Polar Radius [nm]	Equator Shell Thick. [nm]	Polar Shell Thick. [nm]	Mag. SLD Core (×10^−6^ A^−2^, kA m^−1^)	Mag. SLD Shell (×10^−6^ A^−2^, kA m^−1^)
CoNF-E	7	9.62	17.1	4.4	4.4	0.89, 308	0.39, 134
	41	9.00	21.7	4.8	5.5	1.1, 388	0.44, 150
	500	9.19	32.4	4.4	5.3	1.3, 436	0.54, 187
CoNF-D	7	9.80	18.6	4.4	4.6	0.93, 321	0.39,134
	41	10.2	19.7	4.3	3.8	1.0, 347	0.43, 148
	500	12.1	21.3	2.6	2.6	1.1, 364	0.40, 137

## Data Availability

The data that support the findings of this study are available from the corresponding author upon reasonable request.
