# Supplementary material for: Magnetic Anisotropy Dominates over Physical and Magnetic Structure in Performance of Magnetic Nanoflowers
Source: Small Struct. Author manuscript; Available in PMC 2026 Jun 11. (PMC13251730; doi:10.1002/sstr.202400410)
Supplement: Supinfo [file NIHMS2016749-supplement-Supinfo.pdf]

Supplementary Information:
Magnetic Anisotropy Dominates Over Physical and Magnetic Structure in Performance of Magnetic Nanoflowers

Julie Borchers[1], Kathryn Krycka[1], Brianna Bosch-Santos[2], Eduardo de Lima Correa[2,3], Anirudh Sharma[4], Hayden Carlton[4], Yanliu Dang[2], Michael Donahue[5], Cordula Grüttner[6], Robert Ivkov[4,7-9], Cindi L. Dennis[2*]

[1]NIST Center for Neutron Research, National Institute of Standards and Technology, Gaithersburg, MD 20899-6102, USA

[2]Material Measurement Laboratory, National Institute of Standards and Technology, Gaithersburg, MD 20899-8552 USA

[3]Theiss Research, La Jolla, CA 92037, USA

[4]Department of Radiation Oncology and Molecular Radiation Sciences, Johns Hopkins University School of Medicine, Baltimore, MD 21231, USA

[5]Information Technology Laboratory, National Institute of Standards and Technology, Gaithersburg, MD 20899USA

[6]micromod Partikeltechnologie, GmbH, 18057 Rostock, Germany

[7]Department of Oncology, Sydney Kimmel Comprehensive Cancer Center, Johns Hopkins University School of Medicine, Baltimore, MD 21231, USA

[8]Department of Mechanical Engineering, Whiting School of Engineering, Johns Hopkins University, Baltimore, MD 21218-2681, USA

[9]Department of Materials Science and Engineering, Whiting School of Engineering, Johns Hopkins University, Baltimore, MD 21218-2681, USA

Tables:
Table SI-1: Hydrodynamic diameter and poly-dispersity index measured by DLS directly after synthesis. (Values in brackets are from repeated measurements two years after synthesis to check stability.)

Figures:
Figure SI-1 shows the raw DLS data for (a) FeNF-E, (b) FeNF-D, (c) CoNF-E, and (d) CoNF-D. Average values from fits are listed in Table SI-1.

Figure SI-2 shows the temperature dependence of the AC susceptibility for FeNF-D, which has a peak that decreases in intensity as the temperature is increased from 290 K to 320 K.

Figure SI-3 shows the temperature dependence of the ACS for the CoNF-D, which has a similar, but sharper, peak around 20-30 Hz that decreases with increasing temperature.

Figure SI-4 - Contributions of the individual model components to the fit of the N^2 data (blue dots) along the *X*-axis direction from half-polarized scattering for FeNF-E in a 500 mT field. The blue line corresponds to the final fit shown in Fig. 6c) in the main text. The red dashed line shows the fit from only the ellipse form factor multiplied by the hard sphere structure factor (i.e., with the scale factor for the loose grain contributions set to zero). The purple dot-dash line is from only the spherical form factor that describes the loose grains (i.e., with the scale factor for the ellipse form factor set to zero). The green dotted line shows the fit from the form factors for the ellipse and loose grains assuming that the hard sphere structure factor does not multiply the former (i.e., scattering is from individual,

uncorrelated ellipses). This plot demonstrates the necessity of including a hard sphere structure factor to describe the peaks evident in the *X*-axis data at higher fields.

Figure SI-5 – Example of alternate models considered for fits of N^2 data. The data (hollow blue dots) shown in both panels are for FeNF-E in a 500 mT field obtained from half-polarized scattering along the *Y*-axis. The blue lines correspond to the final fit, shown in Fig. 6c) in the main text, for the model with an ellipse form factor plus a sphere form factor describing the loose grains. The red line in (a) corresponds to the best fit with a form factor for a single sphere. The fitted radius for the sphere is 16.6 nm, which is between the values of the equator and polar radii (13.3 nm and 27.8 nm, respectively) obtained for the ellipse model used in Fig. 6c) and Table 2 in the main text. The chi-squared for the single sphere model fit is very high. The fit clearly misses the data at low *Q* and the fall-off is too steep at higher *Q*. The red line in (b) corresponds to a fit with a form factor for two spheres. The fitted radii for these spheres are 14.8 nm and 27.8 nm, the latter of which equals the dimension of the polar radius obtained for the ellipse model (Fig. 6c and Table 2) and the former of which is larger than the equator radius. The chi-squared for the two sphere model is lower than that obtained for the sphere model in (a) but higher than that obtained for the ellipse model. However, the two sphere model still undercuts the data in the intermediate *Q* region. Similar explorations of alternate models were performed on all of the other SANS data sets, and the ellipse model detailed in the main text fit them all consistently and generated the best chi-squared. Other Sasview models with chaining and/or clustering were considered for data sets that showed a correlation peak, but the ellipsoid form factor multiplied by the hard sphere structure factor model proved to be the best choice and the most self-consistent.

Figure SI-6 - SANS measurements of $I^{\uparrow}$ (purple) and $I^{\downarrow}$ (green) from half-polarized scattering for FeNF-E in magnetic fields of (a), (c) 7 mT and (b), (d) 500 mT. These data correspond to sector cuts of the two-dimensional SANS data (Fig. 5 in main text) along (a), (b) the *Y* axis perpendicular to the magnetic field and along (c), (d) the *X* axis parallel to the field. The structural scattering (N^2) in Figs. 6 and 8 in the main text was extracted by adding $I^{\uparrow}$ and $I^{\downarrow}$ for each field condition. The net magnetization parallel to the field (M^2_{parl}) in Figs. 12 and 13 was obtained from data similar to these for each field using equation 4.

Figure SI-7 shows the *Q* dependence of M_{perp} along the *X* and *Y* directions (top and bottom row, respectively) for CoNF-D in D_2O in a field of 7 mT. Note that the signal-to-noise of these data are limited since they were obtained from full-polarized SANS measurements. As indicated in the main text, the fits to a simple sphere model give radii values of approximately 12 nm along both the *X* and *Y* axes. For these coated MNPs, the transverse magnetic domains extend beyond a single grain but not beyond a single nanoflower.

Figure SI-8 - Measured SLP vs AMF peak amplitude for newer vials of FeNF -E, FeNF-D, CoNF-E, and CoNF-D. SLP was estimated from calorimetry data measured with an AMF frequency of 340 kHz for all MNPs. Data shown represent mean SLP values and error bars represent one standard deviation.

Figure SI-9 - PSF measurements for newer vials of FeNF-E, FeNF-D, CoNF-E, and CoNF-D. (a) Representative comparison of the PSF for each MNP. PSF (b) amplitude and (c) FWHM for each MNP. (The peak for CoNF-D was not distinct enough to measure the FWHM.) Each dot (in (b) and (c)) represents a repeated measurement (3 in total) and the line refers to the median value.

Figure SI-10 – Raw torque data with fits for (a) FeNF-E, (b) FeNF-D, (c) CoNF-E and (d) CoNF-D. Error bars are shown and represent 1σ, but may be smaller than the symbol. Resulting fit parameters are provided in Table 4 in main text.

Figure SI-11 OOMMF modeling under different combinations of saturation magnetization (M_S) values, values and type of magnetic anisotropy (*K*), diameter (*d*) of the total magnetic volume (including shell thickness if appropriate) and spacing between MNFs. Values are chosen to be representative of the different MNFs. If not specified, the exchange energy is A = 13.2 pJ/m. Only half of the hysteresis loop is shown. Each color represents a different chain configuration, but the same parameters.
(a) Assembly key
(b) Ms = 300 kA/m, K = -1 kJ/m^3 (cubic), d=28 nm, 1 nm spacing
(c) Ms = 300 kA/m, K = -1 kJ/m^3 (cubic), d=28 nm, 15 nm spacing
(d) Ms = 340 kA/m, K = +6 kJ/m^3 (cubic), d=20 nm, spacing = 2 nm
(e) Ms = 340 kA/m, K = -6 kJ/m^3 (cubic), d=20 nm, spacing = 2 nm

(f) Ms = 426 kA/m, K = +7.9 kJ/m^3 (cubic), d=20 nm, spacing = 2 nm
(g) Ms = 426 kA/m, K = -7.9 kJ/m^3 (cubic), d=20 nm, spacing = 2 nm
(h) Ms = 480 kA/m, K = 0 kJ/m^3, d=20 nm, spacing = 2 nm
(i) Ms = 385 (core)/165 (shell) kA/m, 17 nm core, d = 26 nm, A = 13.2/7.6 (interface) pJ/m, K = 0, 1 nm spacing
(j) Ms = 385 (core)/165 (shell) kA/m, 17 nm core, d = 26 nm, A = 13.2/7.6 (interface) pJ/m, K = 0, 12 nm spacing.

Figure SI-12 Total energy and individual energy terms are plotted on the left axis showing the relative contribution of each to the total. Also plotted (on the right axis) is the normalized magnetization, showing the partial reversal process for the 5 chain, N=27 hcp curve shown in Fig. 15.

Figure SI-13 - Direct comparison of the XRD data for the four samples. Inset shows a close-up of the primary peak at 35.7 ^{o} in 2ϑ showing the slight shift of the CoNF peak relative to the FeNF peak.

References

[1] "Sasview Core Shell Ellipsoid," https://www.sasview.org/docs/user/models/core_shell_ellipsoid.html
[2] "Sasview Core Shell Sphere," https://www.sasview.org/docs/user/models/core_shell_sphere.html

Table SI-1: Hydrodynamic diameter and poly-dispersity index measured by DLS directly after synthesis. (Values in brackets are from repeated measurements two years after synthesis to check stability.)

Sample Name	***Lot Number***	***Diameter (nm)***	***Poly-Dispersity Index***
FeNF-E	17719103-02 (Synomag)	44.6 [49.7]	0.065 [0.119]
FeNF-D	17819104-02 (Synomag-D)	59.2 [60.5]	0.050 [0.026]
CoNF-E	17419123-02 ($CoFe_2O_4$)	44.4 [46.3]	0.142 [0.151]
CoNF-D	18119124-02 ($CoFe_2O_4$-D)	55.1 [55.8]	0.081 [0.054]

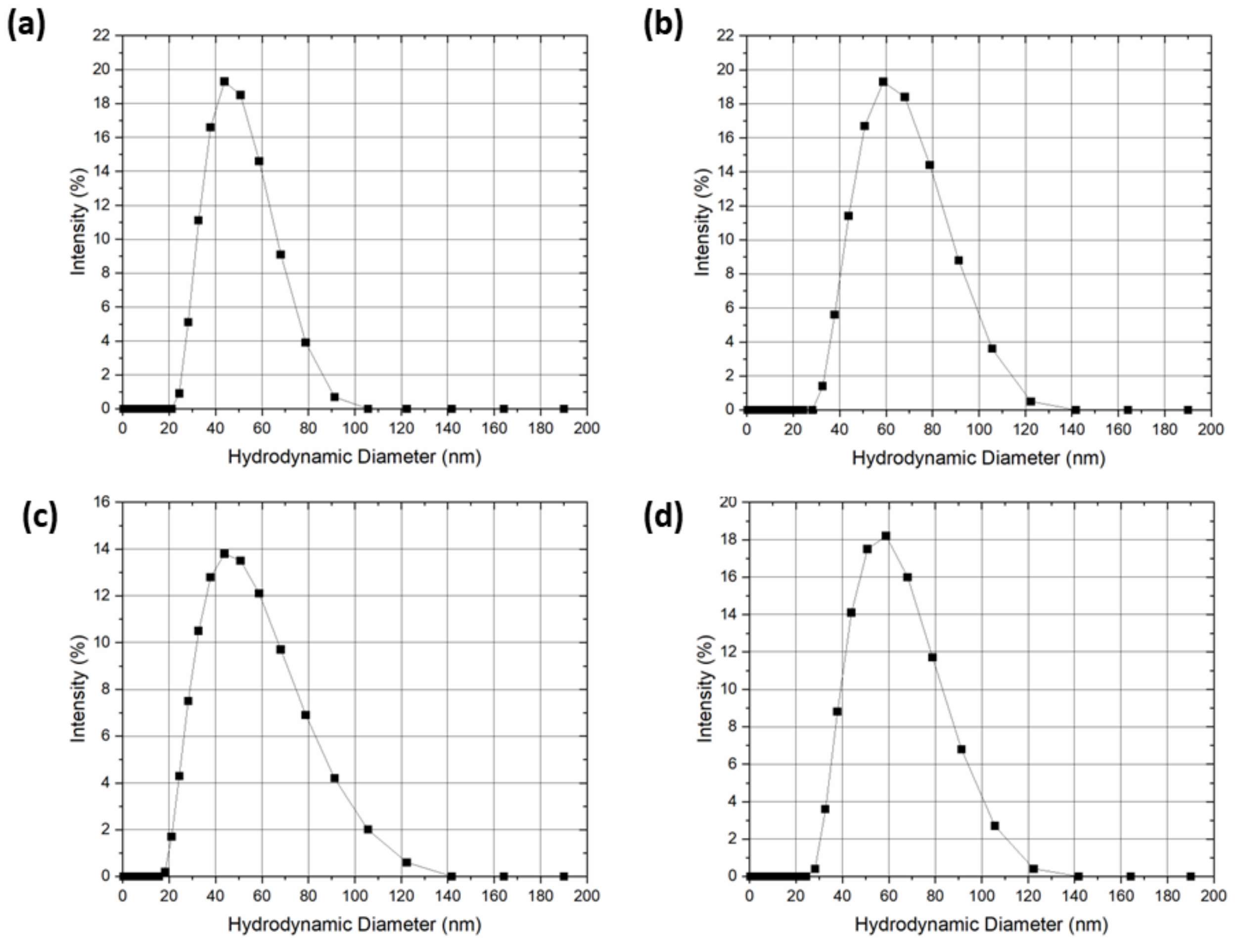

Figure SI-1 shows the raw DLS data for (a) FeNF-E, (b) FeNF-D, (c) CoNF-E, and (d) CoNF-D. Average values from fits are listed in Table SI-1.

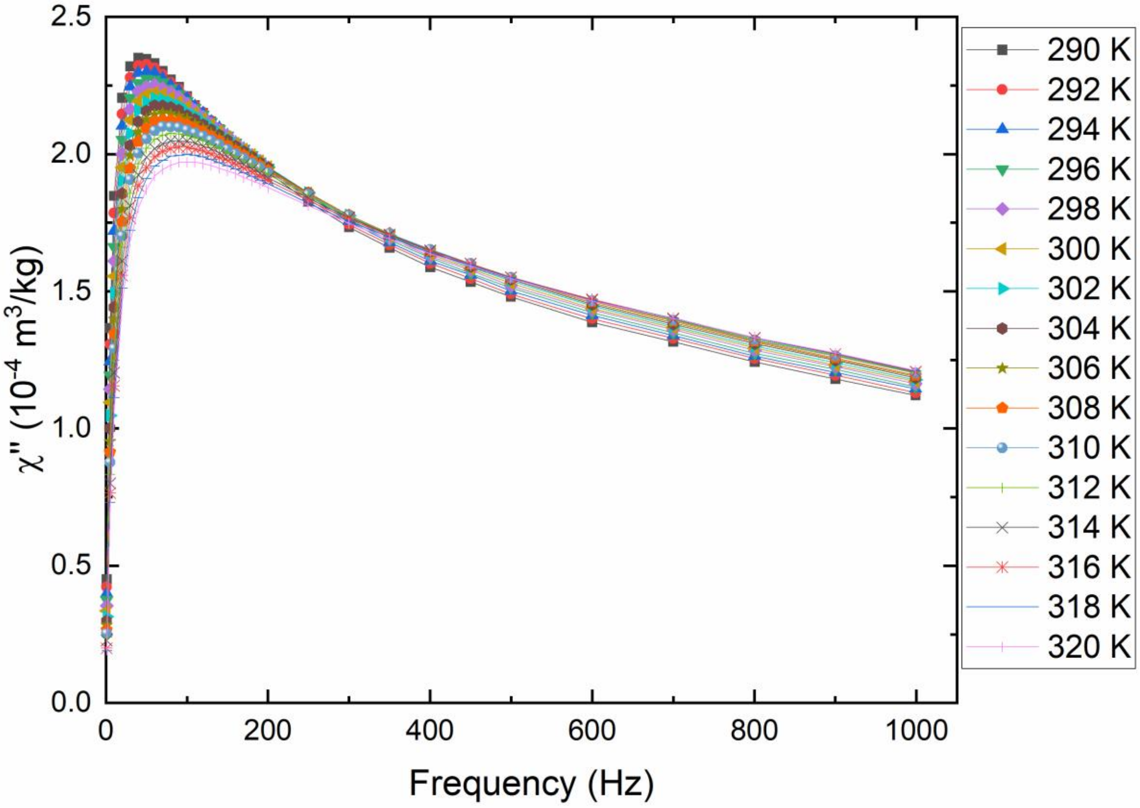

Figure SI-2 - Imaginary part of the AC susceptibility is plotted as a function of frequency and temperature for the FeNF-D. Error bars are shown and represent 1σ, but may be smaller than the symbol.

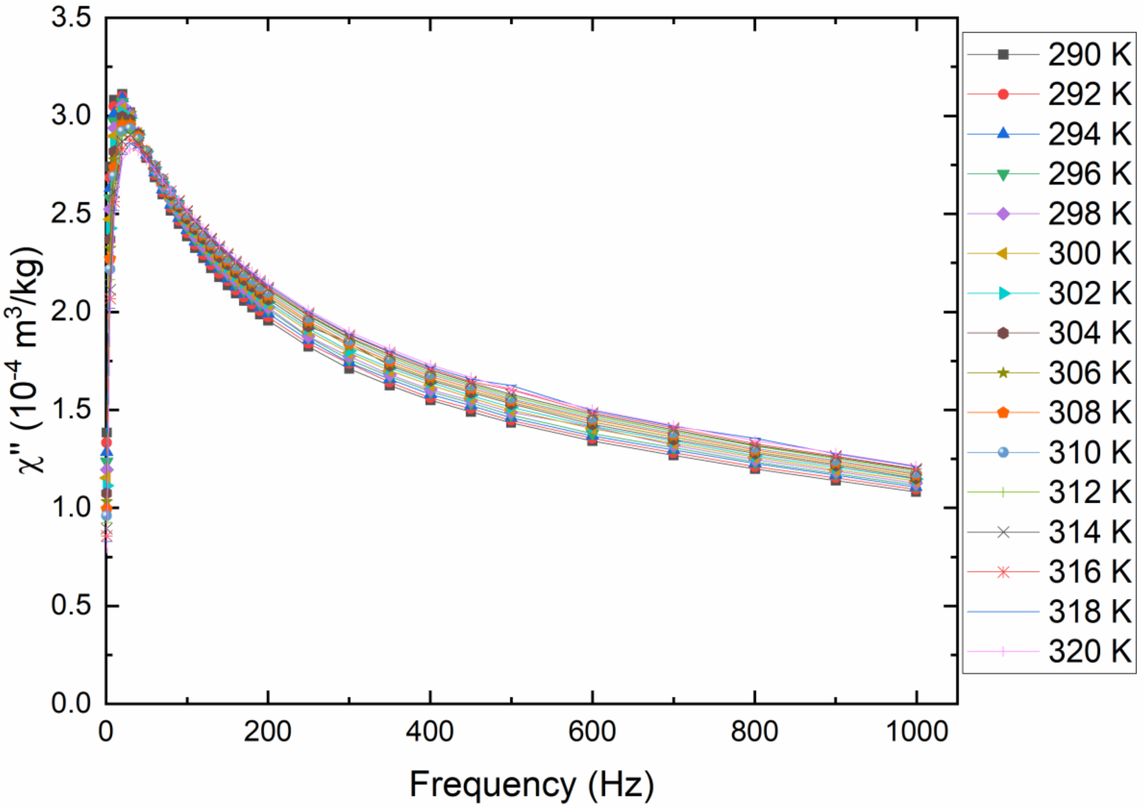

Figure SI-3 - Imaginary part of the AC susceptibility is plotted as a function of frequency and temperature for the CoNF-D. Error bars are shown and represent 1σ, but may be smaller than the symbol.

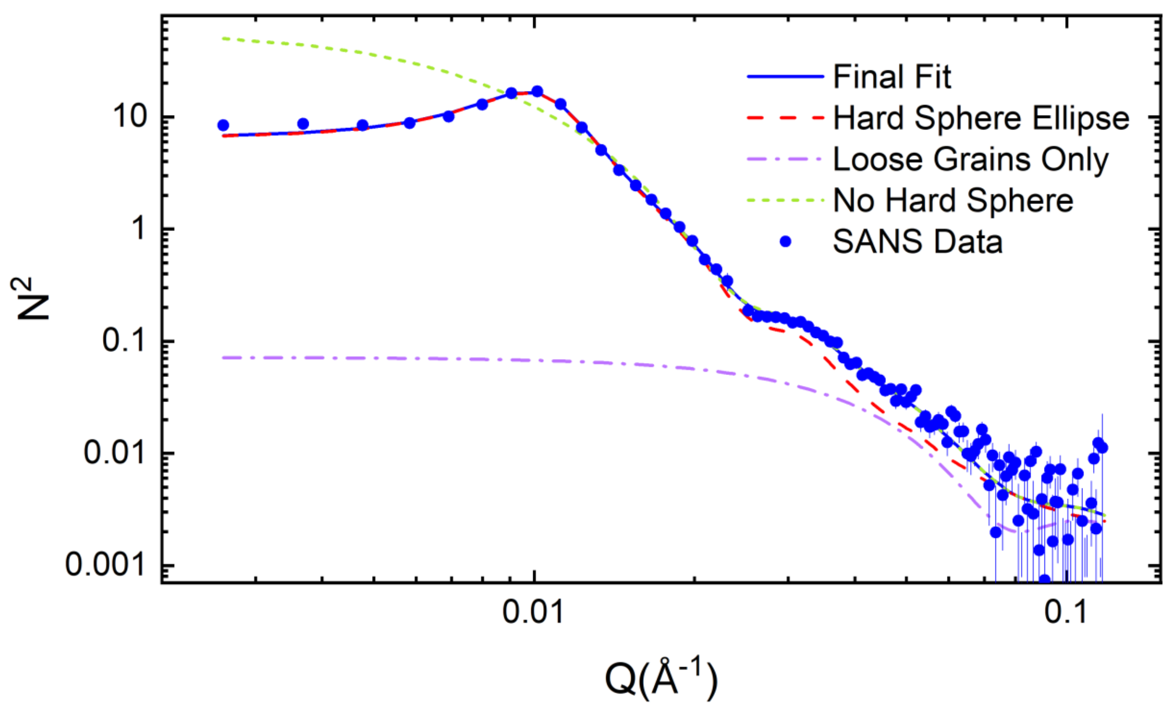

Figure SI-4 - Contributions of the individual model components to the fit of the N^2 data (blue dots) along the X-axis direction from half-polarized scattering for FeNF-E in a 500 mT field. The blue line corresponds to the final fit shown in Fig. 6c) in the main text. The red dashed line shows the fit from only the ellipse form factor multiplied by the hard sphere structure factor (i.e., with the scale factor for the loose grain contributions set to zero). The purple dot-dash line is from only the spherical form factor that describes the loose grains (i.e., with the scale factor for the ellipse form factor set to zero). The green dotted line shows the fit from the form factors for the ellipse and loose grains assuming that the hard sphere structure factor does not multiply the former (i.e., scattering is from individual, uncorrelated ellipses). This plot demonstrates the necessity of including a hard sphere structure factor to describe the peaks evident in the X-axis data at higher fields.

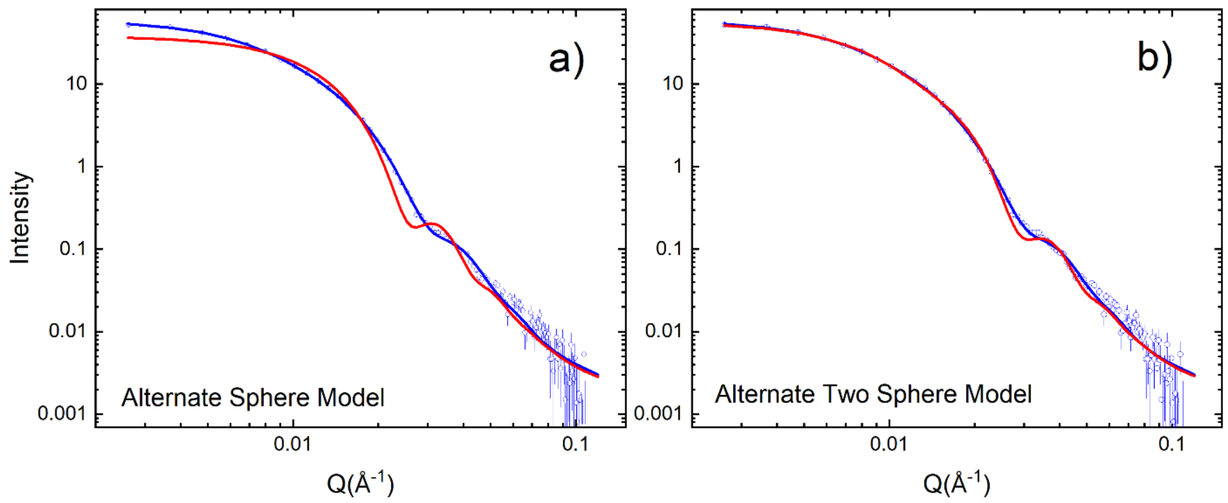

Figure SI-5 - Example of alternate models considered for fits of N^2 data. The data (hollow blue dots) shown in both panels are for FeNF-E in a 500 mT field obtained from half-polarized scattering along the *Y*-axis. The blue lines correspond to the final fit, shown in Fig. 6c) in the main text, for the model with an ellipse form factor plus a sphere form factor describing the loose grains. The red line in (a) corresponds to the best fit with a form factor for a single sphere. The fitted radius for the sphere is 16.6 nm, which is between the values of the equator and polar radii (13.3 nm and 27.8 nm, respectively) obtained for the ellipse model used in Fig. 6c) and Table 2 in the main text. The chi-squared for the single sphere model fit is very high. The fit clearly misses the data at low *Q* and the fall-off is too steep at higher *Q*. The red line in (b) corresponds to a fit with a form factor for two spheres. The fitted radii for these spheres are 14.8 nm and 27.8 nm, the latter of which equals the dimension of the polar radius obtained for the ellipse model (Fig. 6c and Table 2) and the former of which is larger than the equator radius. The chi-squared for the two sphere model is lower than that obtained for the sphere model in (a) but higher than that obtained for the ellipse model. However, the two sphere model still undercuts the data in the intermediate *Q* region. Similar explorations of alternate models were performed on all of the other SANS data sets, and the ellipse model detailed in the main text fit them all consistently and generated the best chi-squared. Other Sasview models with chaining and/or clustering were considered for data sets that showed a correlation peak, but the ellipsoid form factor multiplied by the hard sphere structure factor model proved to be the best choice and the most self-consistent.

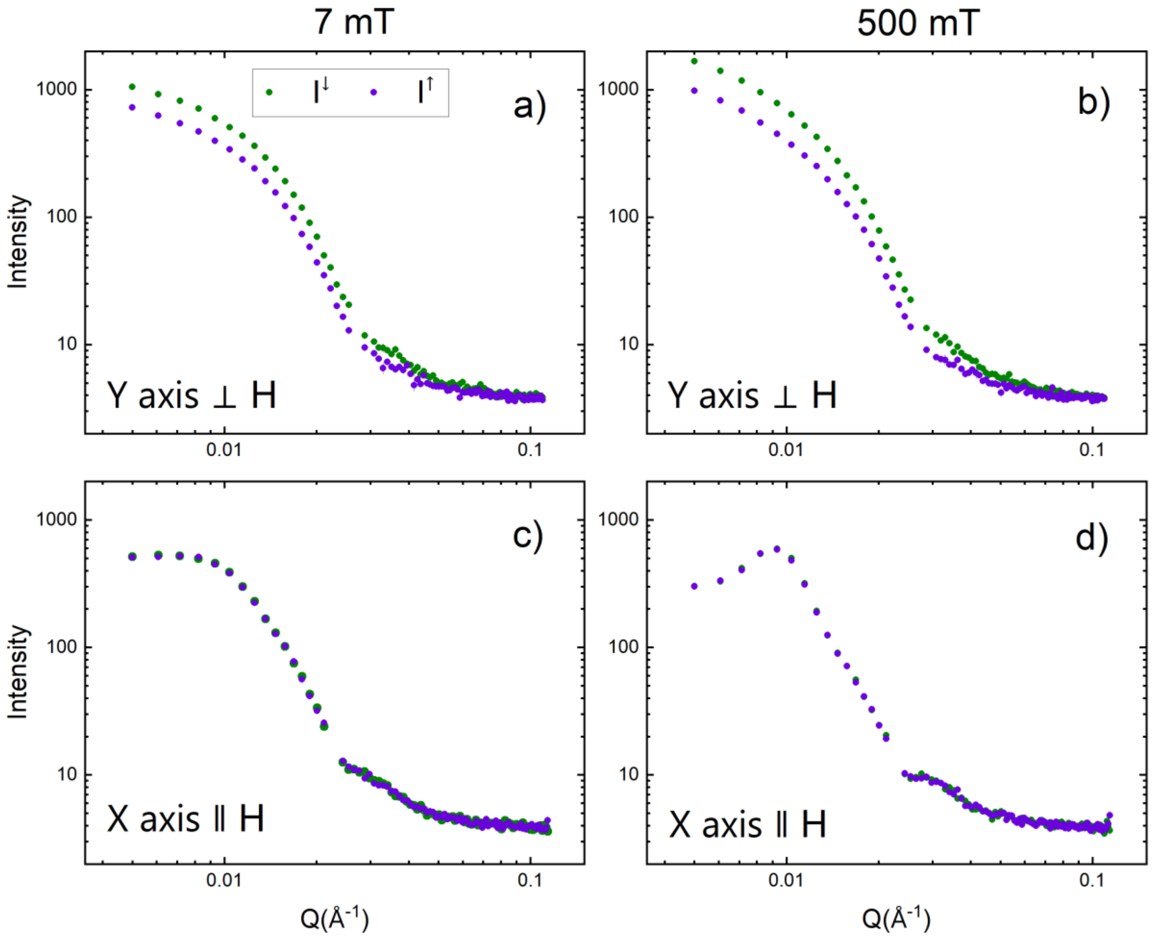

Figure SI-6 - SANS measurements of $I^{\uparrow}$ (purple) and $I^{\downarrow}$ (green) from half-polarized scattering for FeNF-E in magnetic fields of (a), (c) 7 mT and (b), (d) 500 mT. These data correspond to sector cuts of the two-dimensional SANS data (Fig. 5 in main text) along (a), (b) the *Y* axis perpendicular to the magnetic field and along (c), (d) the *X* axis parallel to the field. The structural scattering (N^2) in Figs. 6 and 8 in the main text was extracted by adding $I^{\uparrow}$ and $I^{\downarrow}$ for each field condition. The net magnetization parallel to the field (M^2_{parl}) in Figs. 12 and 13 was obtained from data similar to these for each field using equation 4.

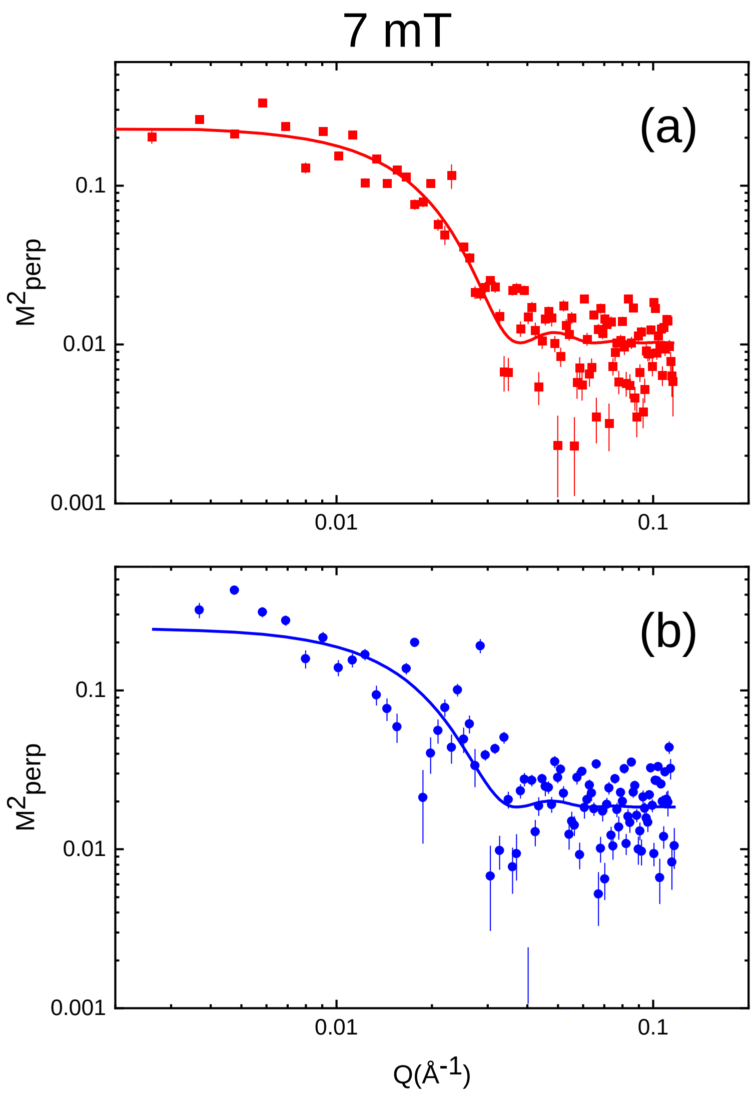

Figure SI-7 - Square of the net magnetization perpendicular to the applied field (along the *X* direction) obtained from full-polarized SANS measurements and corresponding fits to a spherical function at room temperature for the CoNF-D in D_2O in a field of 7 mT. The red curve in (a) corresponds to scattering as seen along the *X* direction, and the blue curve in (b) corresponds to scattering along the *Y* direction. M^2_{perp} is in arbitrary units. Error bars are shown and represent 1σ, but may be smaller than the symbol. Resulting fit parameters are provided in Table 6 in main text.

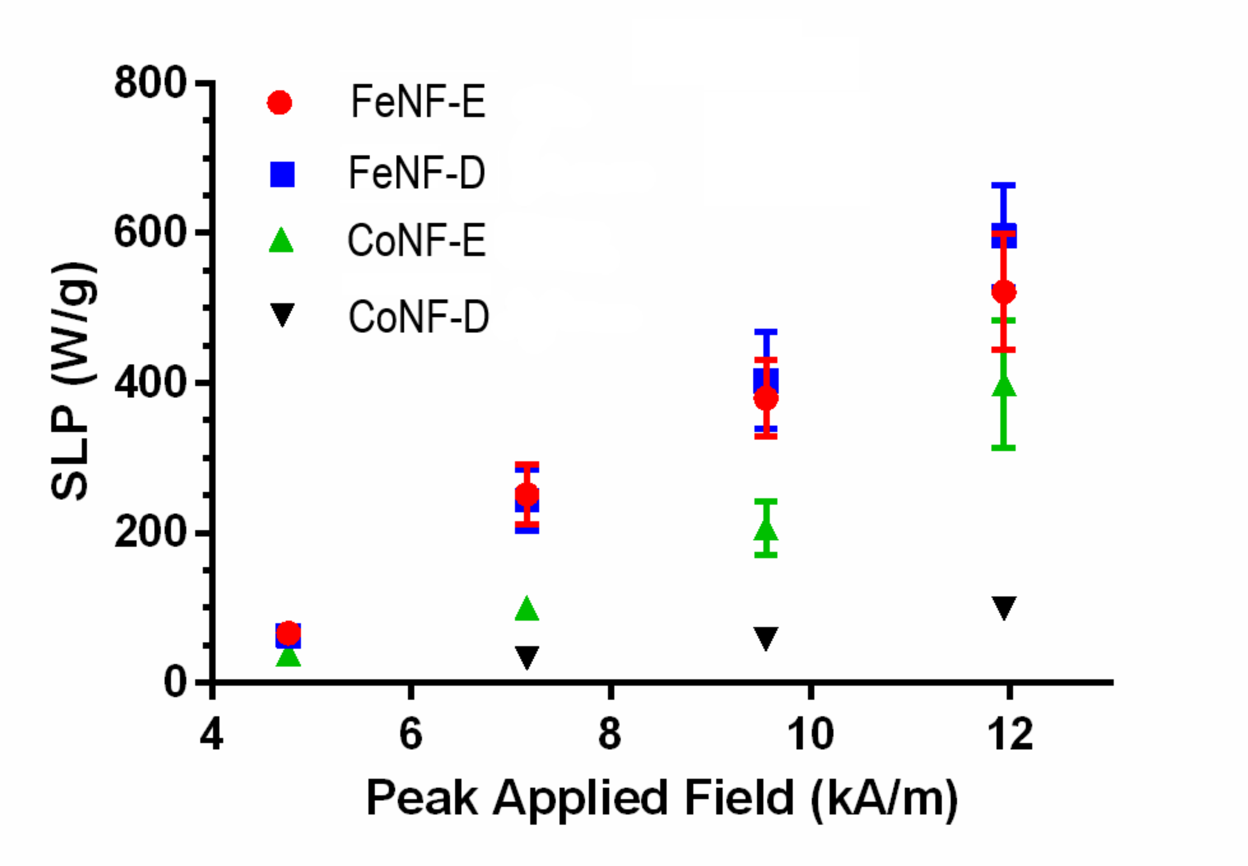

Figure SI-8 - Measured SLP vs AMF peak amplitude for newer vials of FeNF -E, FeNF-D, CoNF-E, and CoNF-D. SLP was estimated from calorimetry data measured with an AMF frequency of 340 kHz for all MNPs. Data shown represent mean SLP values and error bars represent one standard deviation.

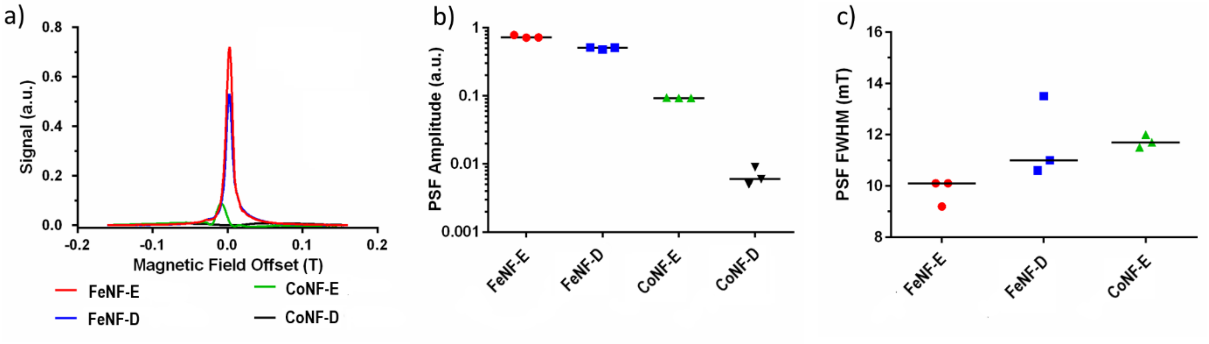

Figure SI-9 - PSF measurements for newer vials of FeNF-E, FeNF-D, CoNF-E, and CoNF-D. (a) Representative comparison of the PSF for each MNP. PSF (b) amplitude and (c) FWHM for each MNP. (The peak for CoNF-D was not distinct enough to measure the FWHM.) Each dot (in (b) and (c)) represents a repeated measurement (3 in total) and the line refers to the median value.

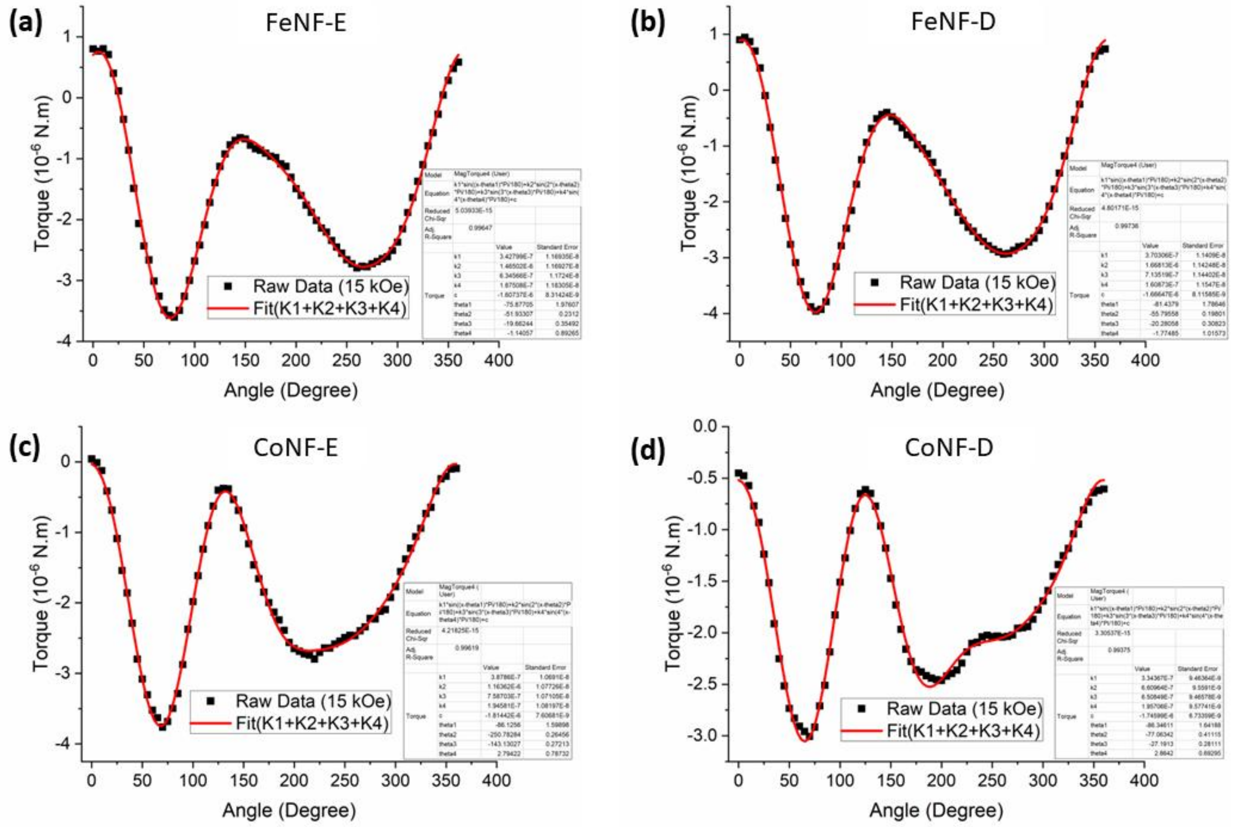

Figure SI-10 – Raw torque data with fits for (a) FeNF-E, (b) FeNF-D, (c) CoNF-E and (d) CoNF-D. Error bars are shown and represent 1σ, but may be smaller than the symbol. Resulting fit parameters are provided in Table 4 in main text.

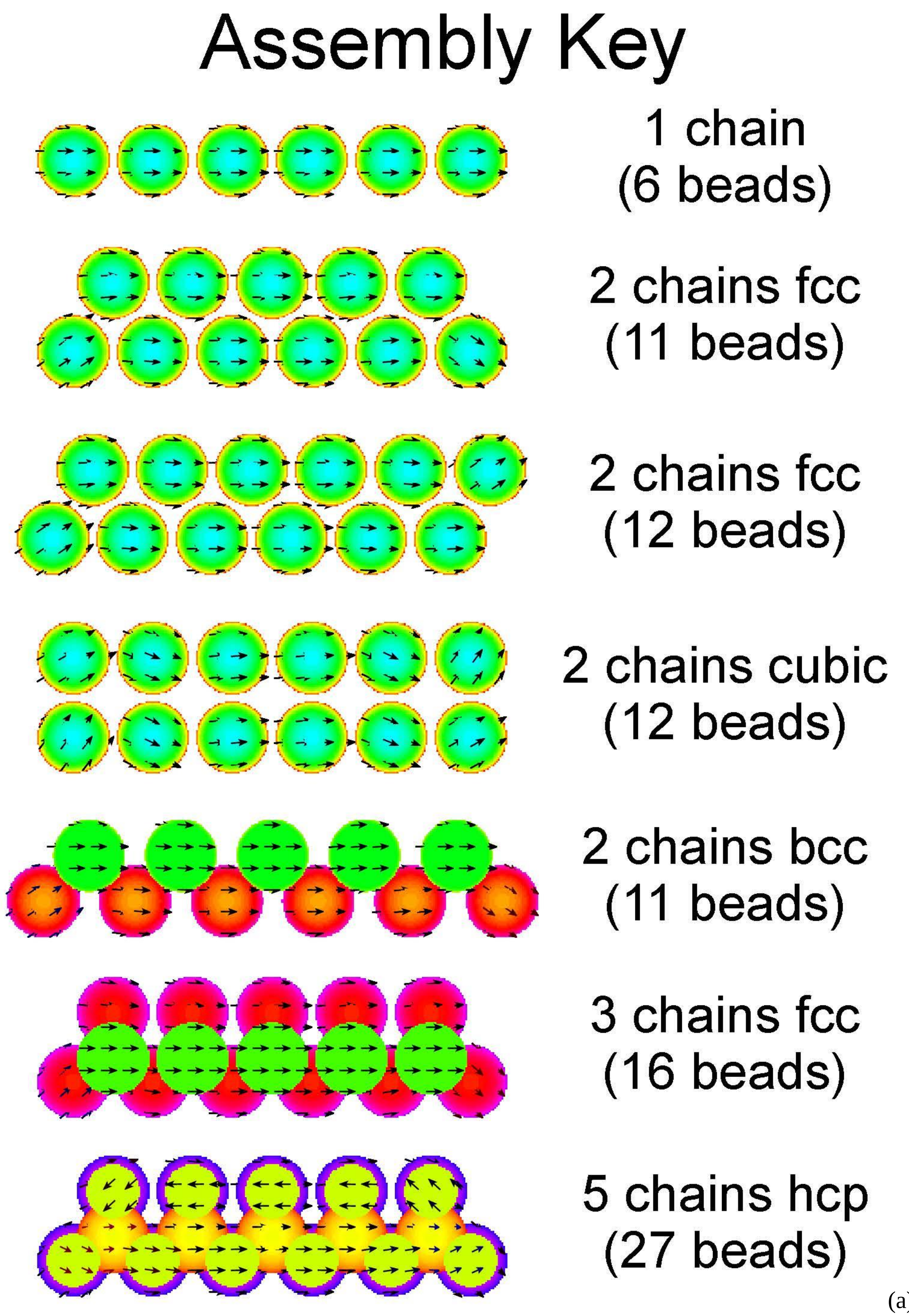
Assembly Key
1 chain
(6 beads)
2 chains fcc
(11 beads)
2 chains fcc
(12 beads)
2 chains cubic
(12 beads)
2 chains bcc
(11 beads)
3 chains fcc
(16 beads)
5 chains hcp
(27 beads)
(a)

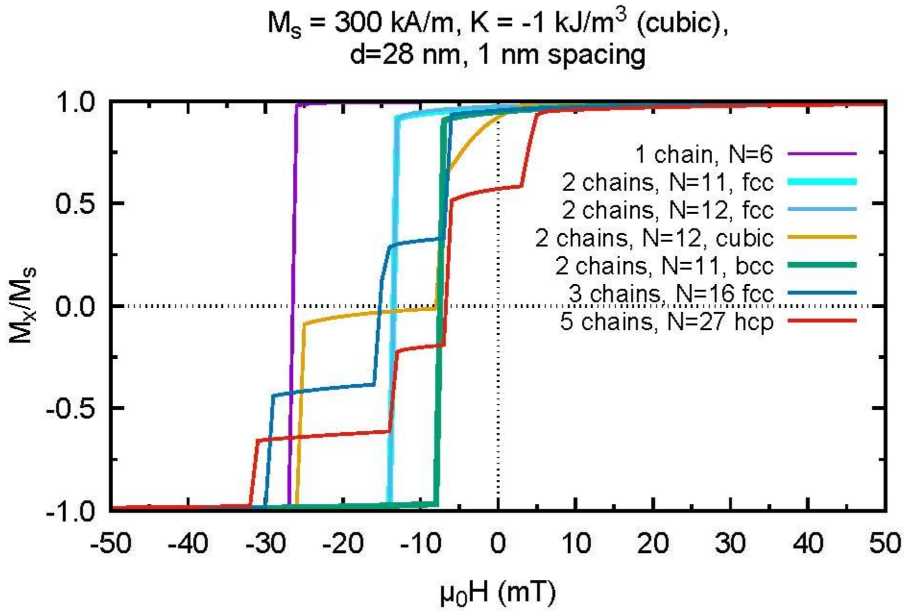

M_s = 300 kA/m, K = -1 kJ/m^3 (cubic),
d=28 nm, 1 nm spacing
1 chain, N=6
2 chains, N=11, fcc
2 chains, N=12, fcc
2 chains, N=12, cubic
2 chains, N=11, bcc
3 chains, N=16 fcc
5 chains, N=27 hcp
M_x/M_s
$\mu_0 H$ (mT)

(b)

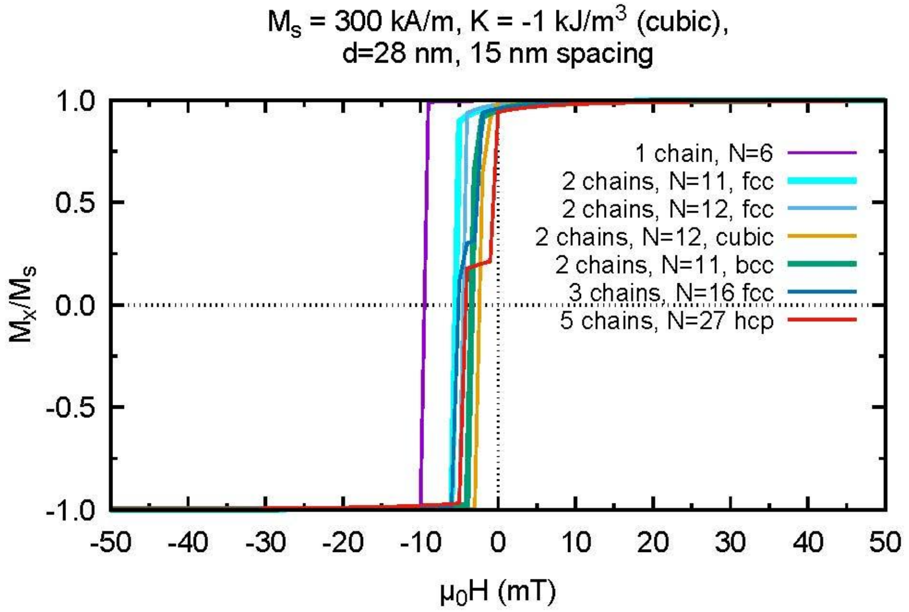

M_s = 300 kA/m, K = -1 kJ/m^3 (cubic),
d=28 nm, 15 nm spacing
1 chain, N=6
2 chains, N=11, fcc
2 chains, N=12, fcc
2 chains, N=12, cubic
2 chains, N=11, bcc
3 chains, N=16 fcc
5 chains, N=27 hcp
M_x/M_s
$\mu_0 H$ (mT)

(c)

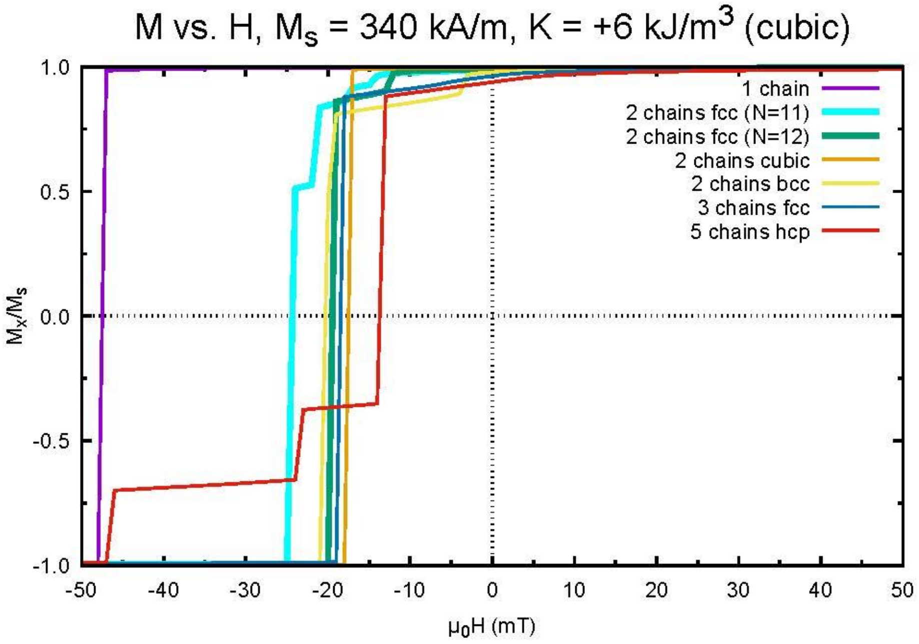
M vs. H, M_s = 340 kA/m, K = +6 kJ/m^3 (cubic)
1 chain
2 chains fcc (N=11)
2 chains fcc (N=12)
2 chains cubic
2 chains bcc
3 chains fcc
5 chains hcp
M_x/M_s
$\mu_0 H$ (mT)
1.0
0.5
0.0
-0.5
-1.0
-50
-40
-30
-20
-10
0
10
20
30
40
50

(d)

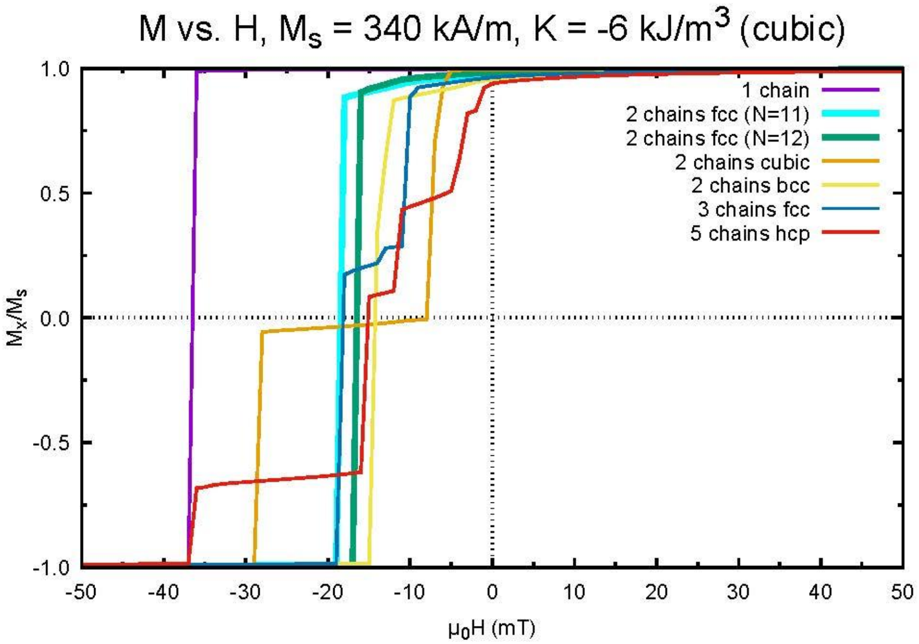
M vs. H, M_s = 340 kA/m, K = -6 kJ/m^3 (cubic)
1 chain
2 chains fcc (N=11)
2 chains fcc (N=12)
2 chains cubic
2 chains bcc
3 chains fcc
5 chains hcp
M_x/M_s
$\mu_0 H$ (mT)
1.0
0.5
0.0
-0.5
-1.0
-50
-40
-30
-20
-10
0
10
20
30
40
50

(e)

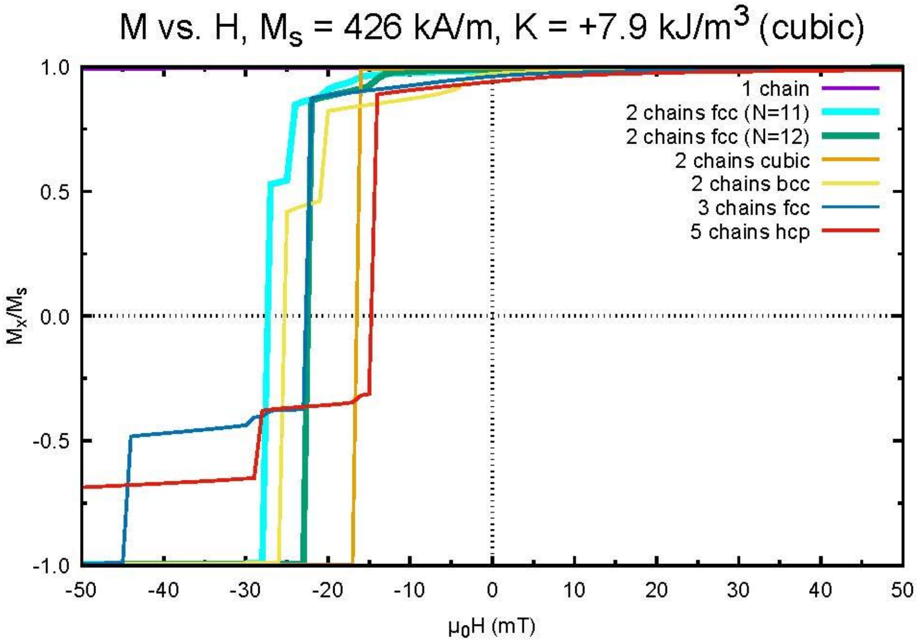
M vs. H, M_s = 426 kA/m, K = +7.9 kJ/m^3 (cubic)
1 chain
2 chains fcc (N=11)
2 chains fcc (N=12)
2 chains cubic
2 chains bcc
3 chains fcc
5 chains hcp
M_x/M_s
μ_0H (mT)

(f)

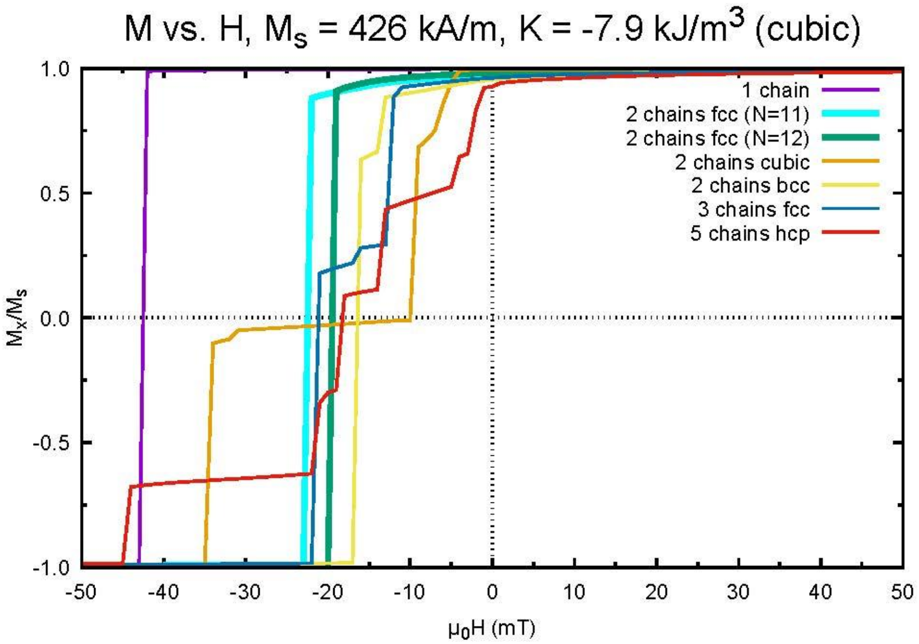
M vs. H, M_s = 426 kA/m, K = -7.9 kJ/m^3 (cubic)
1 chain
2 chains fcc (N=11)
2 chains fcc (N=12)
2 chains cubic
2 chains bcc
3 chains fcc
5 chains hcp
M_x/M_s
μ_0H (mT)

(g)

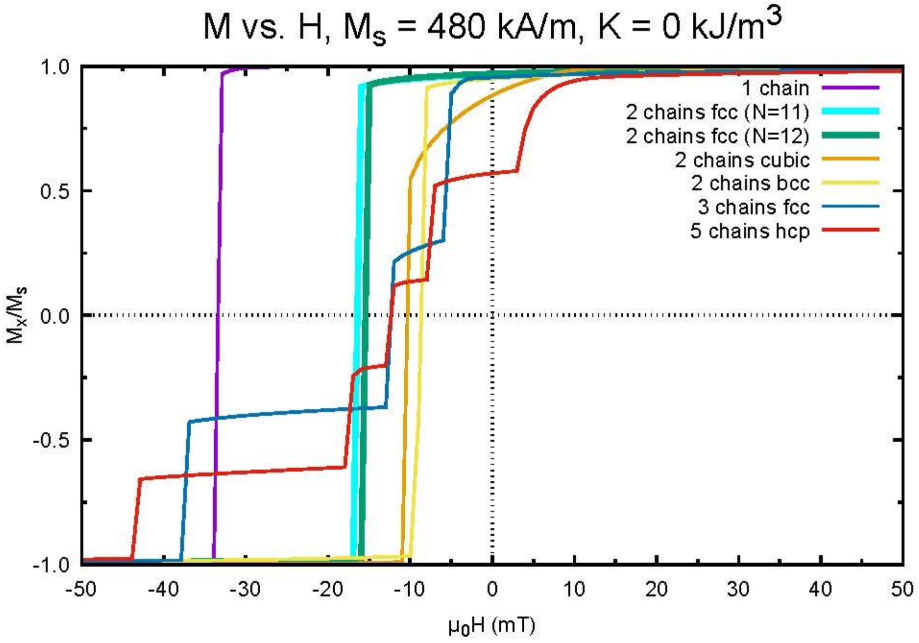

M vs. H, Ms = 480 kA/m, K = 0 kJ/m3
1 chain
2 chains fcc (N=11)
2 chains fcc (N=12)
2 chains cubic
2 chains bcc
3 chains fcc
5 chains hcp
Mx/Ms
μ0H (mT)

(h)

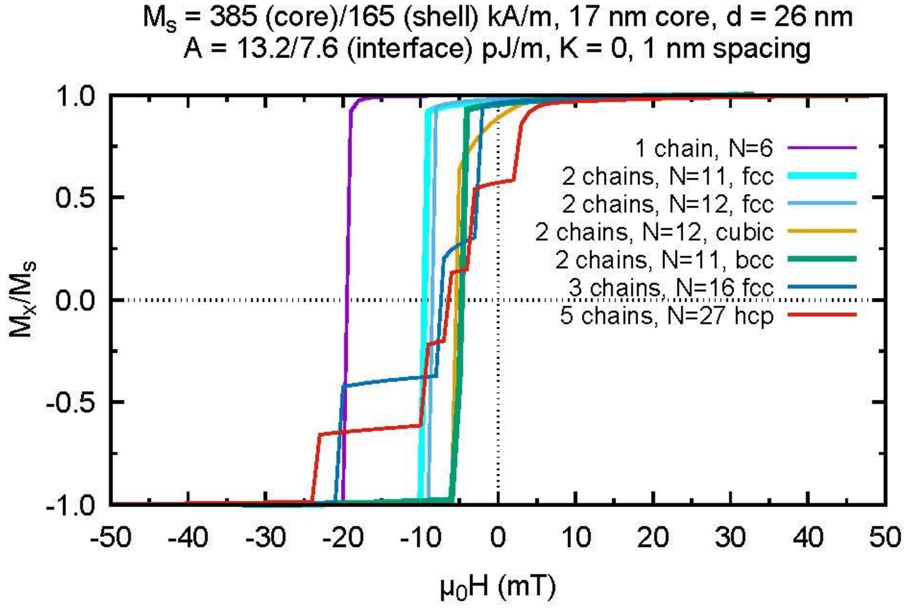

Ms = 385 (core)/165 (shell) kA/m, 17 nm core, d = 26 nm
A = 13.2/7.6 (interface) pJ/m, K = 0, 1 nm spacing
1 chain, N=6
2 chains, N=11, fcc
2 chains, N=12, fcc
2 chains, N=12, cubic
2 chains, N=11, bcc
3 chains, N=16 fcc
5 chains, N=27 hcp
Mx/Ms
μ0H (mT)

(i)

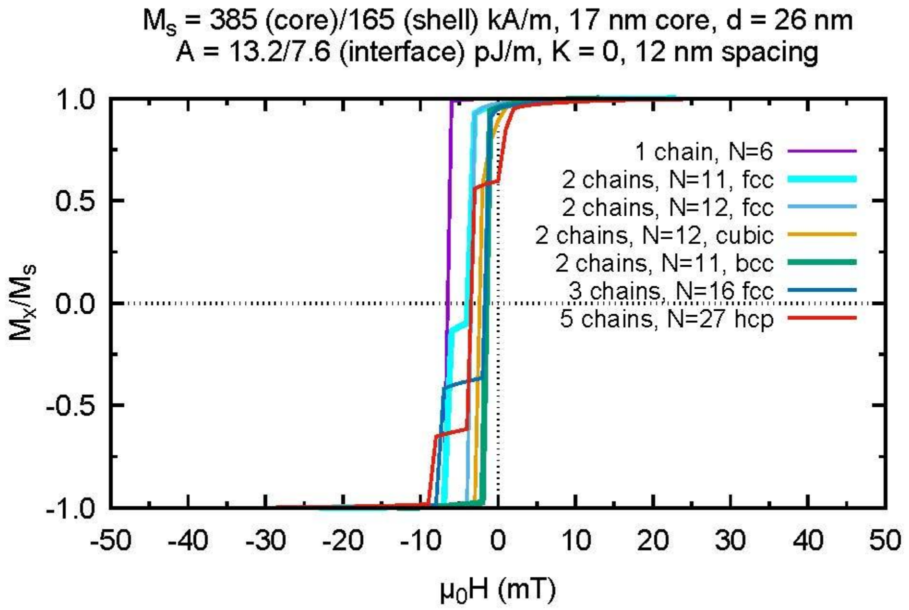

(j)

Figure SI-11 OOMMF modeling under different combinations of saturation magnetization (M_S) values, values and type of magnetic anisotropy (*K*), diameter (*d*) of the total magnetic volume (including shell thickness if appropriate) and spacing between MNFs. Values are chosen to be representative of the different MNFs. If not specified, the exchange energy is A = 13.2 pJ/m. Only half of the hysteresis loop is shown. Each color represents a different chain configuration, but the same parameters.

(a) Assembly key
(b) Ms = 300 kA/m, K = -1 kJ/m^3 (cubic), d=28 nm, 1 nm spacing
(c) Ms = 300 kA/m, K = -1 kJ/m^3 (cubic), d=28 nm, 15 nm spacing
(d) Ms = 340 kA/m, K = +6 kJ/m^3 (cubic), d=20 nm, spacing = 2 nm
(e) Ms = 340 kA/m, K = -6 kJ/m^3 (cubic), d=20 nm, spacing = 2 nm
(f) Ms = 426 kA/m, K = +7.9 kJ/m^3 (cubic), d=20 nm, spacing = 2 nm
(g) Ms = 426 kA/m, K = -7.9 kJ/m^3 (cubic), d=20 nm, spacing = 2 nm
(h) Ms = 480 kA/m, K = 0 kJ/m^3, d=20 nm, spacing = 2 nm
(i) Ms = 385 (core)/165 (shell) kA/m, 17 nm core, d = 26 nm, A = 13.2/7.6 (interface) pJ/m, K = 0, 1 nm spacing
(j) Ms = 385 (core)/165 (shell) kA/m, 17 nm core, d = 26 nm, A = 13.2/7.6 (interface) pJ/m, K = 0, 12 nm spacing.

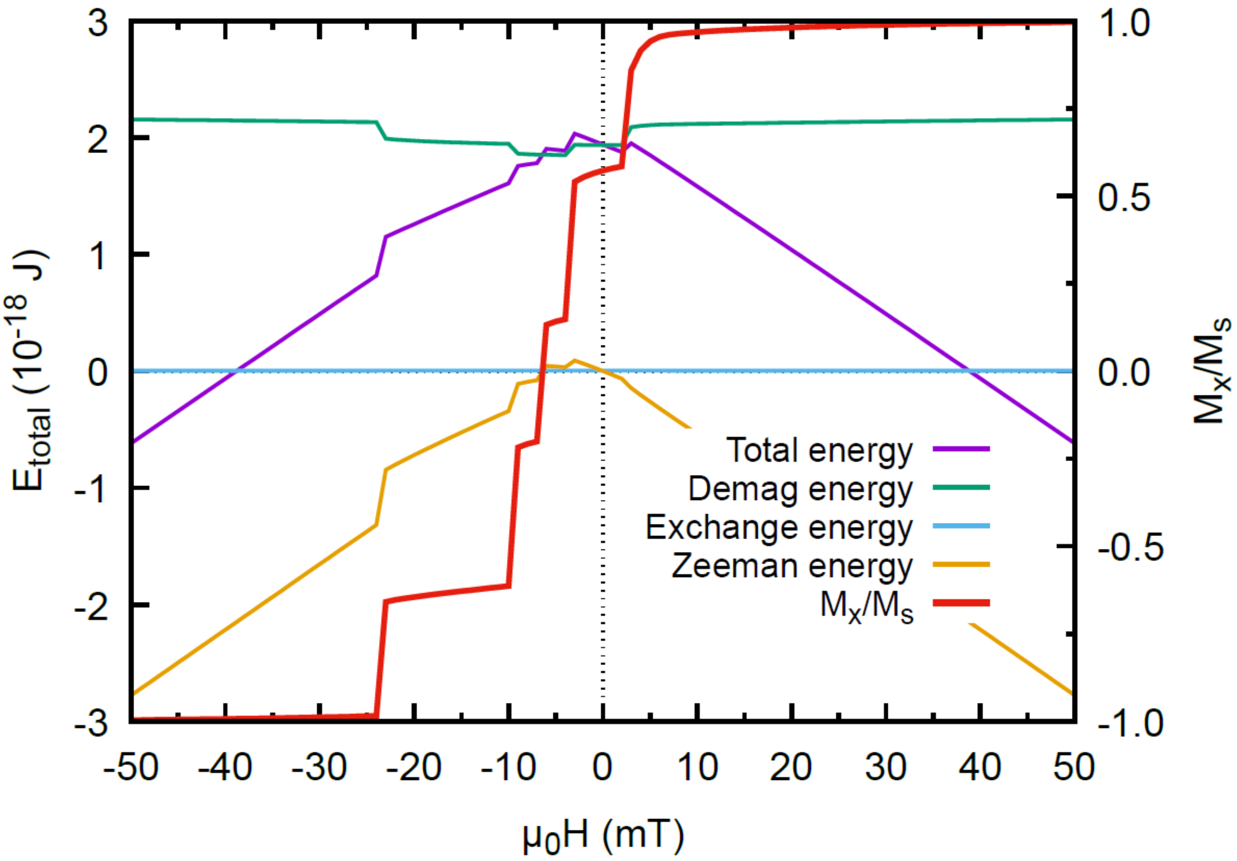

Figure SI-12 Total energy and individual energy terms are plotted on the left axis showing the relative contribution of each to the total. Also plotted (on the right axis) is the normalized magnetization, showing the partial reversal process for the 5 chain, N=27 hcp curve shown in Fig. 15.

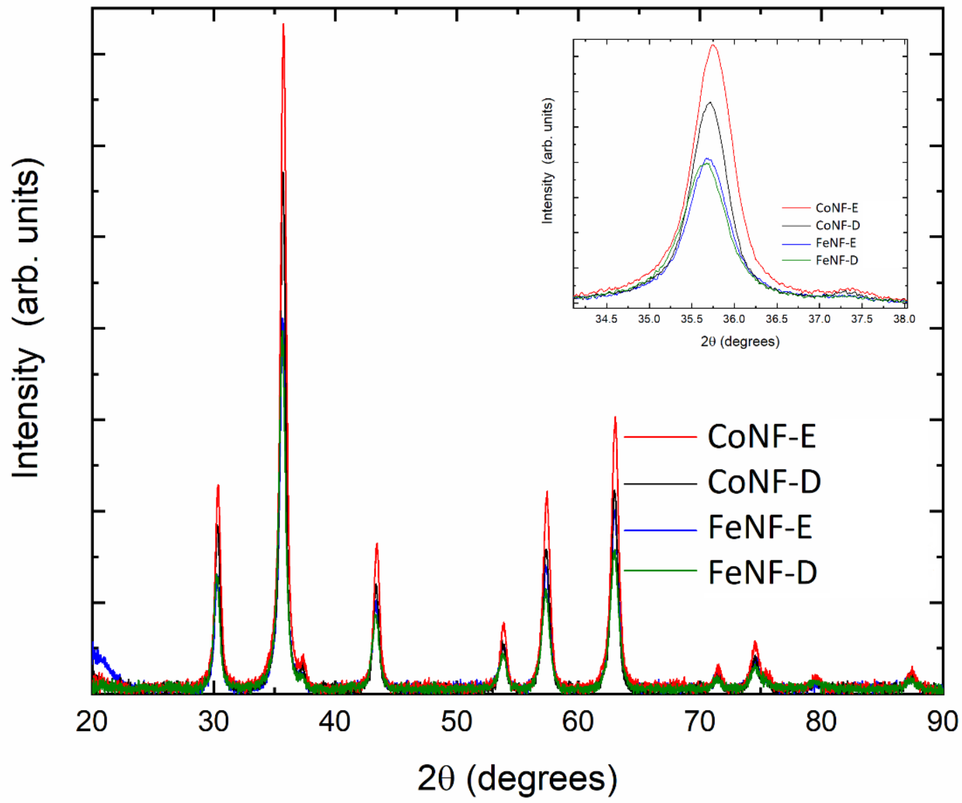

Figure SI-13 - Direct comparison of the XRD data for the four samples. Inset shows a close-up of the primary peak at 35.7 ^{o} in 2ϑ showing the slight shift of the CoNF peak relative to the FeNF peak.
